# Multiomic analysis reveals a key BCAT1 role in mTOR activation by B cell receptor and TLR9

**DOI:** 10.1172/JCI186258

**Published:** 2025-09-09

**Authors:** Rui Guo, Yizhe Sun, Matthew Y. Lim, Hardik Shah, Joao A. Paulo, Rahaman A. Ahmed, Weixing Li, Yuchen Zhang, Haopeng Yang, Liang Wei Wang, Daniel Strebinger, Nicholas A. Smith, Meng Li, Merrin Man Long Leong, Michael Lutchenkov, Jin Hua Liang, Zhixuan Li, Yin Wang, Rishi Puri, Ari Melnick, Michael R. Green, John M. Asara, Adonia E. Papathanassiu, Duane R. Wesemann, Steven P. Gygi, Vamsi K. Mootha, Benjamin E. Gewurz

**Affiliations:** 1 Division of Infectious Diseases, Department of Medicine, Brigham and Women’s Hospital, Boston, Massachusetts, USA.; 2 Center for Integrated Solutions in Infectious Disease, Broad Institute, Cambridge, Massachusetts, USA.; 3 Department of Microbiology and; 4 Department of Cell Biology, Harvard Medical School, Boston, Massachusetts, USA.; 5 Harvard Program in Virology, Boston, Massachusetts, USA.; 6 Howard Hughes Medical Institute and Department of Molecular Biology, Massachusetts General Hospital, Boston, Massachusetts, USA.; 7 Department of Medicine, Division of Allergy and Clinical Immunology, Brigham and Women’s Hospital and Harvard Medical School, Boston, Massachusetts, USA.; 8 Ragon Institute of Massachusetts General Hospital and; 9 Broad Institute of Harvard and Massachusetts Institute of Technology, Cambridge, Massachusetts, USA.; 10 Ergon Pharmaceuticals, LLC, Silver Spring, Maryland, USA.; 11 Department of Lymphoma/Myeloma, University of Texas MD Anderson Cancer Center, Houston, Texas, USA.; 12 Department of Medicine, Division of Hematology & Medical Oncology, Weill Cornell Medicine, New York, New York, USA.; 13 Department of Biomedical Sciences, College of Veterinary Medicine, Cornell University, Ithaca, New York, USA.; 14 Mass Spectrometry Core, Beth Israel Deaconess Medical Center, Boston, Massachusetts, USA.

**Keywords:** Cell biology, Metabolism, Adaptive immunity, Amino acid metabolism, Lymphomas

## Abstract

B lymphocytes play major adaptive immune roles, producing antibodies and driving T cell responses. However, how immunometabolism networks support B cell activation and differentiation in response to distinct receptor stimuli remains incompletely understood. To gain insights, we systematically investigated acute primary human B cell transcriptional, translational, and metabolomic responses to B cell receptor (BCR), TLR9, CD40-ligand (CD40L), IL-4, or combinations thereof. T cell–independent BCR/TLR9 costimulation, which drives malignant and autoimmune B cell states, highly induced transaminase branched chain amino acid transaminase 1 (BCAT1), which localized to lysosomal membranes to support branched chain amino acid synthesis and mTORC1 activation. BCAT1 inhibition blunted BCR/TLR9, but not CD40L/IL-4–triggered B cell proliferation, IL-10 expression, and BCR/TLR pathway–driven lymphoma xenograft outgrowth. These results provide a valuable resource, reveal receptor-mediated immunometabolism remodeling to support key B cell phenotypes, and identify BCAT1 as an activated B cell therapeutic target.

## Abstract 2

Primary human B-cells remodel distinct metabolism pathways to support responses to key immune stimuli. BCAT1 Supports T-cell Independent B-cell Activation

## Graphical abstract

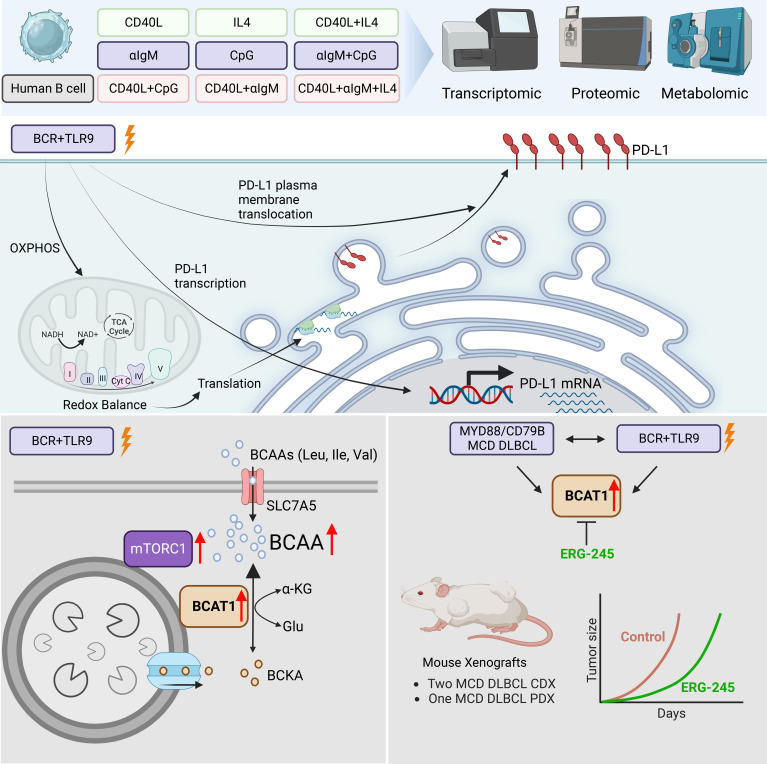

## Introduction

B cells decode a multitude of membrane receptor stimuli to decide whether and how to respond to myriad innate and adaptive immune stimuli. Collective B lymphocyte responses to receptor signals drive humoral and cell-mediated immune responses but also underlie autoimmune and B cell lymphoma disease states. In addition to their obligatory role in humoral immunity, B cells also carry out major immune functions, including initiating T cell responses, maintaining immune homeostasis, and driving tumor responses to checkpoint blockade (1, 2). Yet, much remains to be learned about how human B cell immunometabolism response to distinct T cell–dependent versus T cell–independent signals, received either alone or in combination, drive rapid immune responses.

B cells recognize a remarkable range of antigens via the cell surface B cell receptor (BCR), composed of immunoglobulin heavy and light chains and associated CD79a/Iga and CD79b/Igb signaling chains. After BCR activation, immunogens are internalized and processed in lysosomes, where peptide antigens are presented via MHC class II molecules to CD4^+^ T cells. In turn, activated CD4^+^ T cells can then provide crucial second signals to drive B cell activation, and two signals are generally needed to drive B cell proliferation (2). These include CD40-ligand (CD40L/CD154) and IL-4 (3). CD40L trimers activate cognate B cell plasma membrane CD40 receptors, which stimulate NF-κB, MAPK, and AKT/PI3K pathways (4, 5), whereas the IL-4 receptor (IL-4R) stimulates JAK/STAT pathways to drive B cell activation and differentiation (6). T cell CD40L and cytokine cues are critical for major B cell activities, including germinal center formation, class-switch recombination, and somatic hypermutation. Receipt of multiple activating signals rescues B cells from death (7) and induces rapid B cell proliferation, which serves to expand the pool of antigen-specific B cells and supports B cell differentiation and the formation of germinal centers.

B cells can also be activated by innate immune signals, including pathogen-associated molecular patterns (PAMPs) that are recognized by TLRs. TLR9 recognizes unmethylated CpG dinucleotides within endosomal compartments, where it then signals through the adaptor protein MyD88 to activate IL-1 receptor–associated kinases 1 and 4 to activate NF-κB, MAPK, and IFN regulatory factor pathways (8–13). PAMPs provide the adaptive immune system with an additional layer of self/nonself-discrimination (14, 15). TLR9 signaling, together with BCR stimulation, drives type 1 T cell–independent responses (2). By contrast, certain highly multivalent antigens trigger type 2 T cell–independent responses (16). However, TLR9 can downmodulate antigen presentation and disrupt affinity maturation downstream of BCR engagement (17). Gain-of-function CD79 and MyD88 mutations hyperactivate BCR/TLR9 signaling in several types of lymphoma, including the diffuse large B cell lymphoma (DLBCL) MyD88/CD79B-mutated (MCD) subtype (18–20). MCD DLBCLs are aggressive and typically have inferior clinical outcomes, highlighting the need for novel therapeutic approaches (19). In MCD DLBCL, TLR9/BCR coactivation drives the formation of the internalized MyD88-TLR9-BCR (My-T-BCR) complex, which hyperactivates mTOR from late endosomes (21). TLR signaling also plays key roles in B cell autoimmune responses, including in systemic lupus erythematosus (8, 22, 23). Interestingly, although TLR9 promotes loss of tolerance to DNA in lupus, it is protective against systemic lupus erythematosus through MyD88-independent roles (24, 25).

An open question is how immunometabolism networks support B cell activation, differentiation, rapid proliferation, and humoral responses to distinct stimuli. Whereas resting primary human B cells have low basal metabolism (26), B cells rapidly remodel metabolism pathways in response to receptor stimuli (7, 27, 28). For example, human B cells rapidly increase oxidative phosphorylation (OXPHOS) and glycolysis in response to BCR stimulation, but are unable to sustain this in the absence of T cell help or TLR9 costimulation (7). IL-4 costimulates B cell responses, in part through increasing the abundance of α-ketoglutarate (αKG), a key TCA intermediate and anaplerotic substrate (29).

To gain insights into ex vivo primary human B cell responses, we leveraged bulk transcriptomic, proteomic, and metabolomic approaches to characterize responses to 9 major routes of immune receptor stimulation. BCR/TLR9 coactivation highly induced the enzyme branched chain amino acid transaminase 1 (BCAT1) and drove its lysosomal subcellular localization, where it synthesized branched chain amino acids (BCAAs) to support mTOR activation critical for primary B cell growth and survival. A BCAT1 antagonist diminished outgrowth of BCR/TLR9-driven lymphomas in vivo, including a patient-derived xenograft (PDX).

## Results

### Multiomics profiling of differential primary human B cell responses to immune receptor stimuli.

**Figure 1:**
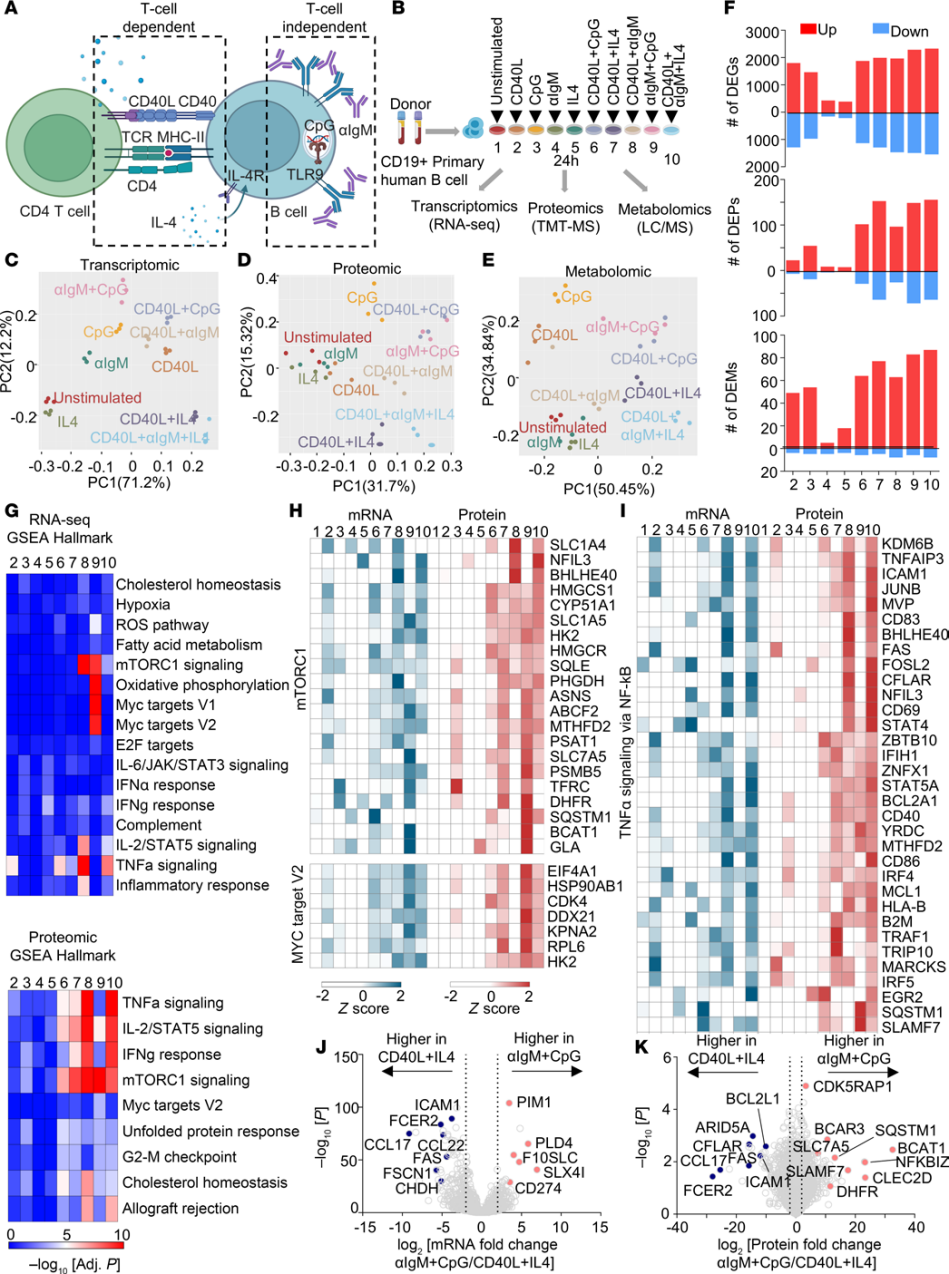
Receptor-driven B cell activation results in different cellular responses. (**A**) Schematic of T cell–dependent or –independent B cell activation pathways. (**B**) Multiomics profiling experimental design. Human primary peripheral blood CD19^+^ B cells were isolated by negative selection from 3 donors and stimulated by CD40L (50 ng/mL), CpG (0.5 μM), IL-4 (20 ng/mL), αIgM (1 mg/mL), or combinations thereof for 24 hours and then profiled. (**C**–**E**) Principal component analysis of transcriptomic (**C**), proteomic (**D**), and metabolomic (**E**) datasets. (**F**) Numbers of differentially expressed genes (DEGs), proteins (DEP), and metabolites (DEMs) across conditions using the numbering scheme in **B**, relative to unstimulated cells and using a *P* value < 0.01 and a fold-change > 2 or <0.5 cutoff. (**G**) GSEA of pathways enriched across conditions at the RNA (top) or protein (bottom) levels. (**H**) Heatmap visualization of row *z* scores of mRNA and protein abundance in GSEA Hallmark mTORC1 signaling (top) and MYC target V2 (bottom) gene set. (**I**) Heatmap visualization of row *z* scores of mRNA and protein abundance in GSEA TNF-α signaling via NF-κB gene set. (**J**) Volcano plot visualization of –log_10_ (*P* value statistical significance) versus log_2_ (mRNA abundance fold change) from RNA-Seq analysis of B cells stimulated with αIgM + CpG versus CD40L + IL-4. (**K**) Volcano plot visualization of –log_10_ (*P* value statistical significance) versus log_2_ (protein abundance fold change) from proteomic analysis of B cells stimulated with αIgM + CpG versus CD40L + IL-4.

To systematically investigate acute primary human B cell transcriptional, translational, and metabolomic responses to key T cell–dependent versus T cell–independent receptor cues, peripheral blood CD19^+^ cells were purified by negative selection. We modeled responses to key T cell–independent stimuli by anti-immunoglobulin cross-linking to drive BCR signaling and/or with the TLR9-activating PAMP CpG oligonucleotide. To model responses to key T cell–dependent stimuli, B cells were instead stimulated with trimeric CD40 ligand (CD40L) and/or IL-4 (Figure 1, A and B). We also activated B cells with CD40L + CpG to model bystander B cell activation or with CD40L + αIgM ± IL-4 to model antigen receptor stimulation with T cell help. To facilitate cross-comparison between agonists, we tested the effects of 5 different concentrations for each agonist to define a dosage that produced a maximal or near-maximal response (Supplemental Figure 1, A and B; supplemental material available online with this article; https://doi.org/10.1172/JCI186258DS1). We then profiled cells at 24 hours after stimulation, a time point prior to the first mitotic division of both naive B cells and circulating memory B cells (Supplemental Figure 2, A and B), in order to permit cross-comparison with the basal unstimulated state. Indicative of distinct biological outcomes, B cells exhibited markedly different morphological changes in response to these stimuli. For instance, B cells stimulated by αIgM + CpG appeared larger but formed smaller aggregates by comparison with cells stimulated by T cell–dependent signals (Supplemental Figure 3A).

In order to systematically investigate how individual versus combinatorial stimuli altered B cell expression and immunometabolism networks, we conducted parallel RNA-Seq, tandem-mass-tag mass spectrometry proteomic and polar metabolite liquid chromatography/mass spectrometry (LC/MS) profiling across all 10 conditions (Supplemental Table 1). Principal component analysis (PCA) yielded triplicates that closely clustered together from each condition, indicating a high level of reproducibility across conditions and human donors (Figure 1, C–E). Notably, transcriptomic responses to T cell–dependent stimuli modeled by CD40L + IL-4 ± αIgM clustered oppositely from responses to T cell–independent αIgM + CpG (Figure 1C).

We next cross-compared differentially expressed genes (DEGs), differentially expressed proteins (DEPs), and differentially expressed metabolites (DEMs) across the 10 conditions to broadly characterize stimulus-specific B cell responses. Response magnitude was generally higher at the transcriptomic than proteomic level at this early time point, with CD40L or CpG stimulation eliciting larger numbers of DEGs than stimulation by either αIgM or IL-4 alone (Figure 1F and Supplemental Figure 3B). Interestingly, similar numbers of DEGs were observed in response to CD40L, CpG, and to the combinatorial stimuli tested, suggesting dominant effects of these ligands. Combinatorial stimulation differentially regulated a large gene set, with 1,077 DEGs overlapping across all 5 combinatorial stimulation groups, despite these varying by the degree of T cell–dependent versus T cell–independent signaling (Supplemental Figure 3C).

Despite a degree of overlap, we nonetheless observed that each of the 9 stimulation conditions most dynamically regulated a small set of genes often implicated in B cell biology, most of which were upregulated (Supplemental Figure 4). These included well-characterized T cell–dependent genes upregulated by CD40/IL-4, including *AICDA*, which encodes the enzyme AID, and XBP1, which stimulates plasma cell differentiation (30). Interestingly, IL-4 stimulation alone most highly induced the transcriptional repressor BCL6, which is critical for germinal center formation and for preventing premature B cell activation and differentiation (31, 32) (Supplemental Figure 4). By contrast, T cell–independent αIgM + CpG costimulation selectively induced a wider range of targets, including the de novo nicotinamide adenine dinucleotide biosynthetic enzyme NAMPT and the neutral amino acid transporter SLC7A5 (Supplemental Figure 4). CpG alone, but to a greater extent αIgM + CpG, highly induced *PRDM1*, which encodes BLIMP1, the master regulator of antibody-secreting cell differentiation. Gene Set Enrichment Analysis (GSEA) (33) highlighted that αIgM + CpG stimuli more strongly induced mTORC1 signaling, MYC targets, and OXPHOS (group 9, Figure 1, G and H), suggesting that T cell–independent stimulation may preferentially upregulate these key immunometabolic pathways. By contrast, CD40L + IL-4 or CD40L + IL-4 + αIgM stimulation more strongly induced TNF-α, IL-2/STAT5, and IFN-γ signaling (group 7, Figure 1, G and I). These gene sets included transcription factors, metabolic enzymes, and cell surface receptors with critical roles in B cell adhesion, activation, survival, and antigen presentation (Figure 1H), highlighting multiple levels of T/B cell crosstalk.

To gain further insights into B cell responses to T cell–dependent versus T cell–independent stimuli, we next directly cross-compared the CD40L + IL-4 versus αIgM + CpG conditions. Volcano plot analysis highlighted CD40L + IL-4 induction of mRNAs encoding the T cell chemoattractant chemokines CCL17 and CCL22, exemplifying B/T cell cross-communication even at the early 24-hour time point. Transcripts encoding multiple B cell surface proteins were likewise more highly induced by T cell–dependent signaling, including FAS, ICAM-1, and CD23/FCER2. By contrast, αIgM + CpG more highly upregulated CD274, which encodes the immune checkpoint regulator PD-L1 (Figure 1J). On the protein level, CD40L + IL-4 more strongly upregulated the antiapoptotic proteins CFLAR/cFLIP and BCL2L1/Bcl-xL, even at this early time point, whereas αIgM + CpG more highly induced CLEC2D, the lectin receptor for the NK inhibitor receptor KLRB1. Taken together with effects on PD-L1, these results suggest that T cell–independent stimulation may downmodulate key cellular immune responses. Suggestive also of key T cell–independent effects on B cell immunometabolism, αIgM + CpG also more highly induced dihydrofolate reductase (DHFR), the neutral amino acid transporter (SLC7A5) and branched-chain amino acid transaminase 1 (BCAT1), which are key regulators of folate, amino acid, and BCAA metabolism, respectively (Figure 1, J and K).

### BCR and TLR9 coactivation hyperactivates key B cell immunometabolism pathways.

**Figure 2:**
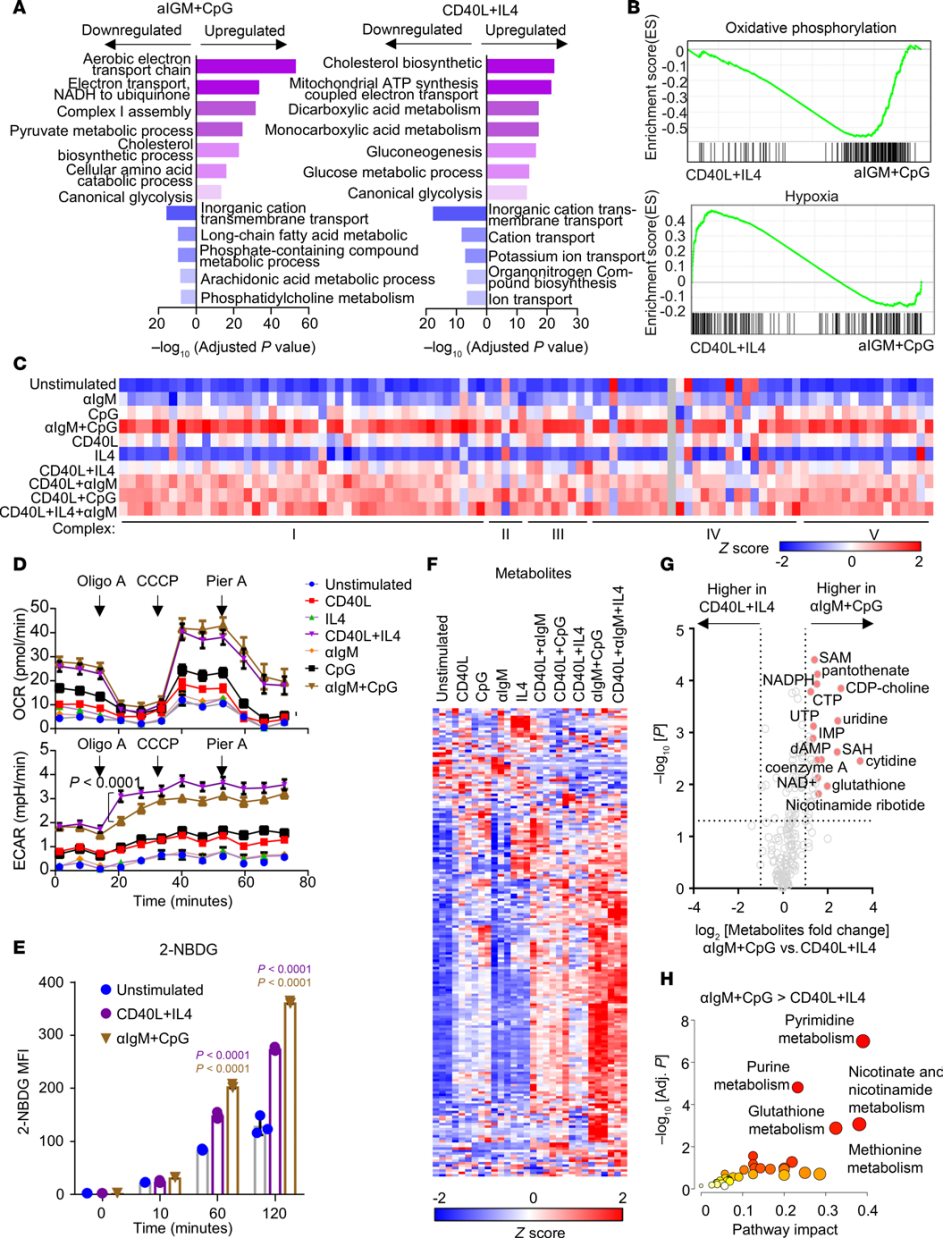
Cross-comparison of αIgM + CpG versus CD40/IL-4–driven B cell metabolism remodeling. (**A**) Gene ontology (GO) biological process analysis of genes differentially expressed in B cells stimulated with αIgM + CpG versus CD40L + IL-4, using a curated metabolism gene set (34). (**B**) GSEA Hallmark pathway analysis of oxidative phosphorylation (top) and hypoxia genes in CD differentially expressed genes in B cells stimulated with αIgM + CpG versus CD40L + IL-4. (**C**) Heatmap analysis of mRNA encoding electron transport chain (ETC) components in cells stimulated as indicated. Columns display *z* score values for each ETC gene, produced by cross-comparison of the 10 conditions. (**D**) Seahorse oxygen consumption rate (OCR, top) and extracellular acidification rate (ECAR, bottom) of primary B cells stimulated by indicated conditions for 24 hours and subject to flux analysis in the presence of indicated ETC inhibitors. Mean ± SEM from *n* = 7 replicates shown. *P* values calculated by 2-way ANOVA. (**E**) FACS analysis of primary B cell glucose analogue 2-(N-(7-nitrobenz-2-oxa-1,3-diazol-4-yl)amino)-2-deoxyglucose uptake at the indicated time points after stimulation. Mean ± SEM from *n* = 3 replicates. *P* values calculated by 2-tailed paired Student’s *t* test. (**F**) Heatmap analysis showing intracellular metabolite *z* scores in primary human B cells stimulated for 24 hours as indicated. (**G**) Volcano plot visualization of –log_10_ (*P* value statistical significance) versus log_2_ (fold-change metabolite abundance) from primary B cells stimulated by αIgM + CpG versus CD40L + IL-4 for 24 hours from *n* = 3 replicates. (**H**) MetaboAnalyst pathway enrichment analysis of metabolites that were higher in αIgM + CpG–stimulated cells than CD40L + IL-4–stimulated cells at 24 hours.

To gain further insights into how T cell–dependent versus independent stimuli affect primary B cell immunometabolism, we next analyzed metabolic gene responses using a curated gene set (34). As Figure 2A shows, αIgM + CpG and CD40/IL-4 similarly induced multiple metabolic pathways, albeit to varying degrees, indicating responses to somewhat overlapping metabolic demands. Yet, large clusters of metabolic genes were more highly induced by αIgM + CpG than by CD40L + IL-4 (Supplemental Figure 5A). Gene ontology (GO) analysis indicated that OXPHOS, fatty acid metabolism, purine metabolism, and amino acid catabolism were all more highly induced by αIgM + CpG than by CD40L + IL-4 (Figure 2, A and B, and Supplemental Figure 5, A and B).

Components of all 5 electron transport chain complexes were the most highly upregulated by αIgM + CpG, whereas stimulation by a CD40L-containing regimen induced these to a lesser extent (Figure 2C). Phenotypically, Seahorse metabolic flux analysis identified that αIgM + CpG and CD40L + IL-4 nonetheless similarly induced basal respiration and maximal respiratory capacity, perhaps suggesting posttranscriptional level compensatory regulation (Figure 2D). Consistent with prior analyses (7), combinatorial αIgM + CpG stimuli more strongly induced oxygen consumption rate (OCR) than either αIgM or CpG alone. A similar phenomenon was observed with CD40L and IL-4 stimuli (Figure 2D). Of the transcription factors implicated in control of respiratory chain component expression, MYC was the most highly induced by CD40L and a regimen containing CD40L, although combinatorial αIgM + CpG also highly induced MYC (Supplemental Figure 5C). Notably, CD40 + IL-4 costimulation elicited a higher extracellular acidification rate, a measure of glycolysis (Figure 2D). Taken together with our GSEA, which identified hypoxia gene upregulation in CD40L/IL-4–stimulated cells (Figure 2B), these results suggest that T cell–dependent B cell responses may be more reliant on aerobic glycolysis. However, while CD40L/IL-4 and αIgM/CpG stimulation each significantly increased glucose uptake, αIgM + CpG did so more strongly (Figure 2E).

Across individual stimuli, CD40L or CpG more highly affected the intracellular B cell metabolite landscape than either αIgM or IL-4, consistent with the magnitude of their transcription level effects. As shown in Figure 2F, αIgM + CpG produced the strongest cellular metabolome-wide effect, which triggered higher metabolite levels of the purine and pyrimidine nucleotide, methionine, nicotinate/nicotinamide, and glutathione metabolism pathways (Figure 2, G and H, and Supplemental Table 1). Despite these differences, combinatorial CD40L + IL-4 + αIgM stimulation, which models a key T cell–dependent germinal center light zone B cell stimulus, produced somewhat overlapping metabolomic responses with T cell–independent αIgM + CpG stimulation (Figure 2F and Supplemental Figure 5, A and B). Collectively, these findings highlight that distinct types of receptor stimuli, including a T cell–dependent versus T cell–independent regimen, produce myriad metabolomic responses in primary human CD19^+^ B cells, with potential major effects on key humoral immune phenotypes.

Multiple targets induced by αIgM/CpG stimulation, such as ASNS and MTHFD2, are targets of the activating transcription factor 4 (ATF4), which serves as a central mediator of cellular responses to stress, including metabolic adaptation (35). For instance, ATF4 drives integrated stress responses by promoting expression of cytoprotective genes that program key metabolic pathways (36, 37). We therefore further investigated agonist responses on ATF4 expression. Transcriptomic analyses identified that multiple stimuli, including αIgM + CpG and CD40L + IL-4, increased ATF4 mRNA levels by approximately 2-fold (Supplemental Figure 6A). ATF4 upregulation on the protein level was also observed by immunoblot (Supplemental Figure 6B). Given ATF4 roles in metabolic adaptation, we tested whether ATF4 induction was mTOR dependent. Indeed, blockade of mTORC1 and 2 by the small molecule antagonist Torin 1 prevented ATF4 induction in αIgM/CpG-stimulated Rael Burkitt B cells (Supplemental Figure 6C).

### BCAT1 is essential for BCR/TLR9-driven B cell proliferation.

**Figure 3:**
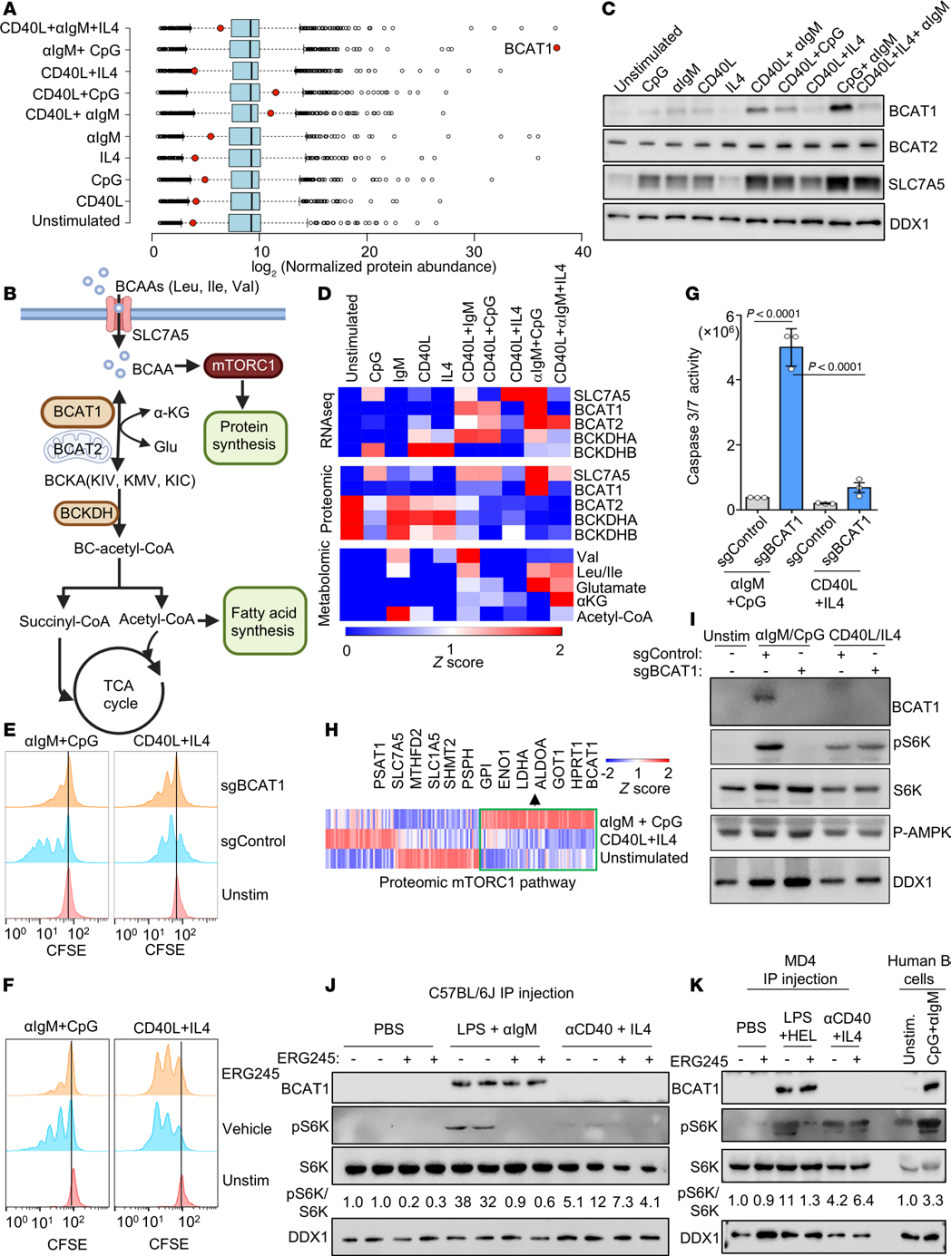
BCR/TLR9 costimulation highly induces BCAT1, which is essential for αIgM/CpG but not CD40/IL-4–driven primary B cell mTORC1 activation, growth, and survival. (**A**) Log_2_-normalized protein abundance from proteomic analysis of primary B cells after 24-hour stimulation. BCAT1 protein levels are highlighted in red. (**B**) BCAA metabolism schematic. Cytosolic BCAT1 and mitochondrial BCAT2 catalyze reversible transamination of BCKA and BCAA.. (**C**) Immunoblot of BCAT1, BCAT2, SLC7A5, and DDX1 from whole-cell lysates (WCLs) of 24-hour stimulated B cells. DDX1 was the load control. (**D**) Heatmap analysis of key BCAA pathway RNA, protein, and metabolite z scores in 24-hour stimulated primary B cells. (**E**) CFSE analysis of primary B cells electroporated with Cas9 ribonucleoprotein complexes with control or BCAT1 targeting sgRNA, then stimulated for 5 days. (**F**) CFSE analysis of primary B cells treated with vehicle or ERG245 (100 mM) and stimulated. (**G**) Mean ± SEM caspase-3/7 activity of primary B cells with indicated Cas9 ribonucleoproteins after 48 hour stimulation. *P* values calculated by 2-tailed paired Student’s *t* test. (**H**) Heatmap of selected mTORC1 pathway target gene z scores, shown in vertical columns, in 24-hour stimulated primary B cells. Metabolic enzymes upregulated by αIgM + CpG vs. CD40L + IL-4 stimulation are highlighted. (**I**) Immunoblots of WCLs from 24-hour stimulated primary B cells expressing indicated Cas9 ribonucleoproteins. (**J**) Immunoblot of WCLs from C57BL/6J murine splenic B cells, harvested 48 hours after i.p. injection of PBS vehicle, 20 mg LPS + 250 mg αIgM, or 250 mg aCD40 antibody + 1 mg murine IL-4 in complex with 10 mg anti–IL-4, with vehicle or 20 mg/kg ERG245. (**K**) Immunoblot of WCLs from MD4 murine splenic B cells, harvested 48 hours after i.p. injection of PBS vehicle, 20 mg LPS + 10 mg HEL, or 250 mg aCD40 antibody + 1 mg IL-4 in complex with 10 mg αIL-4, with vehicle or 20 mg/kg ERG245. Representative of *n* = 3 replicates (**E**, **F**, and **I**–**K**). See also Supplemental Figure 4.

We observed that BCR/TLR9 costimulation markedly upregulated BCAT1 expression on the mRNA and protein levels, whereas it was induced to a much lesser extent or not at all by the other stimuli (Figure 3A and Supplemental Figure 7A). BCAT1 is an aminotransferase that can either synthesize or catabolize the BCAAs leucine, isoleucine, and valine in reversible reactions, but it has not previously been studied in B cell activation. When running in the forward direction, BCAT1 converts the nitrogen donor glutamine and branched chain ketoacids (BCKAs) into αKG and BCAA, which support protein synthesis and mTOR activation. When running in the reverse direction, BCAT1 instead catabolizes αKG and BCAA to produce glutamine and BCKA, which fuel TCA and fatty acid synthesis (38–40).

We validated that αIgM + CpG more strongly upregulated BCAT1 by immunoblot (Figure 3C). By comparison, the mitochondrial BCAT2 isoform was expressed in unstimulated cells and was only modestly upregulated by any of the conditions (Figure 3, C and D). BCAT1 was induced by that αIgM + CpG in both peripheral blood CD27^–^ naive B cells and in CD27^+^ circulating memory cells (Supplemental Figure 7B). Consistent with a key BCAA role in support of BCR/TLR9-costimulated B cells, the major plasma membrane BCAA transporter SLC7A5 was also highly induced (Figure 3, B–D). BCAA abundance was also higher in αIgM + CpG–stimulated cells than in B cells stimulated by combinatorial regimens, with the exception of CD40L + αIgM + IL-4. By contrast, subunits of the BCKA dehydrogenase complex (BCKDHA/B), which participate in BCKA catabolism to acetyl-CoA and CO_2_, were downmodulated on the protein level (Figure 3D). This raised the interesting possibility that BCAT1 may take on a selectively important role downstream of CD79 and MyD88.

To gain insights into BCAT1 roles in B cell activation, we tested the effects of BCAT1 perturbation on proliferation and survival of peripheral blood B cells stimulated by αIgM + CpG versus by CD40L + IL-4. We electroporated freshly isolated B cells with Cas9 ribonucleoprotein complexes containing control or *BCAT1* targeting sgRNA (Supplemental Figure 7C) (41). Intriguingly, CRISPR *BCAT1* KO strongly impaired αIgM + CpG– but not CD40L + IL-4–driven primary B cell outgrowth, as judged by a CFSE dye dilution assay (Figure 3E and Supplemental Figure 7D). Similar results were obtained with primary B cells treated with the highly selective leucine-based BCAT1 small molecule antagonist ERG245 (42), suggestive of on-target effects at the level of BCAT1 (Figure 3F and Supplemental Figure 7, E and F). We further validated ERG245 on-target effects by LC-MS analysis (Supplemental Figure 8 and Supplemental Table 2). In particular, ERG245 treatment produced similar results to BCAT1 KO in Rael Burkitt B cells, which we used to achieve higher levels of CRISPR editing than we could achieve in primary B cells (Supplemental Figure 8, A–C). To interrogate effects of BCAT1 KO on primary B cell survival, we next performed caspase activity assays. BCAT1 KO induced caspase-3/7 activity and cell death to a significantly greater extent in αIgM + CpG–stimulated than in CD40L + IL-4–stimulated primary B cells (Figure 3G and Supplemental Figure 9A).

We hypothesized that BCAT1 may be needed to support mTOR in αIgM + CpG–stimulated cells, given that αIgM + CpG most highly activated GSEA Hallmark mTORC1 signaling at the RNA and protein levels (Figure 1G). In support, proteomic analysis highlighted that clusters of mTORC1 pathway targets were more highly upregulated by αIgM + CpG than by CD40L/IL-4 stimulation, including multiple components of the glycolysis, one-carbon metabolism, and amino acid metabolism pathways (Figure 3H). Consistent with this result, mTORC1 target S6K phosphorylation levels were higher in αIgM + CpG–stimulated cells, and BCAT1 KO strongly impaired S6K phosphorylation (Figure 3I). BCAT1 was similarly important for αIgM + CpG–driven phosphorylation of mTORC1 serine 2448, which is indicative of mTOR activation. Additionally, immunoblot analysis revealed that αIgM + CpG induced a much higher level of phospho-S6, further indicating hyperactivated mTORC1 signaling. Although phosphorylation of the mTOR-negative regulator AMPK was somewhat higher in αIgM + CpG–stimulated cells, this was not affected by BCAT1 KO, suggesting alternative route(s) by which BCAT1 supports mTOR. Since mTORC1 regulates translation, we also tested the translation rate using puromycin pulse labeling, which detects puromycin incorporation into elongating protein chains (43). Puromycin labeling indicated that αIgM + CpG more highly induced nascent polypeptide synthesis than the other conditions (Supplemental Figure 9B). Similar results were observed by flow cytometry analysis of total protein content and cell size, which is also controlled by mTORC1 (44). However, total protein content was slightly higher in cells stimulated by CD40L + αIgM + IL-4 (Supplemental Figure 9C). These findings indicate that BCAT1 is a major positive regulator of mTOR in αIgM + CpG–stimulated B cells.

### BCR/TLR9 costimulation induces BCAT1 in vivo.

To investigate whether BCAT1 is induced by BCR/TLR costimulation in support of mTOR activation in vivo, we utilized murine models. Since LPS is more commonly used than CpG to activate TLR signaling in mice, we first cross-compared BCAT1 induction in ex vivo murine splenic B cells stimulated by LPS + αIgM versus CpG + αIgM. Although both induced BCAT1, LPS + αIgM stimulation did so more strongly (Supplemental Figure 10A). We then stimulated C57BL/6J mice by i.p. injection of PBS vehicle, LPS + αIgM, or agonistic aCD40 mAb BE0016-2, together with IL-4, which we complexed with the mAb 11B11 to stabilize IL-4 in vivo (45, 46), in the absence or presence of ERG245. Consistent with our human B cell results, BCAT1 expression was highly induced on the protein level in murine splenic B cells by αIgM + LPS but not by CD40 + IL-4 stimulation (Figure 3J). Although ERG245 did not appreciably alter BCAT1 induction, it blocked αIgM + LPS–driven mTOR activation, as judged by S6K phosphorylation (Figure 3J).

To further test BCAT1 induction and support of mTOR activation in vivo in the context of physiological BCR stimulation, we next utilized MD4 transgenic mice (47), which express BCR directed against hen egg lysosome (HEL). As observed with C57BL/6J mice above, BCAT1 was highly induced by incubation of ex vivo MD4 splenic B cells with HEL together with LPS, but not appreciably by negative control ovalbumin or by CD40L + IL-4 stimulation (Supplemental Figure 10B). We then stimulated MD4 mice by i.p. injection with LPS + HEL or by aCD40 + IL-4 (stabilized by aIL-4 as above) for 48 hours, together with vehicle or ERG245. BCAT1 expression was again highly induced by BCR stimulation by HEL, together with LPS, but not appreciably by aCD40 + IL-4 stimulation. Importantly, LPS + HEL strongly induced S6K phosphorylation in a manner that was nearly completely blocked by ERG245, whereas S6K phosphorylation downstream of aCD40 + IL-4 was not diminished by ERG245 (Figure 3K). Together, these results indicate that BCAT1 is highly induced by combinatorial BCR + TLR stimulation in vivo, where it again plays key roles in support of mTOR activation.

### Analysis of mediators that induce BCAT1 and of B cell genes regulated by BCAT1.

To gain insights into pathways that induce BCAT1 expression, we stimulated human peripheral blood B cells by αIgM + CpG in the presence of DMSO vehicle versus well-characterized small molecule inhibitors against downstream BCR and TLR9 mediators. Inhibition of the kinases SYK or BTK by R406 or ibrutinib, respectively, each strongly impaired BCAT1 induction, suggesting the importance of BCR proximal tyrosine kinase signaling in driving BCAT1 expression (Supplemental Figure 11A). Inhibition of PI3 kinase by idelalisib blocked BCAT1 induction to a similar extent, likewise implicating kinase signaling downstream of TLR9 (Supplemental Figure 11A). By contrast, inhibition of NFAT, canonical NF-κB, or JAK/STAT only modestly impaired BCAT1 induction (Supplemental Figure 11A). Interestingly, MAPK and mTOR are activated downstream of both BCR and TLR9, and inhibition of ERK, JNK, or p38 MAPK or mTOR each strongly impaired BCAT1 induction (Supplemental Figure 11A). Taken together, these data highlight roles of kinase signaling in rapid BCAT1 induction by BCR + TLR9 costimulation.

To next characterize potential BCAT1 roles in transcription regulation downstream of BCR and TLR9, we performed RNA-Seq on αIgM + CpG–stimulated BCAT1 CRISPR KO versus control primary human B cells. BCAT1 KO upregulated 145 and downregulated 101 B cell genes at 24 hours of stimulation. GO analysis indicated that BCAT1 depletion resulted in downregulation of E2F targets and G2-M checkpoint genes in αIgM + CpG–stimulated cells (Supplemental Figure 11B and Supplemental Table 3). The most highly downregulated genes included IL-10; the DNA methylation enzyme UHRF1; the transcription factor BATF; and the genes CDC25A, MCM10, and PCNA, each of which have key cell cycle roles (Supplemental Figure 11C). We validated that αIgM + CpG induced IL-10 on the protein level in primary B cells. Furthermore, BCAT1 KO reduced IL-10 abundance to levels observed in CD40L/IL-4–stimulated cells, in which BCAT1 KO did not substantially alter IL-10 levels (Supplemental Figure 11D). Interestingly, BCAT1, IL-10, and PD-L1 mRNA amounts were each markedly higher at 48 hours of αIgM/CpG than in CD40L/IL-4–stimulated cells (Supplemental Figure 11E). Since IL-10 is a B regulatory cell hallmark (48–52), our data raise the possibility that BCAT1 may support B regulatory cell function upon BCR/TLR9 activation.

### BCAT1 supports BCAA production in BCR/TLR9-stimulated B cells.

**Figure 4:**
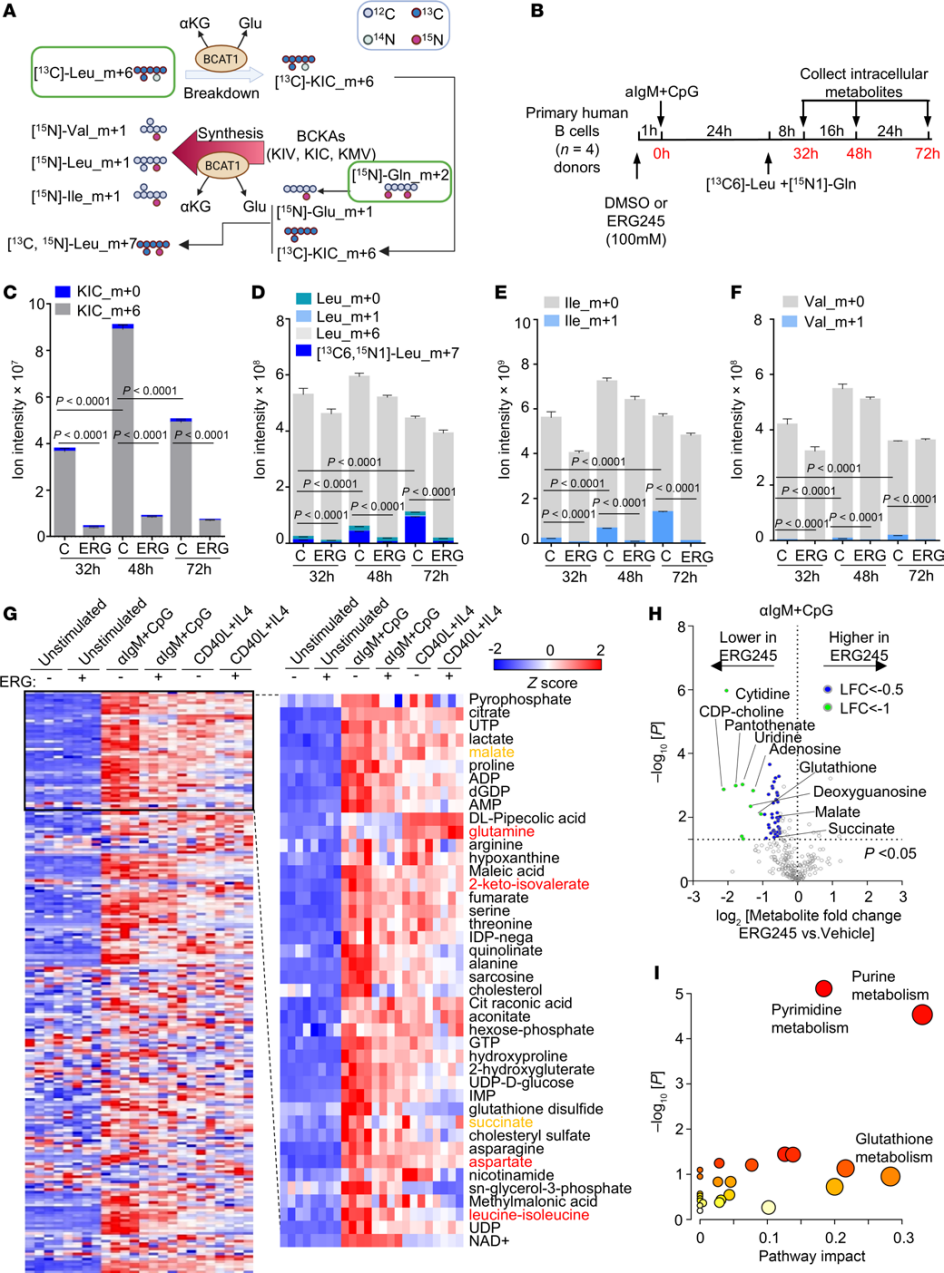
BCR/TLR9 stimulation drives BCAA synthesis in human primary B cells. (**A**) Isotope tracing schematic. [^13^C]-L-leucine_m+6 was used to trace BCAT1 BCAA catabolism to a-keto-isocaproic acid (KIC); [^15^N]-glutamine_m+2 was used to monitor BCAT1 BCAA biosynthesis from KIC, a-keto-isovaleric acid (KIV), or a-keto b-methylvaleric acid (KMV). Gln, glutamine. Glu, glutamate. (**B**) Isotope tracing experimental design. Primary B cells from *n* = 4 donors were pretreated with vehicle or ERG245(100 μM) for 1 hour and then stimulated by αIgM + CpG for 24 hours in the presence of vehicle or ERG245. Cells were washed with PBS 3 times and resuspended in glutamine/leucine-free media supplemented with 381 mM ^13^C6-leucine and 2.054 mM ^15^N2-glutamine + 10% dialyzed FBS. ERG245(100 μM) and αIgM + CpG stimulants were also refreshed at this time point. Intracellular metabolites were profiled at 8, 24, and 48 hours later. (**C**) Ion intensities of m+6-labeled and -unlabeled KIC at the indicated times in cells treated with vehicle control or ERG245. (**D**) Ion intensities of labeled and unlabeled leucine (Leu) at the indicated times in cells treated with vehicle control or ERG245. (**E**) Ion intensities of labeled and unlabeled isoleucine (Ile) at the indicated times in cells treated with vehicle control or ERG245. (**F**) Ion intensities of labeled and unlabeled valine (Val) at the indicated times in cells treated with vehicle control or ERG245. (**G**) Heatmap analysis of metabolite *z* scores in primary B cells treated with vehicle or ERG245 (100 μM) and stimulated as indicated for 24 hours. (**H**) Volcano plot visualization of –log_10_ (*P* value statistical significance) and log_2_ (fold-change metabolite abundance) from metabolomic analysis of ERG245-treated versus vehicle-treated primary B cells stimulated by αIgM + CpG for 24 hours from *n* = 4 replicates. (**I**) MetaboAnalyst pathway enrichment analysis of metabolites diminished by ERG245 treatment in αIgM + CpG–stimulated B cells at 24 hours. *P* values were calculated by 2-way ANOVA followed by Tukey’s multiple-comparison test (**C**–**F**).

We next used [^13^C]-leucine_m+6 and [^15^N]-glutamine_m+2 isotope tracing to investigate the directionality of BCR/TLR9-induced BCAT1 BCAA metabolism. To trace BCAT1 conversion of BCAA and αKG to BCKA and glutamate (Glu), we incubated αIgM + CpG–stimulated cells with 0.381mM [^13^C]-leucine_m+6 to survey for the appearance of labeled BCKA catabolites. However, since BCKA can then be reaminated by BCAT1 using glutamine (Gln) as the amino donor, we also added 2 mM [^15^N]-Gln_m+2. We measured BCAA catabolism by detecting [^13^C]-ketoisocaproate (KIC)_m+6 levels. Likewise, we measured BCAA anabolism by detecting the appearances of [^15^N]-labeled leucine (Leu)_m+1, isoleucine (Ile)_m+1, and valine (Val)_m+1 (Figure 4A). B cells were pretreated with either vehicle or ERG245 for 1 hour before αIgM + CpG stimulation, and then incubated in medium containing [^13^C]-Leu_m+6 and [^15^N]-Gln_m+2 starting from 24 hours after stimulation. Labeled and unlabeled metabolite abundance was quantitated at 32, 48, and 72 hours after stimulation (Figure 4B).

Liquid chromatography/mass spectrometry metabolite tracing indicated that BCAT1 does contribute to B cell BCKA pools upon its induction by αIgM + CpG, as [^13^C]-KIC_m+6 levels significantly increased between 32 and 72 hours after stimulation. [^13^C]-KIC_m+6 levels then decreased at the 72-hour time point, potentially indicating a balance between production and consumption (Figure 4C and Supplemental Table 4). BCAT1 inhibition by ERG245 strongly decreased [^13^C]-KIC_m+6 levels at all time points, indicating BCAT1 roles in KIC generation (Figure 4C). However, we also observed steadily increasing [^15^N]-labeled Leu_m+1, Ile_m+1, and to a lesser extent Val_m+1 levels, each of which were suppressed by ERG245, indicating that BCAT1 also consumes glutamine to synthesize BCAA and produce αKG (Figure 4, D–F). [^13^C,^15^N]-Leu_m+7 composed the majority of the [^15^N]-labeled Leu pool, indicating that BCAT1 preferentially reaminated [^13^C]-KIC_m+6 at this time point (Figure 4D). However, the fraction of [^15^N]-labeled Val_m+1 was comparatively smaller, suggesting that BCAT1 may preferentially synthesize Leu and Ile at the early stage of αIgM + CpG–driven B cell activation (Figure 4, D–F). In contrast to serving as a substrate for BCAA biosynthesis, [^13^C]-KIC_m+6 was not a major TCA cycle anaplerotic input. TCA intermediate m+2 isotope signals remained low throughout the time course, despite marked increases in unlabeled TCA metabolite abundance (Supplemental Figure 12, A–G). Intriguingly, ERG245 nonetheless strongly decreased levels of most TCA cycle intermediates, indicating that BCAT1 plays a crucial role in coordinating TCA metabolism in BCR/TLR9 coactivated cells, potentially via effects on mTOR (Supplemental Figure 12, A–G).

To broadly profile BCAT1 contributions to αIgM + CpG versus CD40L/IL-4 coactivated peripheral blood B cells, we performed LC/MS metabolome profiling on cells stimulated in the presence of DMSO vehicle control or ERG245. Whereas ERG245 had minimal effects on unstimulated cells, ERG245 broadly restrained BCR/TLR9-driven metabolite increases. Comparatively smaller effects were observed on CD40L/IL-4–treated cells, and ERG245 only modestly affected the metabolome (Figure 4G and Supplemental Table 5). Leucine-isoleucine and 2-keto-isovalerate levels were higher in αIgM + CpG–stimulated cells, whereas glutamine was higher in CD40/IL-4–stimulated cells (Figure 4G and Supplemental Figure 12H). Notably, ERG245 did not significantly change aspartate abundance, despite it being a substrate for similar transamination reactions (Supplemental Figure 12I). Volcano plot and metabolism pathway impact analysis highlighted that ERG245 most strongly reduced the abundance of nucleotides and glutathione in BCR/TLR9-stimulated B cells, potentially due to effects at the level of mTOR (Figure 4, H and I) and reflecting that resting human B cells have low nucleotide and glutathione levels and must rapidly increase them upon activation (26). These findings suggest that BCAT1 supports BCAA pools and mTOR upon BCR/TLR9-driven B cell activation.

Since import can also substantially affect BCAA intracellular levels, we next inhibited the plasma membrane neutral transporter LAT3 (also called SLC43A1), which is a major transporter of neutral amino acids including leucine, isoleucine, and valine. LAT3 inhibition by the highly selective small molecule antagonist Venuloside A (53) did not appreciably affect proliferation of αIgM + CpG–stimulated peripheral blood B cells. However, Venuloside A LAT3 inhibition impaired αIgM + CpG–driven peripheral blood B cell proliferation when dosed in combination with either of two ERG245 doses, to a significantly greater degree than ERG245 alone at either dose (Supplemental Figure 13). These results are consistent with a model in which BCAT1 inhibition renders cells more dependent on LAT3-mediated BCAA uptake.

### BCR/TLR9 signaling targets BCAT1 to lysosome membranes to support mTOR.

**Figure 5:**
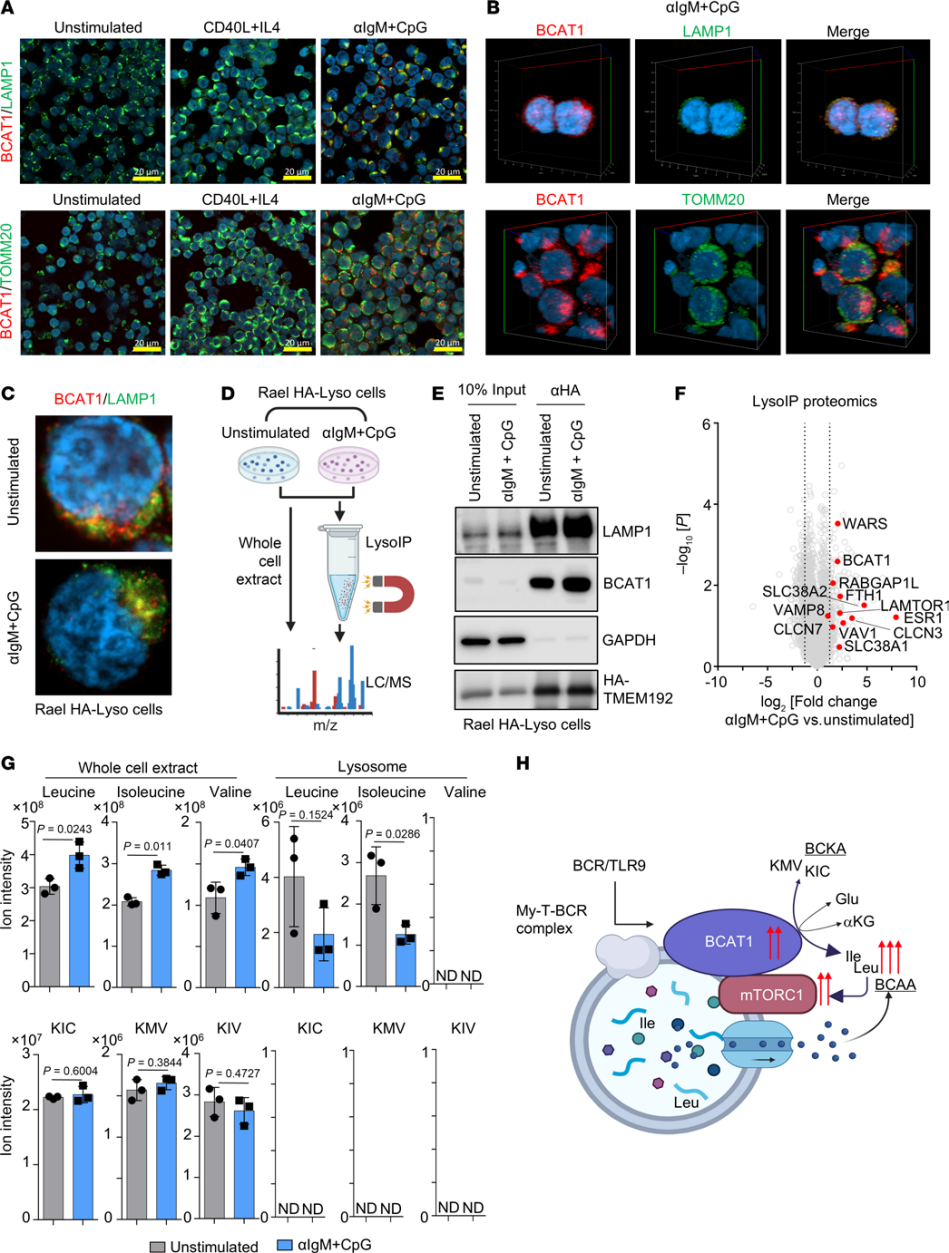
BCR/TLR9 but not CD40/IL-4 costimulation targets BCAT1 to remodeled lysosomes. (**A**) Confocal microscopy analysis of BCAT1 (red) colocalization with the lysosomal LAMP1 (top, green) or mitochondrial TOMM20 (bottom, green) markers in primary B cells stimulated for 24 hours as indicated. (**B**) 3D *Z*-stack reconstruction of BCAT1, LAMP1, and TOMM20 in primary B cells stimulated by αIgM + CpG for 24 hours. (**C**) Confocal analysis of BCAT1 and LAMP1 colocalization in Rael TMEM192-HA^+^ B cells (HA-Lyso cells) stimulated by αIgM + CpG for 24 hours, as indicated. (**D**) Lyso-IP proteomic analysis workflow. (**E**) Immunoblot of whole-cell lysates or anti-HA immunopurified lysosomes from Rael Lyso cells stimulated as in **D**. (**F**) Volcano plot of –log_10_ (*P* value) versus log_2_ (fold-change) of tandem-mass-tag protein abundance in immunopurified lysosomes from Rael Lyso-IP cells as in **D**. (**G**) Normalized BCAAs and BCKA ion intensities in whole-cell lysates versus lysosomes immunopurified from Rael Lyso-IP cells as in **D**. *P* values were calculated by 2-tailed paired Student’s *t* test. (**H**) Schematic of BCAT1 lysosomal targeting and BCAA production to support mTORC1 hyperactivation.

A complex of lysosomal membrane proteins sense amino acid levels to control mTORC1 recruitment and activation. When amino acids are abundant, mTORC1 is recruited to the outer lysosomal membrane, where it is activated by RHEB (54–57). When leucine levels are low, several mechanisms block mTORC1 activation. Sestrin2 inhibits mTORC1 lysosomal recruitment and activation (58), SAR1B inhibits the mTORC1 activator GATOR2(59), leucyl-tRNA synthetase LARS fails to activate Rag GTPase (60), and Raptor acetylation decreases to further downmodulate mTORC1(61). Because BCAT1 is thought to be cytoplasmic, we hypothesized that BCR/TLR9-induced BCAT1 supports mTORC1 by producing BCAA in close proximity with lysosomes. To test this, we performed confocal microscopy on primary B cells at rest or 24 hours after stimulation by CD40L/IL-4 versus αIgM + CpG, which revealed a high degree of colocalization between BCAT1 and lysosomal-associated membrane protein 1 (LAMP1) but not with the mitochondrial marker translocase of outer mitochondrial membrane 20 homolog (TOMM20) (Figure 5A and B, and Supplemental Figure 14, A–C). Thus, a major population of BCAT1 homes to lysosomes in BCR/TLR9-stimulated primary human B cells, presumably to the lysosomal outer membrane as BCAT1 does not appear to contain a targeting sequence for lysosomal uptake.

To further investigate BCAT1 subcellular localization, we then leveraged the LysoIP approach, in which stably expressed HA-epitope tagged transmembrane protein 192 (TMEM192) is used as a bait for lysosomal affinity purification (62) (Figure 5, C and D). We stably expressed TMEM192 in Rael Burkitt lymphoma B cells, in which BCAT1 and LAMP1 colocalization was increased by αIgM + CpG stimulation (Supplemental Figure 14D). The lysosomal marker LAMP1 was enriched in material anti–HA-TMEM192 immunopurified from Rael cells, whereas cytosolic GAPDH was highly depleted, suggesting successful lysosome isolation. Importantly, BCAT1 was also enriched in immunopurified lysosomes, particularly after αIgM + CpG stimulation, even though in Rael cells total BCAT1 levels remained similar (Figure 5E). Confocal microscopy again highlighted that αIgM + CpG increased BCAT1/LAMP1 colocalization in Rael TMEM192^+^ cells (Supplemental Figure 14D). BCAT1/LAMP1 colocalization was similarly evident in HBL1 MCD DLBCL cells (Supplemental Figure 14, E and F). These results suggest that BCR/TLR9 signaling contributes to BCAT1 lysosomal subcellular localization, likely at the outer membrane.

To gain further insights into how BCR/TLR9 stimulation remodels Rael lysosomes, we performed LC/MS proteomic profiling of lysosomes immunopurified from αIgM + CpG versus unstimulated LysoIP Rael cells. Consistent with our immunoblot and microscopy analyses, BCAT1 was enriched in lysosomes purified from stimulated Rael cells (Figure 5F and Supplemental Table 6). Intriguingly, BCR/TLR9 signaling also increased lysosomal levels of LAMTOR1, which has a key role in assembly of the Ragulator complex that together with the Rag GTPases control mTORC1 lysosomal recruitment (63, 64). Further suggestive of crosstalk between BCR/TLR9 and mTORC1 at the level of the lysosome, SLC38A1/2 abundance was also increased in lysosomes of stimulated Rael cells. SLC38A1/2 are membrane transporters that specialize in the uptake of neutral amino acids and that are implicated in mTORC1 regulation in T cells (65), but they have not yet been studied in B cells (Figure 5F and Supplemental Table 6). Of note, SLC38A2 family member SLC38A9 is a lysosomal membrane protein that is a major regulator of lysosomal amino acid sensing (66, 67).

To then directly investigate BCR/TLR coactivation effects on lysosomal BCAA and BCKA, we performed targeted LC/MS analysis in whole cells or in lysosomes immunopurified from resting versus αIgM + CpG–stimulated Rael HA-LysoIP cells. Whole-cell Leu, Ile, and Val BCAA pools each significantly increased upon BCR/TLR9 costimulation. However, lysosomal Leu and Ile levels instead substantially decreased, suggesting that these amino acids were exported from lysosomes upon αIgM + CpG stimulation. Whole-cell BCKAs remained unchanged by BCR/TLR stimulation, whereas BCKAs were not detected in immunopurified lysosomes (Figure 5G). Collectively, our results support a model where BCAT1 augments Leu and Ile synthesis at the lysosomal membrane to support mTORC1 hyperactivation (Figure 5H).

### BCAT1 inhibition suppresses MCD DLBCL tumor growth in vitro and in vivo.

**Figure 6:**
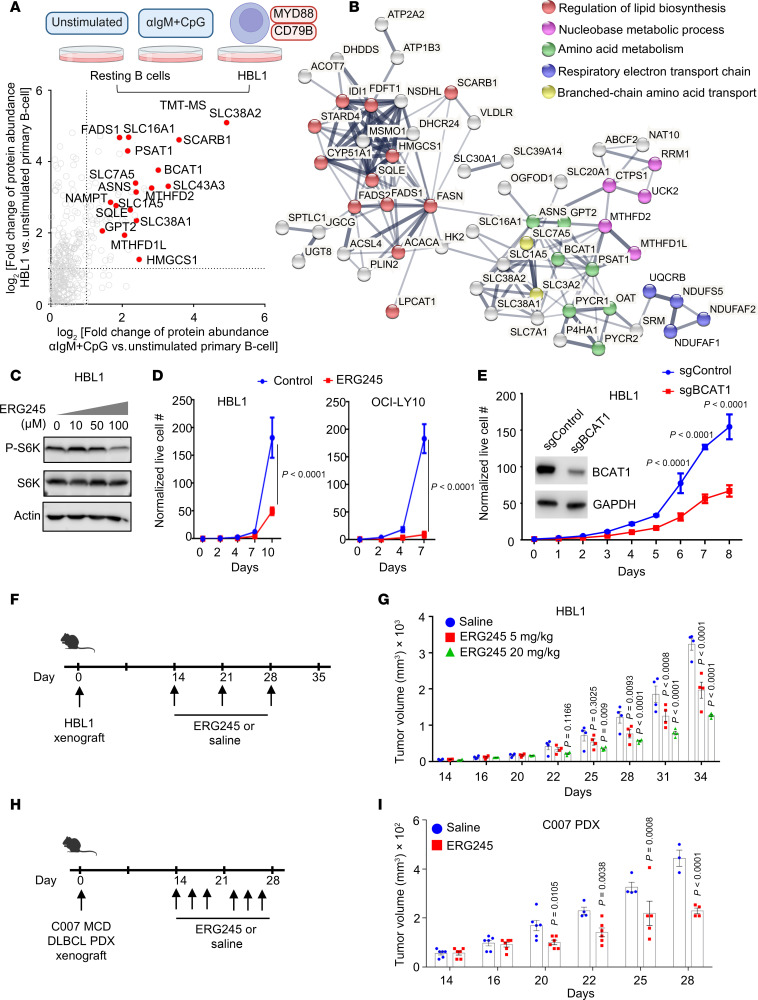
BCAT1 is highly expressed in BCR/TLR9-driven MCD DLBCL, where it supports B cell proliferation in vitro and in vivo. (**A**) Volcano plot comparing tandem-mass-tag proteomic log_2_ (fold change) of whole cell protein abundance in HBL1 DLBCL versus unstimulated primary B cell (*y* axis) versus 24 hours αIgM + CpG–stimulated versus unstimulated primary B cell (*x* axis). Proteins from a curated metabolic gene set are shown (34). From *n* = 3 proteomics dataset. (**B**) String analysis of protein-protein interactions among factors upregulated in both αIgM + CpG–stimulated and HBL1 (fold change >2) relative to unstimulated primary B cells. (**C**) Immunoblots of whole-cell lysates from HBL1 cells treated with vehicle, 10, 50, and 100 μM ERG245 as indicated for 24 hours. (**D**) Growth curve analysis of vehicle or 100 μM ERG245-treated HBL1 (left) or OCI-LY10 MCD DLBCL cells. Mean ± SD values from *n* = 3 replicates. (**E**) Growth curve analysis of Cas9 + HBL1 cells expressing control or BCAT1 sgRNAs. Mean ± SD values from *n* = 3 replicates. (**F**) Schematic of HBL1 MCD DLBCL mouse xenograft experiments. HBL1 tumors were implanted in mouse flanks 2 weeks prior to administration of vehicle versus 5 mg/kg or 20 mg/kg ERG245. (**G**) Mean ± SEM HBL1 tumor volumes in mice treated as indicated. (**H**) Schematic of C007 MCD DLBCL patient-derived xenograft (PDX) experiments. Tumors were implanted in mouse flanks 2 weeks prior to administration of vehicle versus 20 mg/kg ERG245. (**I**) Mean ± SEM C007 PDX tumor volumes in mice treated as indicated. *P* values were calculated by 2-way ANOVA followed by Šídák’s multiple-comparison test (**D**, **E**, and **G**) or Tukey’s multiple-comparison test (**I**).

mTORC1 is hyperactivated by the My-T-BCR complex in MCD DLBCL, where BCR, TLR9, and MyD88 form a super-complex that colocalizes with mTORC1 on endolysosomes (21). To gain insights into whether TLR9/BCR coactivation causes similar remodeling in primary B cells and in MCD DLBCL, we cross-compared proteomes from αIgM/CpG-stimulated primary B cells or the tumor-derived MCD DLBCL HBL1 cell line with resting primary B cells. Interestingly, a group of metabolic proteins were similarly upregulated in αIgM + CpG and in HBL1, presumably by My-T-BCR signaling in both contexts, including BCAT1 and SLC7A5. Using a fold-change of 2 or greater as the threshold, we identified that 1,280 proteins were commonly upregulated by αIgM + CpG and in HBL1, relative to their resting primary B cell levels (Figure 6A and Supplemental Table 7). STRING analysis (68) identified multiple metabolic subnetworks upregulated in both activated B cell contexts, including BCAA metabolism (Figure 6B).

To determine whether BCAT1 activity is likewise important for MCD DLBCL proliferation in vitro, we tested ERG245 effects on HBL1 and OCI-LY10. ERG245 reduced phospho-S6 levels, indicating its inhibitory effects on mTOR signaling (Figure 6C). ERG245 significantly reduced proliferation of both MCD cell lines (Figure 6D), which was further supported by the observation that CRISPR editing of BCAT1 also impaired HBL1 proliferation (Figure 6E). To extend this observation in vivo, we established HBL1 xenografts in NOD.Cg-Prkdcscid Il2rgtm1Wjl/SzJ (NSG) mice. HBL1 tumors were grown for approximately 14 days after implantation until tumor volumes reached 32–64 mm^3^. Mice were then treated weekly with either vehicle control or ERG245 at doses of 5 or 20 mg/kg via i.p. injection (Figure 6F). There was no significant difference in body weight observed between mice treated with vehicle control versus ERG245 at either the 5 or 20 mg/kg dose over the following 3 weeks (Supplemental Figure 14G), indicating that ERG245 was well tolerated. However, tumor volumes were significantly smaller from day 12 onward in ERG245-treated mice in a dose-dependent manner (Figure 6G). Tumor volumes of OCI-LY10 xenografts were also significantly reduced by ERG245 (Supplemental Figure 14H). To further extend these results, we next tested ERG245’s effects on an MCD DLBCL PDX model. Two weeks after establishment of the C007 PDX, ERG245 was dosed 3 times per week at 20 mg/kg (Figure 6H). ERG245 significantly decreased PDX tumor volumes beginning at day 7 after ERG245 dosing and significantly increased body weight of C007 PDX–carrying mice (Figure 6I and Supplemental Figure 14I).

## Discussion

B lymphocytes are uniquely positioned to integrate a wide range of antigenic, PAMP, and T cell cues (1, 2, 69–72). As a result, it is hypothesized that a signaling code drives distinct B cell responses to receptor stimuli (28). However, much remains to be learned about how key T cell–dependent versus T cell–independent B cell stimuli remodel immunometabolism networks to control B cell activity. Here, we present a multiomics compendium of acute primary human CD19^+^ peripheral blood B cell responses to BCR, TLR9, CD40, and/or IL-4R activation, each of which are prominent drivers of naive B cell responses. We found that BCR and TLR9 jointly induce PD-L1 as well as the transaminase BCAT1, which is targeted to lysosomes to support mTORC1 activation.

Immunometabolic regulation is critical for supporting B cell proliferation and effector functions (28, 73). Our data suggest that BCR/TLR9 induce BCAA production by BCAT1 at the lysosome membrane to support mTORC1 activation and B cell growth and survival. How BCAT1 homes to lysosomal membranes remains an intriguing question. Since this has not been observed in other cell types, an intriguing possibility is that a complex containing the BCR and TLR9 analogous to the My-T-BCR described in DLBCL may recruit BCAT1 to endolysosomes (21). Alternatively, BCR/TLR9 stimulation causes major lysosomal remodeling and may induce a protein or posttranslational modification to target BCAT1 to lysosomes. In support of this idea, highly spatially delineated roles are emerging as a theme in BCAT biology. For instance, a distinct spatially constrained mitotic spindle BCAT1-localized role was recently observed in epithelial cells (74), and BCAT2 also exerts a spatially regulated role, in which it forms a mitochondrial BCAA metabolon together with branched-chain α-keto acid dehydrogenase to shuttle BCAA catabolites into the TCA cycle (75).

Although BCAT1 catalyzes reversible transamination reactions, BCAA catabolism typically predominates (76, 77). We provide evidence that BCAT1 fluxes in both directions in BCR/TLR9-stimulated cells, but the majority of labeled leucine was [^13^C,^15^N]-leucine (m+7), suggesting that BCAA synthesis predominates. We speculate that the specific location of BCAT1 can determine its catabolic and anabolic activity. To test this hypothesis, subcellular mass spectrometry imaging (78) could be used to measure [^13^C,^15^N]-leucine in the lysosomal vicinity. Also suggestive of an anabolic role, BCR/TLR9 did not increase protein levels of the branched-chain α-keto acid dehydrogenase complex (BCKDC), which catalyzes the irreversible conversion of BCKA to acetyl- and succinyl-CoA for TCA metabolism. Notably, BCAT1 promotes BCAA production in BCR-ABL–driven chronic myelogenous leukemia, in which BCAT1 blockade impairs B cell proliferation and causes differentiation (77).

BCAT1 is expressed in CD4^+^ T cells, where it drives BCAA catabolism to instead downmodulate mTORC1 activity (79). BCAT1 also catabolizes BCAA in activated macrophages (39). Interestingly, CD8 T cell BCAT1 instead supports effector functions, although it does not influence BCAA levels (42). We speculate that differences in glutamine and BCKA levels may account for these differences. Notably, increased glutamate levels in EZH2-mutant acute myelogenous leukemia drive BCAA production by BCAT1 to support mTORC1 and to restrict αKG levels (80). It will therefore be of interest to determine whether BCAT1 homes to lysosomes in these other hematopoietic cell contexts.

Our results indicate that BCAT1 is not required for CD40L + IL-4–induced mTOR activation. Although BCAAs are well-established mTOR activators, other amino acids such as arginine can also activate mTOR (81, 82). CD40L + IL-4 stimulation may preferentially utilize alternative amino acid–dependent pathways for mTOR activation. Even under amino acid–depleted conditions, mTOR can remain active when AKT and ERK signaling are elevated (83, 84). CD40L + IL-4 may induce stronger activation of these pathways as compared with αIgM + CpG stimulation, thereby maintaining mTOR activity independently of BCAT1-mediated BCAA production.

Novel therapeutic targets are needed for the treatment of a wide range of pathological B cell states (71). BCAT1 may therefore constitute an intriguing metabolic vulnerability, including in MCD DLBCL and in certain autoimmunity states, including systemic lupus erythematosus, where BCR/TLR7 drives pathology. Since BCAT1 inhibition also increases CD4^+^ T cell mTORC1 activation and ameliorates CD8^+^ T cell exhaustion, BCAT1 antagonists may be particularly promising for DLBCL. BCAT1 antagonists may also exert synergy with glutaminase to further reduce BCAA levels and mTORC1. BCAT1 may also serve as a biomarker for pathological BCR/TLR9-driven B cell states.

We acknowledge several limitations of the above studies. First, most of our studies were performed in vitro so that we could characterize the initial responses of peripheral blood primary human B cells to a range of defined receptor stimuli over a key time point of B cell activation and differentiation. Although extensive studies have been performed on murine B cells, we chose this approach to address the relative gap in studies of primary human B cell responses. Although performed ex vivo, nearly all studies were done within the first 24 hours of harvest, a time point prior to the onset of proliferation and designed to minimize the effects of tissue culture. However, key findings were validated in vivo using murine models. Future studies could utilize humanized mice to validate and extend aspects of these studies in vivo, although harvesting sufficient numbers of B cells would require significant cell handling after stimulation such as FACS from explanted spleen, which could perturb key metabolic, transcriptomic, and proteomic responses. Second, although effects of BCAT1 inhibition were studied on primary human B cells in vitro and in transformed human B cells in vivo, BCAT1 roles in primary human cells remain to examined in vivo. Given concurrent BCAT1 roles in T cell and macrophage activation, B cell–specific BCAT1-KO murine models could be used to characterize B cell–intrinsic BCAT1 roles in vivo. However, murine B cell responses may differ from those in humans. Third, our studies focused on purified B lymphocyte responses and therefore do not capture additional effects of a more complex immune milieu, such as occurs in secondary lymphoid organs. Future studies could harness human tonsil organoids, which provide a controlled yet rich cellular milieu for studies of B cell responses (85), though secondary effects of TLR9, CD40, and IL-4 stimulation on additional immune cells such as monocyte/macrophages could complicate such analyses. Fourth, most of our studies characterized bulk B cell responses to receptor stimuli rather than responses at the single-cell level. Future studies could harness single-cell RNA-Seq to characterize distinct primary human B cell subpopulation responses to stimuli. Fifth, we performed lysosomal immunopurification assays in transformed rather than primary B cells because we were unable to achieve sufficient TMEM192 transgene expression in freshly isolated human B cells within 24 hours of harvest. Finally, we used IL-4 in combination with CD40L, and it will be of interest to test effects of other B cell–activating cytokines such as IL-21 or IFN-γ, which also activate JAK/STAT pathways and which may therefore have considerable overlap in phenotypes with IL-4 (86).

In summary, we used multiomics profiling to systematically characterize primary human B cell responses to key receptor stimuli. Collectively, our studies provide a major resource for primary human B cell immunometabolism investigation. We identified major immunometabolism pathways that differ on the transcriptional, proteomic, and metabolomic levels with receptor-driven metabolism reprogramming. BCR/TLR9 but not CD40/IL-4 costimulation highly induced BCAT1, which trafficked to lysosomal membranes to support BCAA synthesis and mTORC1 hyperactivation. BCAT1 was critical for BCR/TLR9– but not CD40/IL-4–driven primary B cell growth and survival. BCAT1 inhibition significantly impaired growth of BCR/TLR9 pathway–dependent MDC DLBCL xenografts in vivo, identifying BCAT1 as a promising B cell lymphoma therapeutic target.

## Methods

### Sex as a biological variable.

Primary human B cells used in this study were isolated from deidentified, discarded leukocyte fractions and may be derived from both male and female donors. Our study examined male and female animals, and similar findings are reported for both sexes.

### Cell lines, reagents, and antibodies.

All cell lines, reagents, and antibodies used in this study are detailed in the Supplemental Methods.

### Primary human B cells.

Discarded, deidentified leukocyte fractions left over from platelet donations were obtained from the Brigham and Women’s Hospital Blood Bank or from the Gulf Coast Medical Center after collection of informed consent. Blood cells were collected from platelet donors following institutional guidelines. Since these were deidentified samples, the gender was unknown. Our studies on primary human blood cells were approved by the Brigham & Women’s Hospital IRB. B cells from Gulf Coast Medical Center were used for RNA-Seq analyses. Primary human B cells were isolated by negative selection using RosetteSep human B cell enrichment and EasySep human B cell enrichment kits (Stem Cell Technologies), according to the manufacturers’ protocols. B cell purity was confirmed by FACS analysis of plasma membrane CD19 positivity. Cells were then cultured with RPMI 1640 with 10% FCS; 7 × 10^6^ B cells were used for each stimulation condition. Stimulants were added at the following concentrations: CD40L, 50 ng/mL (Enzo Life Sciences); CpG, 0.5 μM (IDT); IL-4, 20 ng/mL (R&D Systems); and αIgM, 1 μg/mL (Sigma-Aldrich.). Cells were cultured in the absence of stimulation or stimulated by CD40L only, CpG only, IL-4 only, αIgM only, CD40L + CpG, CD40L + IL-4, CpG + αIgM, CD40L + αIgM, and CD40L + αIgM + IL-4. Cells were treated for 24 hours. For each experiment, cells from 3 donors were isolated and treated separately. At 24 hours, cells were counted and viability was measured using trypan blue staining and counted on a TC20 automated cell counter (Bio-Rad).

### Mice.

For in vitro and in vivo B cell stimulation experiments, C57BL/6-Tg(IghelMD4)4Ccg/J (MD4, 002595) transgenic mouse strain (87) and C57BL/6J (stock 000664) were procured from The Jackson Laboratory. MD4 mice were maintained as hemizygous strains with C57BL/6J mice because homozygous MD4 strains breed poorly. All mice were bred and housed in a specific pathogen–free environment at the Karp Research Facility of Boston Children’s Hospital. All mice used in this study were 6–8 weeks old and animal experiments were approved by the IACUC of Boston Children’s Hospital (approval 00001696).

For mouse xenograft experiments, NOD.Cg-Prkdcscid Il2rgtm1Wjl/SzJ (NSG) immunocompromised mice were procured from The Jackson Laboratory (stock number 005557) and maintained in Weill Cornell Medical Center (WCMC) in accordance with the IACUC of WCMC (2017-0035).

### Statistics.

Unless otherwise indicated, all bar graphs and line graphs represent the arithmetic mean of 3 independent experiments, with error bars denoting standard deviations. Data were analyzed using 2-tailed paired Student’s *t* test or 1- or 2-way ANOVA with the appropriate post test using GraphPad Prism7 software. GO analysis was done with the Enrichr module using the Kyoto Encyclopedia of Genes and Genomes (KEGG) pathway databases. Default parameters of the Enrichr module were used, with the exception that the enrichment statistic was set as classic. Metabolic pathway analyses were performed using MetaboAnalyst 3.0. Figures were drawn with commercially available GraphPad, BioRender, and Microsoft PowerPoint software. A *P* value less than 0.05 was considered statistically significant.

### Study approval.

Research involving primary human blood cells was conducted with approval from the Brigham & Women’s Hospital IRB (protocol 2022p001270). All study participants provided written informed consent. In vitro and in vivo stimulation experiments using mouse B cells received ethical approval from the IACUC at Boston Children’s Hospital (protocol 00001696). All mouse xenograft procedures were performed in compliance with the IACUC guidelines at WCMC (protocol 2017-0035).

### Data availability.

All RNA-Seq datasets have been deposited in NCBI’s Gene Expression Omnibus (GEO). The accession number for the RNA-Seq dataset reported in this paper is GSE232769. The mass spectrometry proteomics data have been deposited to the ProteomeXchange Consortium via the PRIDE partner repository with the dataset identifier PXD016961. Original data values are available in the Supporting Data Values file. All plasmids and cell lines generated in this study will be made available on request.

### Code availability.

No custom code was used for this study.

## Author contributions

Conceptualization was carried out by RG and BEG. Methodology was developed by RG, YS, RAA, DRW, MRG, SPG, BEG, VKM, HS, AM, JAP, and JMA. Investigation was performed by MYL, MMLL, YZ, LWW, DS, NAS, M Lutchenkov, JHL, ZL, YW, RP, RAA, DRW, YS, and RG. M Li contributed through helpful discussions. HY, WL, and AEP provided key reagents. Visualization was provided by RG and YS. Supervision was undertaken by RG, BEG, and VKM. The original draft was written by RG, YS, and BEG. Review and editing of the manuscript were performed by all authors. RG and YS contributed equally to this work.

## Funding support

This work is the result of NIH funding, in whole or in part, and is subject to the NIH Public Access Policy. Through acceptance of this federal funding, the NIH has been given a right to make the work publicly available in PubMed Central.

- NIH grants R01 DE033907, AI137337, and U01CA275301 (to BEG); R00 DE031016, to RG; GM67945, to SPG; and GM132129, to JAP.
- Burroughs Wellcome Career Award in Medical Sciences, to BEG.
- Lymphoma Research Foundation Fellowship, to RG.
- American Cancer Society Postdoctoral Fellowship, PF-23-1144614-01-IBCD, to YS.
- Howard Hughes Medical Institute, to VKM.
- National Science Scholarship, Singapore, to LWW.
- Agency for Science, Technology and Research (A*STAR) Biomedical Research Council Central Research Fund for Use-Inspired Research, Singapore, to LWW.
- Swiss National Science Foundation, P400PB_199261 and P2ELP3_187926, to DS.

## Supplementary Material

Supplemental data

Unedited blot and gel images

Supplemental table 1

Supplemental table 2

Supplemental table 3

Supplemental table 4

Supplemental table 5

Supplemental table 6

Supplemental table 7

Supporting data values

## Version 1 09/09/2025

In-Press Preview

## Version 2 11/17/2025

Electronic publication
