# Supplementary material for: Multiomic analysis reveals a key BCAT1 role in mTOR activation by B cell receptor and TLR9
Source: J Clin Invest. 2025 Sep 9;135(22):e186258. doi: 10.1172/JCI186258 (PMC12618069; doi:10.1172/JCI186258)
Supplement: Supplemental data [file jci-135-186258-s037.pdf]

Supplemental materials

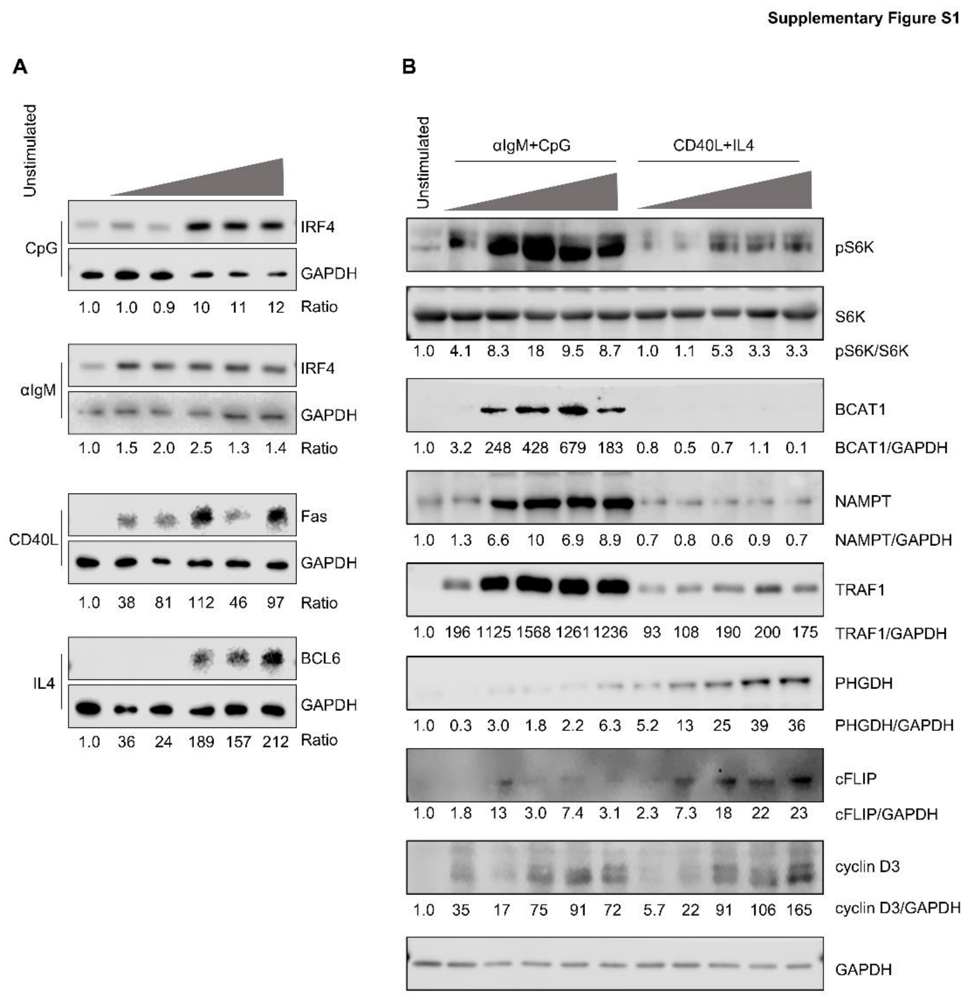

Supplementary Figure S1. Analysis of B-cell stimulus specific dose-response, related to Figure 1.

(A) Immunoblot of whole cell lysates (WCL) from peripheral blood B cells stimulated by CpG, αIgM, CD40L or IL4 for 24h at the following concentrations: CpG 0.125, 0.25, 0.5, 1, 2 μM; αIgM 0.25, 0.5, 1, 2, 4 μg/ml; CD40L 12.5, 25, 50, 100, 200 ng/ml; IL4 5, 10, 20, 40 ,80 ng/ml. GAPDH- or S6K-normalized densitometry ratios are shown below each lane and were also normalized to unstimulated control ratios, which were set to 1.

(B) Immunoblot of WCL from peripheral blood B cells stimulated with αIgM+CpG or CD40L+IL4 for 24h at the following concentrations: CD40L and IL4 12.5/5, 25/10, 50/20, 100/40, and 200/80 ng/ml; CpG and αIgM at 0.125/0.25, 0.25/0.5, 0.5/1, 1/2, and 2/4 μM/ml or μg/ml, respectively.

GAPDH- or S6K-normalized densitometry ratios are shown below each lane and were also normalized to unstimulated control ratios, which were set to 1.

Supplementary Figure S2

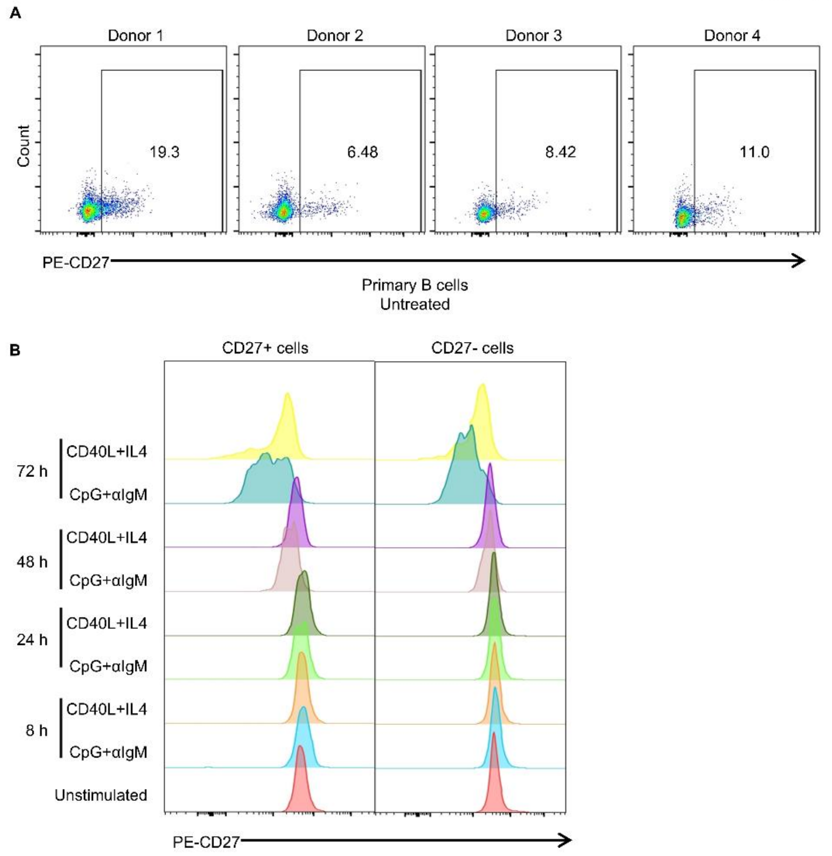

Supplementary Figure S2. Proliferative responses of peripheral blood CD27+ vs CD27- B cells to αIgM+CpG versus CD40+IL4 stimulation, related to Figure 1.

(A) FACS analysis of human peripheral blood B cells subsets. CD27 negativity was used as a naïve B cell marker, whereas CD27+ was used to mark circulating memory B cells. CD27+ cell percentages are indicated from each of four independent donors.

(B) FACS analysis of CFSE-stained peripheral blood B-cells treated with αIgM(1μg/ml)+CpG (0.5μM) or CD40L(50ng/ml)+IL4(20ng/ml), as indicated. CFSE levels are diluted by 50% with each mitosis. CD27- and CD27+ cells were gated for the analysis. Proliferation was not evident until at least 48 hours post-infection. Representative of n = 3 replicates.

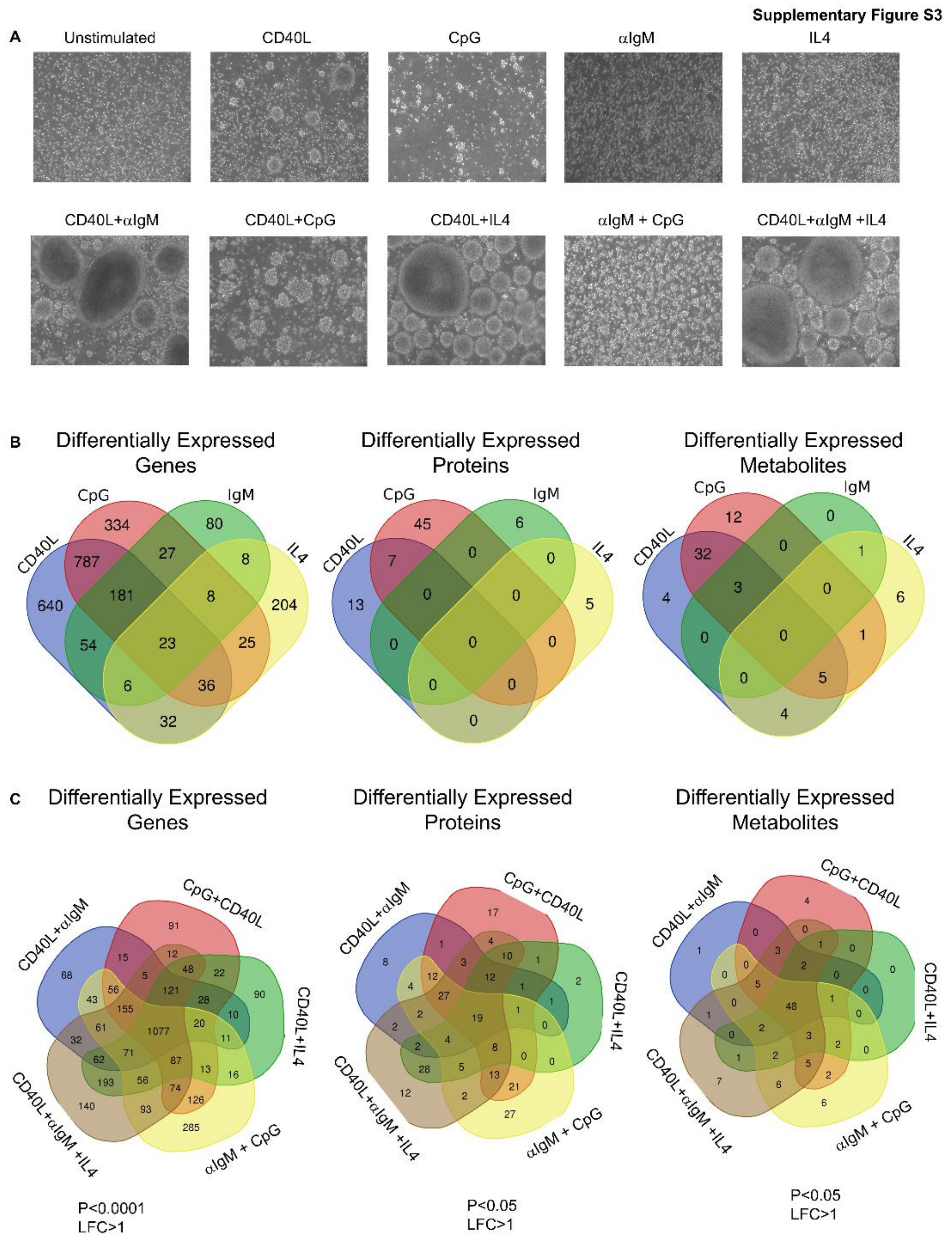

Supplementary Figure S3. Receptor-driven B-cell activation results in different cellular responses, related to Figure 1.

(A) Representative phase microscopic analyses of CD19+ human peripheral blood B-cells stimulated as indicated for 96 hours. Scale bar, 100μm.

(B) Venn diagram analysis of differentially expressed genes, proteins and metabolites across single receptor stimulation conditions.

(C) Venn diagram analysis of differentially expressed genes, proteins and metabolites across combinatorial receptor stimulation conditions.

Supplementary Figure S4

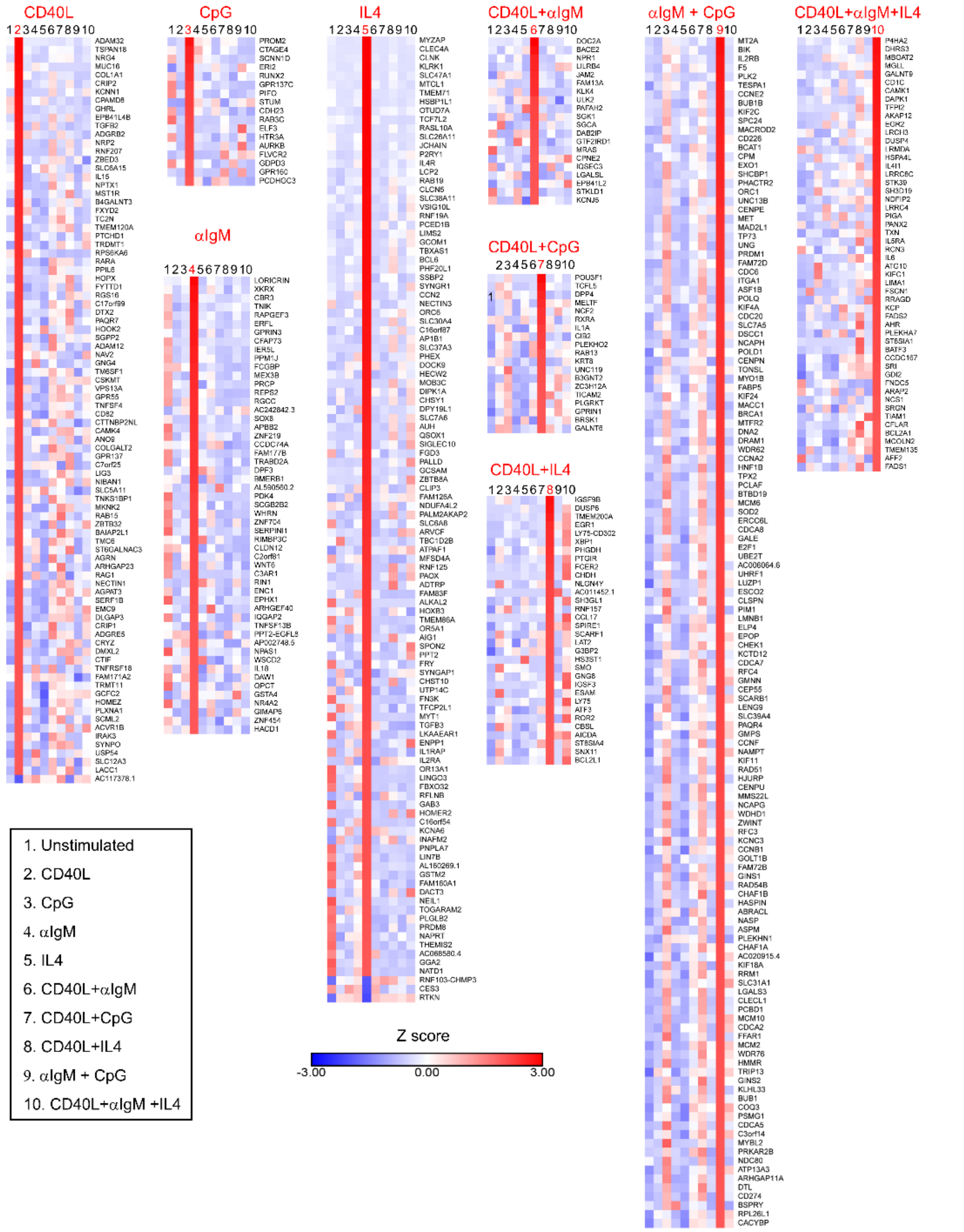

Supplementary Figure S4. Heatmap visualization of the genes most preferentially induced by each condition, related to Figure 1.

Shown are row Z-scores for the indicated genes across the 10 stimulation conditions indicated at bottom left.

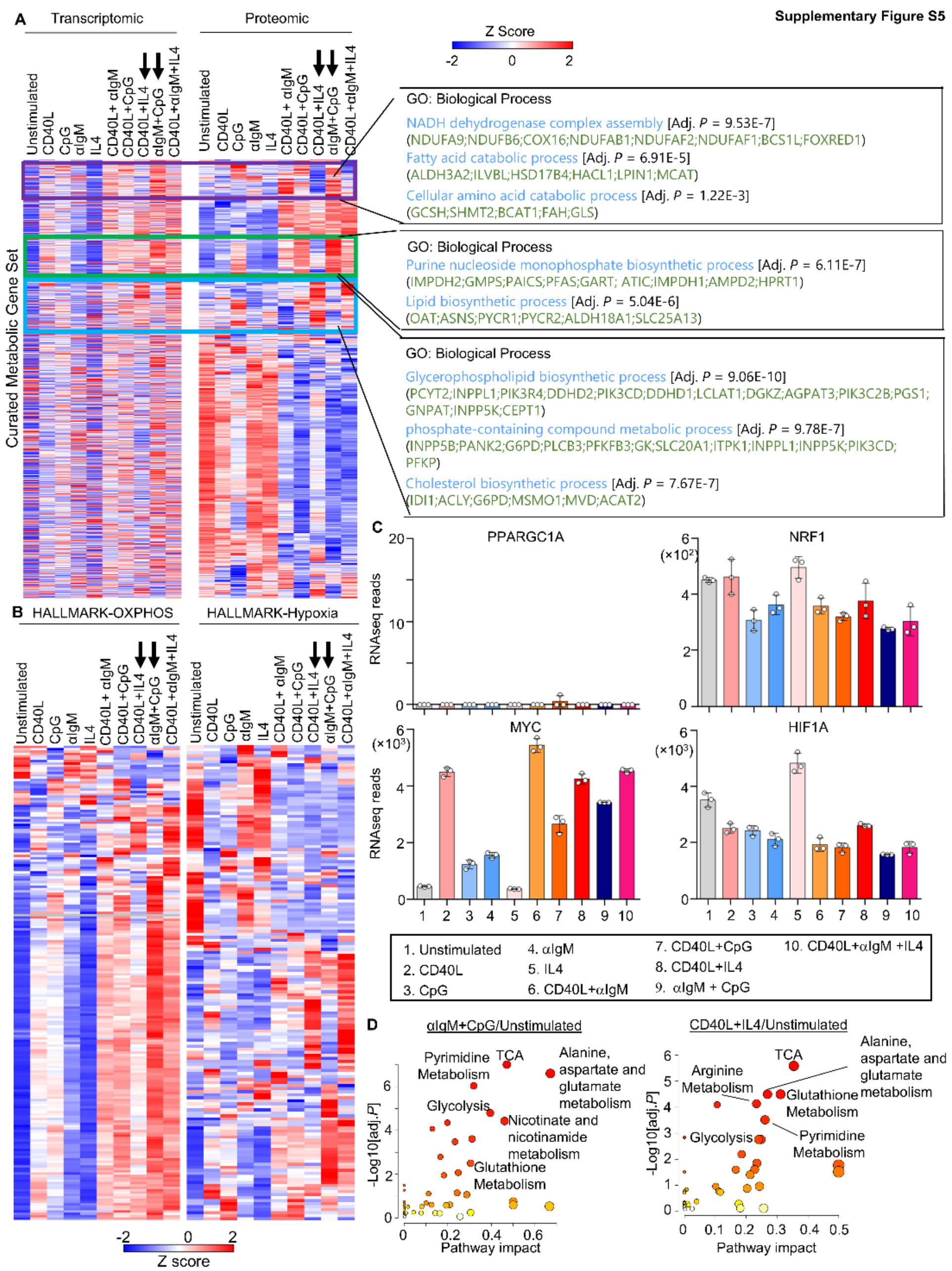

Supplementary Figure S5. B-cell stimulus specific metabolic pathway responses, related to Figure 2.

(A) Heatmap analysis showing metabolic gene mRNA or protein Z-scores from primary human B-cells stimulated for 24h, as indicated, using a curated metabolism gene set. Hierarchical clustering was performed independently for both datasets and then genes were aligned. GO Biological Process analysis of three clusters is highlighted at right.

(B) Heatmap analysis showing Z-scores of mRNAs encoding metabolism genes in primary human B-cells stimulated for 24h, as indicated.

(C) mRNA abundances of the indicated genes in primary B-cells stimulated as indicated for 24h.

(D) MetaboAnalyst Pathway enrichment analysis of metabolites differentially upregulated in primary B-cells stimulated by αIgM+CpG (left) or CD40L+IL4 (right) for 24h vs unstimulated cells.

Supplementary Figure S6

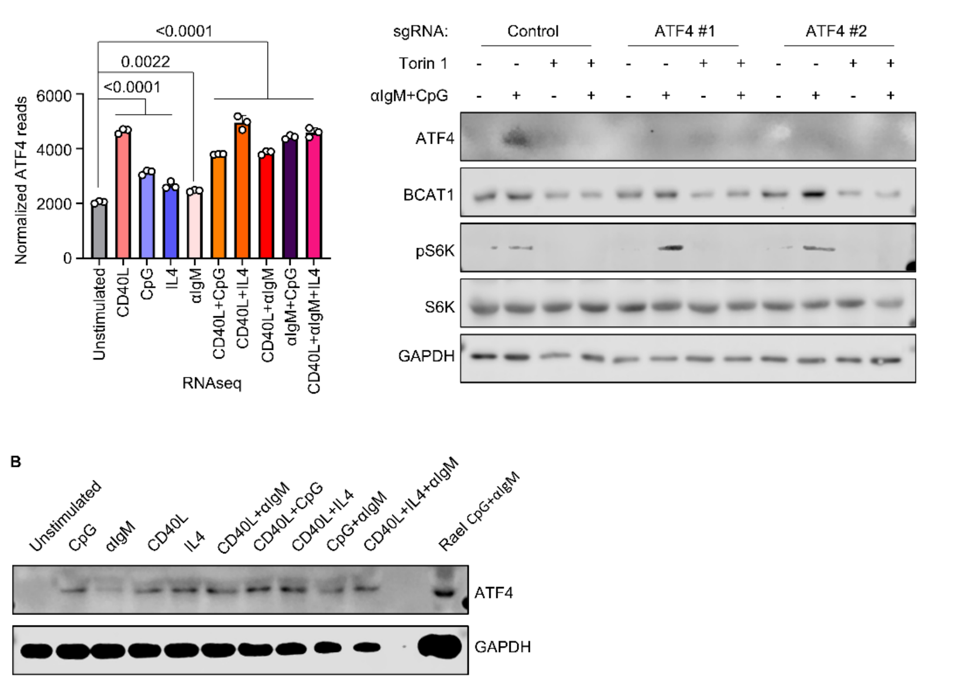

Supplementary Figure S6. Multiple B-cell agonists induce ATF4 expression, related to Figure 1-2.

(A) Mean + SD of normalized ATF4 reads from n=3 RNAseq replicates presented in Fig. 1 of human peripheral blood B-cells stimulated for 24h, as indicated. P-values were calculated by one-way ANOVA followed by Tukey's multiple comparisons test.

(B) Immunoblot analysis of WCL from human peripheral blood B cells stimulated for 24 hours, as indicated.

(C) Immunoblots of WCL from Cas9+ Rael B-cells that expressed control or independent ATF4 targeting sgRNAs and that were stimulated by αIgM+CpG for 24 hours, in the absence or presence of mTORC1/2 inhibitor Torin 1, as indicated.

Blots in B and C are representative of three independent replicates.

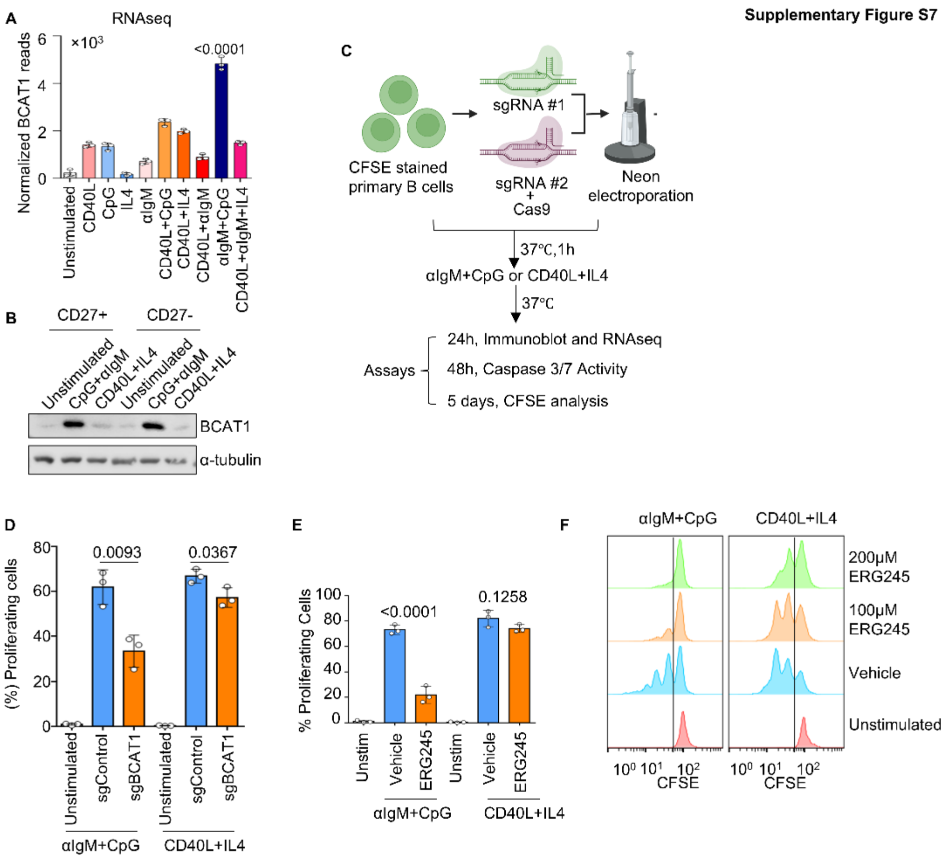

Supplementary Figure S7. BCR/TLR9 co-stimulation highly induces BCAT1, which is essential for aIgM/CpG but not CD40/IL4-driven primary-B-cell mTORC1 activation, growth and survival, related to Figure 3.

(A) Mean ± SEM of normalized BCAT1 reads from n=3 RNAseq datasets from B-cells stimulated as indicated for 24h. P values were calculated by multiple t tests using Holm-Sidak method.

(B) Immunoblots of CD27+ and CD27- B cells stimulated as indicated for 24h. Primary B cells were stained with PE-CD27 antibody, and isolated by magnetic bead selection of PE+ cells.

(C) Experimental design for experiments in Figure 3E, G, I and K. CFSE pre-stained human primary-B-cells were electroporated with Cas9 RNPs loaded with control or BCAT1 sgRNAs. To increase BCAT1 KO efficiency, two BCAT1 sgRNA/Cas9 RNPs were electroporated simultaneously. Cells were then rested for 1 hour and subjected to αIgM+CpG or CD40L+IL4 stimulation. Unstimulated cells were used as a control. Cells were collected at indicated time points for different assays.

(D) Mean ± SEM of percentages of proliferating cells as in Figure 3E, from n=3 replicates. P values were calculated by two-tailed paired Student t test.

(E) Mean ± SEM of percentages of proliferating cells as in Figure 3F, from n=3 replicates. P values were calculated by two-tailed paired Student t test.

(F) FACS analysis of CFSE-stained primary-B-cells treated with H_2O, 100μM ERG245, or 200μM ERG245 from 1 hour prior to αIgM+CpG or CD40L+IL4 stimulation, as indicated. Representative of n = 3 replicates.

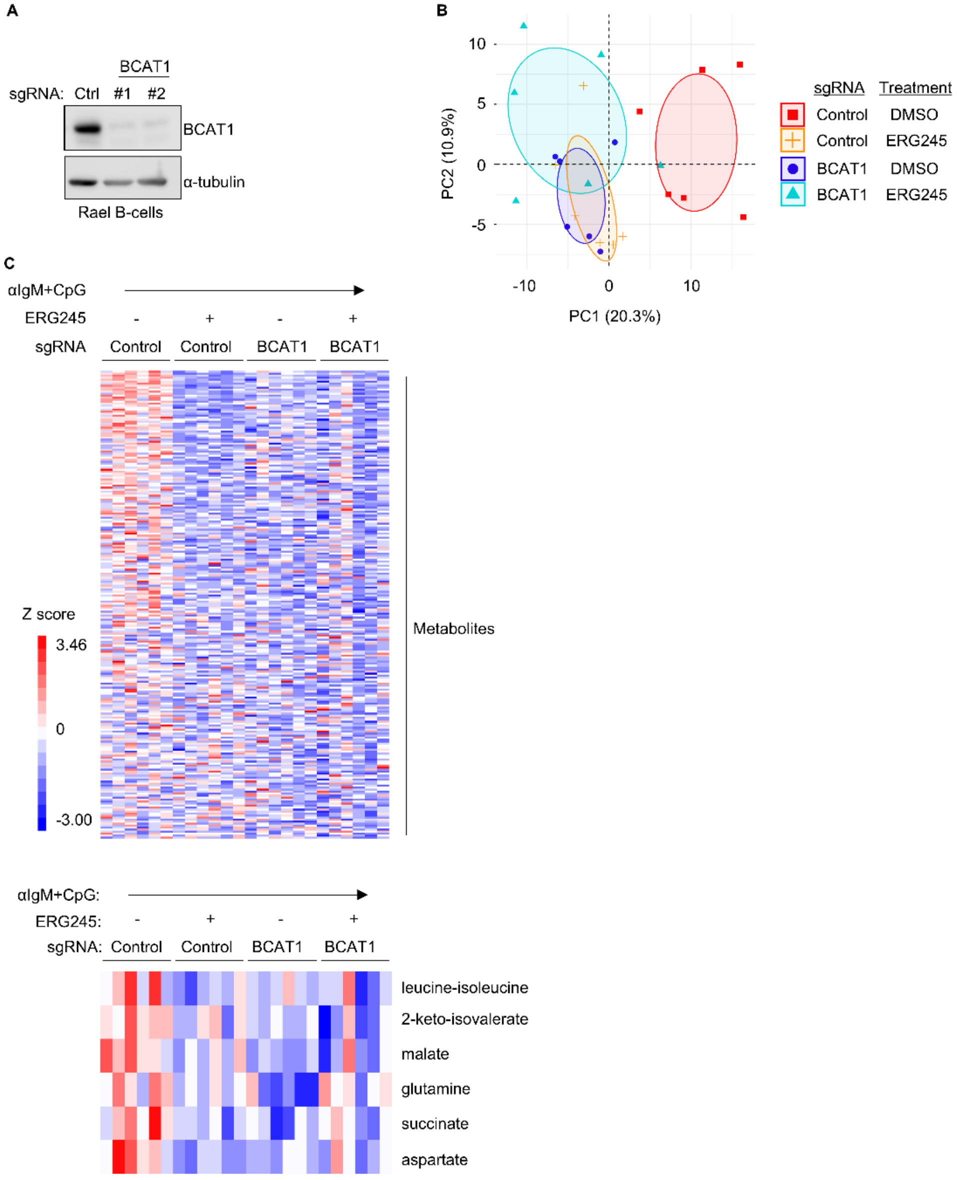

Supplementary Figure S8. Validation of ERG245 on-target effects, related to Figure 3.

(A) Immunoblots of WCL from Cas9+ Rael B-cells expressing the indicated control or independent BCAT1 targeting sgRNAs.

(B) Principle component (PC) analysis of intracellular metabolite analyses of Cas9+ Rael B-cells expressing control or BCAT1 targeting sgRNAs, treated with DMSO vehicle or ERG245 (100µM) and stimulated by αIgM+CpG for 24h, from n=6 replicates. Metabolite expression levels were normalized to cell counts for the analysis. PC analysis highlights similarities between BCAT1 knockout and ERG245-treated cells.

(C) Heatmap analysis of metabolite Z-scores from Rael cells as in (B), from n=6 replicates. Shown below are zoomed in values for selected metabolites.

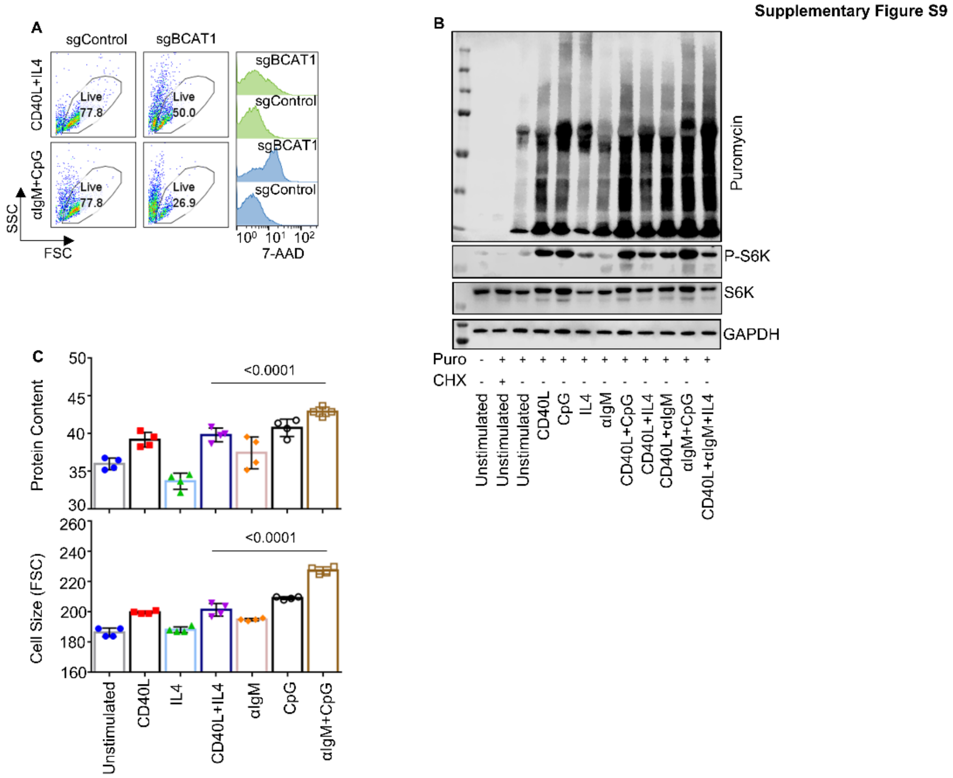

Supplementary Figure S9. BCR/TLR9 co-stimulation strongly activates mTORC1, related to Figure 3.

(A) FACS forward scatter (FSC) and side scatter (SSC) analysis of live cell numbers of primary B-cells electroporated with Cas9 RNPs loaded with Control (left panel) or BCAT1 (middle panel) sgRNAs and stimulated as indicated. 7-AAD vital dye uptake analysis us shown at right.

(B) Immunoblots of puromycin incorporation into newly synthesized polypeptides (top) or of the indicated proteins from WCL from primary B-cells stimulated by the indicated conditions for 24 hours. Puromycin pulse was performed by adding 10μg/ml puromycin to cells for 20min at 37℃. 100μg/ml cycloheximide was used as a negative control.

(C) FACS analysis of total protein content (top) and forward side scatter (FSC) cell size (bottom) in primary B-cells stimulated for 24h as indicated and labeled with eBioscience efluor 670 (5μM). MFI ± SEM from biological quadruplicate replicates are shown. P-values were calculated by one-way ANOVA followed by Dunnett's multiple comparisons test.

Supplementary Figure S10

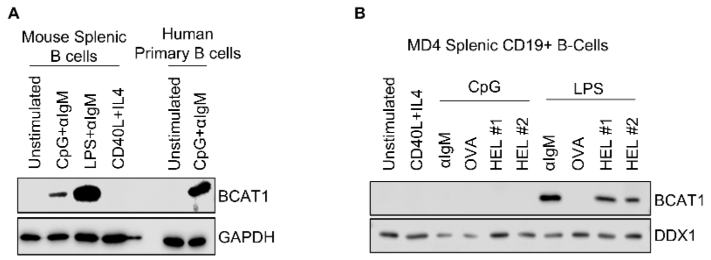

Supplementary Figure S10. BCR/TLR co-stimulation highly induces BCAT1 in murine B-cells *ex vivo*, related to Figure 3.

(A) Analysis of BCAT1 induction in murine splenic B-cells. Immunoblot of WCL from C57BL/6J murine splenic CD19+ B cells mock-stimulated or stimulated *in vitro* for 48h by 0.5μM CpG or 10 μg/ml LPS together with 1μg/ml αIgM, or by 50ng/ml CD40L + 20ng/ml IL4. For cross-comparison, WCL from human peripheral blood CD19+ B-cells, mock stimulated or stimulated by αIgM+CpG, were included at right.

(B) Immunoblot of WCL from MD4 murine splenic CD19+ B cells stimulated *ex vivo* for 48h as indicated by 50ng/ml CD40L + 20ng/ml IL4, 0.5μM CpG or 10 μg/ml LPS together with either 1μg/ml αIgM, negative control ovalbumin (OVA, 1 μg/ml) or by hen egg lysozyme (HEL, 1 μg/ml). Splenic B-cells form two independent mice (termed HEL#1 and #2) were tested.

Blots are representative of n=2 replicates.

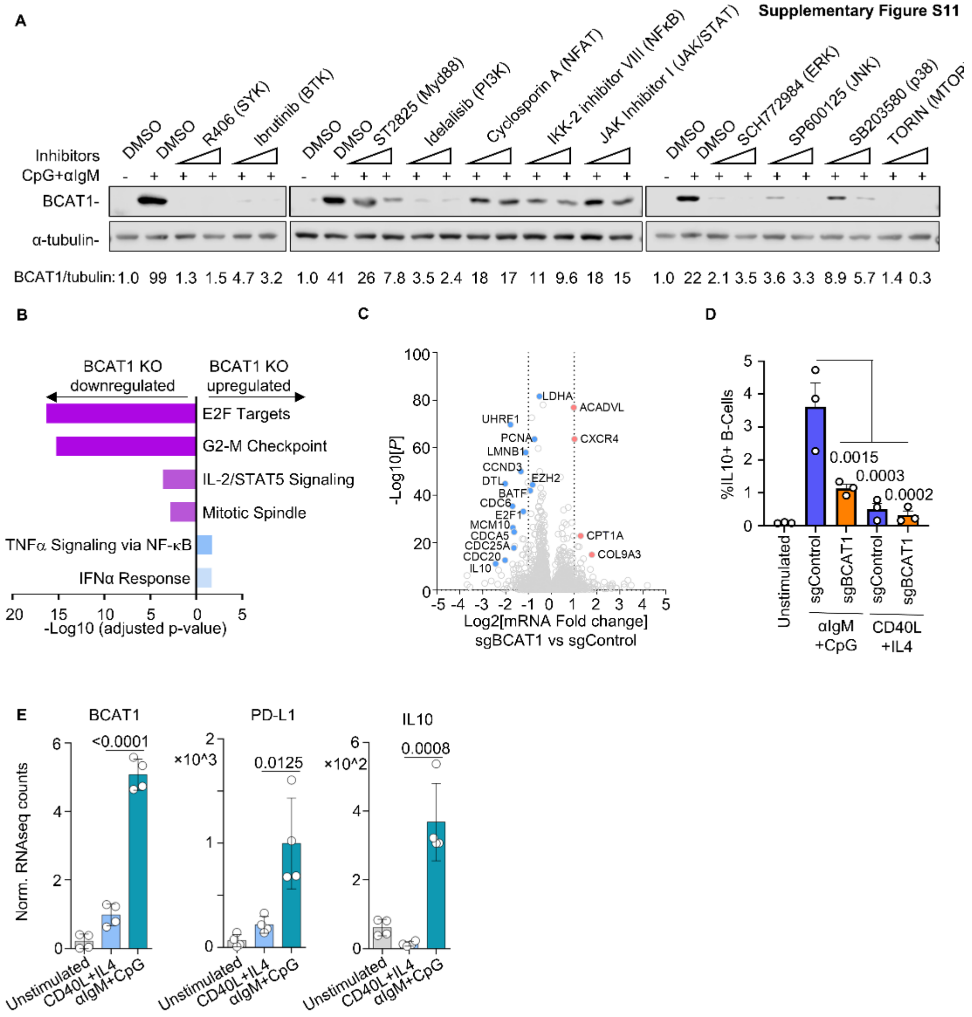

Supplementary Figure S11. Identification of mediators of BCAT1 induction and of BCAT1 regulated genes, related to Figure 3.

(A) Immunoblot of WCL from human peripheral blood B cells stimulated with αIgM+CpG and treated with indicated vehicle or small molecule inhibitors for 24h. Inhibitors were used at published low versus high concentrations as detailed in the method section. Shown at bottom are the fold change BCAT1 abundances relative to α-tubulin load control. Values in unstimulated, DMSO-treated cell WCL were set to 1 in each immunoblot panel, which were run in parallel at the same time. Blots were representative of n=2 replicates.

B) GSEA Hallmark pathway analysis of genes differentially expressed in primary cells electroporated with Cas9 control or BCAT1 RNPs and αIgM+CpG stimulated for 24h.

(C) Volcano plot visualization of -Log10 (p-value statistical significance) vs Log2 (fold-change mRNA abundance) from RNAseq of primary B-cells electroporated with Cas9 RNPs loaded with control or BCAT1 sgRNAs and stimulated by αIgM+CpG for 24h, from n=3 datasets.

(D) FACS analysis of the percentage of IL10+ B-cell from biological quadruplicate replicates of human peripheral blood CD19+ B-cells that were electroporated with Cas9 RNPs loaded with

control versus BCAT1 targeting sgRNAs and stimulated by αIgM+CpG vs CD40L+IL4 for 24h, as indicated. IL10 secretion was blocked by treatment with a protein transport inhibitor cocktail for 5 hours prior to FACS analysis. P-values were calculated by one-way ANOVA followed by Tukey's multiple comparisons test.

(E) Normalized RNAseq counts of BCAT1, PD-L1 and IL-10 mRNAs in human primary B cells unstimulated or stimulated by αIgM+CpG or CD40L+IL4 for 48 hours. P-values were calculated by one-way ANOVA followed by Dunnett's multiple comparisons test.

Supplementary Figure S12

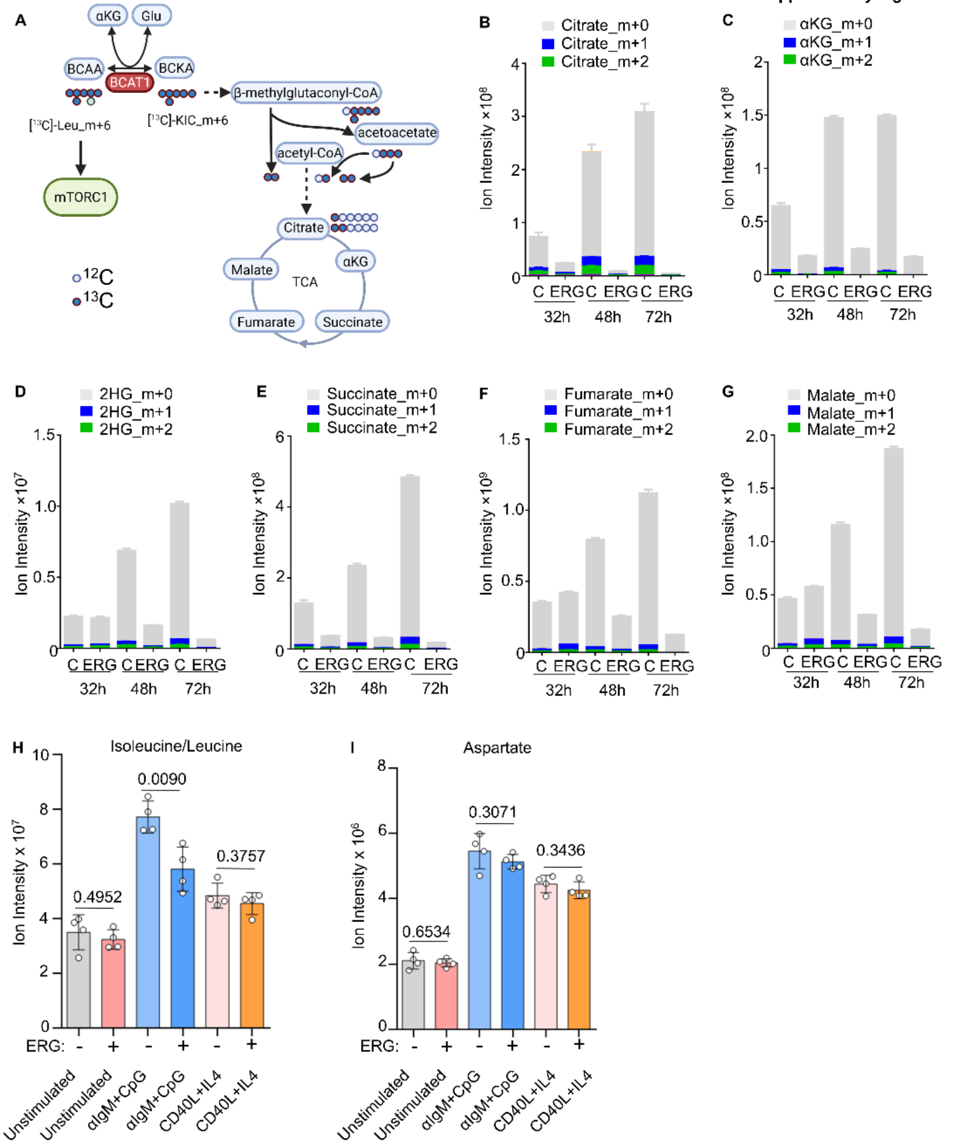

Supplementary Figure S12. BCR/TLR9 Stimulation drives BCAA synthesis in human primary B-cells, related to Figure 4.

(A) Schematic of BCAA metabolism and TCA cycle tracing. Catabolism of Leu and Ile generates acetyl-CoA, which replenishes the TCA cycle. [13C]-L-Leucine_m+6 was used to trace incorporation of carbons from leucine into TCA cycle metabolites.

(B) LC/MS ion intensities of citrate isotopomers from primary B-cells stimulated by αIgM/CpG for 24hrs in the presence of H_2O control (C) or 100μM ERG245.

(C) LC/MS ion intensities of α-ketoglutarate isotopomers from primary B-cells stimulated by αIgM/CpG for 24hrs in the presence of H_2O control (C) or 100μM ERG245.

(D) LC/MS ion intensities of 2-hydroxyglutarate isotopomers from primary B-cells stimulated by αIgM/CpG for 24hrs in the presence of H_2O control (C) or 100μM ERG245.

(E) LC/MS ion intensities of succinate isotopomers from primary B-cells stimulated by αIgM/CpG for 24hrs in the presence of H_2O control (C) or 100μM ERG245.

(F) LC/MS ion intensities of fumarate isotopomers from primary B-cells stimulated by αIgM/CpG for 24hrs in the presence of H_2O control (C) or 100μM ERG245.

(G) LC/MS ion intensities of malate isotopomers from primary B-cells stimulated by αIgM/CpG for 24hrs in the presence of H_2O control (C) or 100μM ERG245.

(H) Mean ± SEM intracellular leucine and isoleucine abundances from the Figure 4G metabolomic analysis of primary B-cells stimulated as indicated in the presence of H_2O versus 100 μM ERG245.

(I) Mean ± SEM intracellular aspartate abundances from the Figure 4G metabolomic analysis of primary B-cells stimulated as indicated in the presence of H_2O versus 100μM ERG245.

P-values were calculated by Two-way ANOVA followed by Šídák's multiple comparisons test (H and I).

Supplementary Figure S13

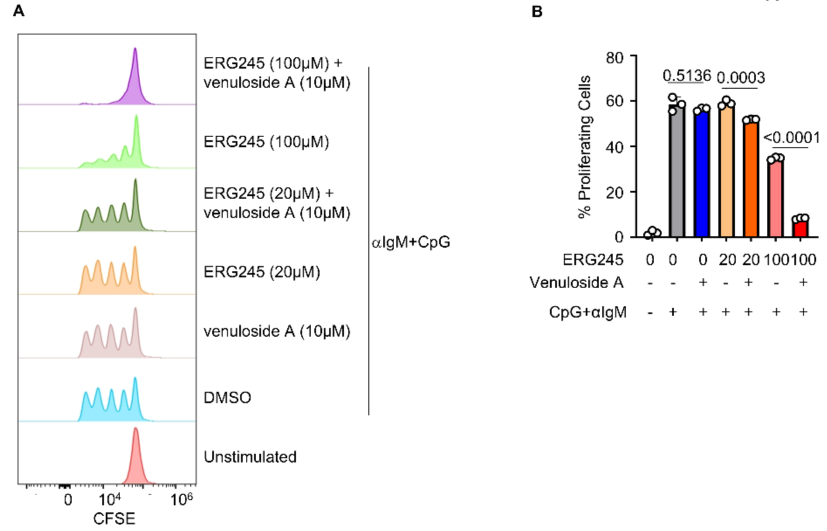

Supplementary Figure S13. Proliferative effects of LAT3 on αIgM+CpG stimulated cells, related to Figure 4.

(A) FACS analysis of CFSE-stained primary-B-cells treated with αIgM+CpG and BCAT1 inhibitor ERG245 or LAT3 inhibitor venuloside A for 5 days, as indicated. αIgM, CpG, ERG245 and venuloside A were replenished after 3 days.

(B) Mean + SEM of percentages of proliferating cells as in (A) from n=3 replicates. P-values were calculated by one-way ANOVA followed by Tukey's multiple comparisons test.

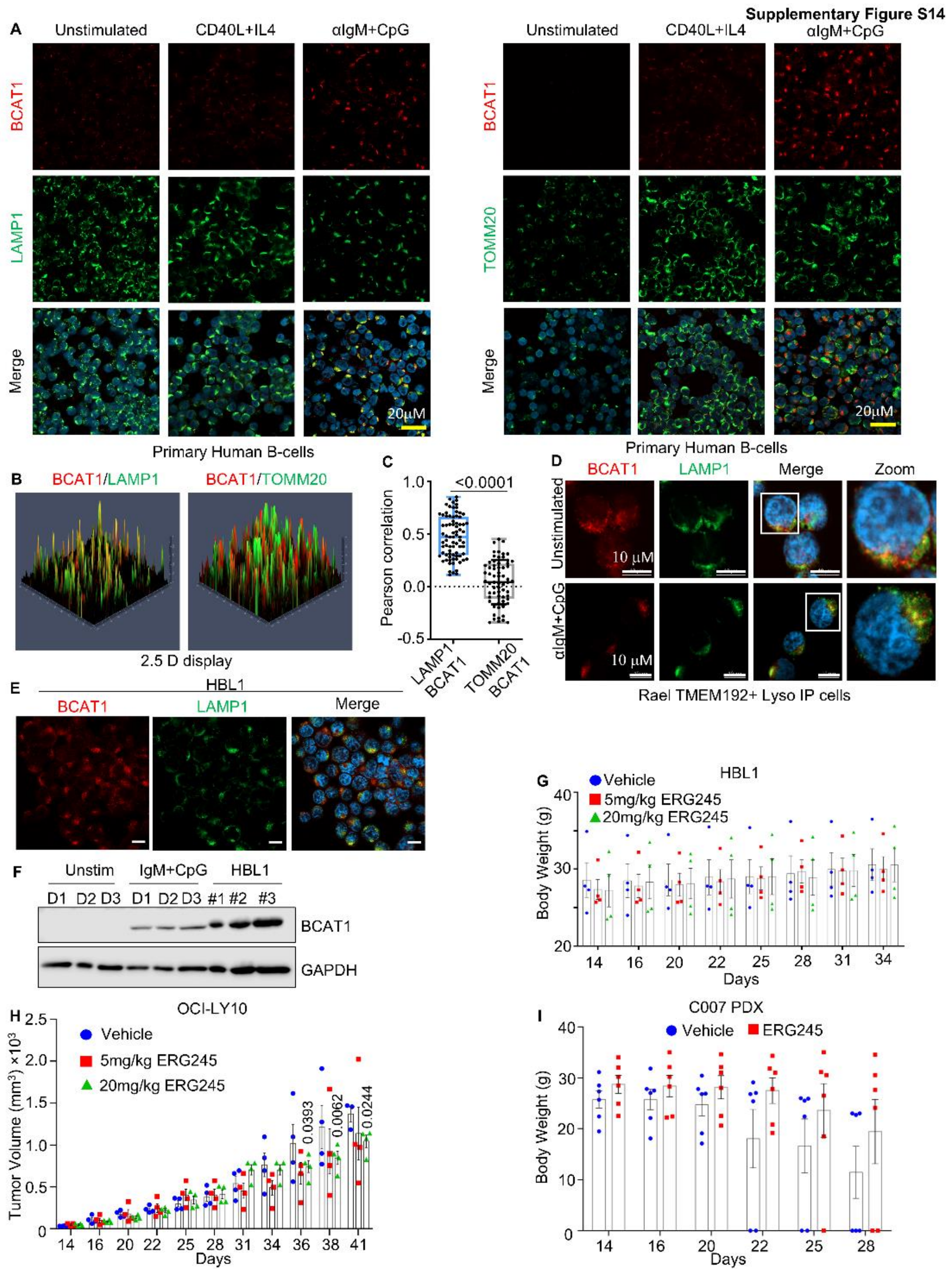

Supplementary Figure S14. BCR/TLR9 induced and lysosomal targeted BCAT1 supports MCD DLBCL xenograft growth in vivo, related to Figures 5 and 6.

(A) Individual IF channels from experiments shown in Figure 5A, from primary B-cells stimulated as indicated for 24 h. Analysis of BCAT1 (red) co-localization with LAMP1 (green, left set of panels) or TOMM20 (green, right set of panels)

(B) 2.5D pixel overlay display of αIgM/CpG stimulated primary B cells from S14A.

(C) Pearson correlation analysis of αIgM/CpG stimulated primary B cells from S14A.

(D) Confocal analysis of BCAT1 and LAMP1 co-localization in Rael TMEM192+ Lyso-IP cells.

(E) Confocal analysis of BCAT1 and LAMP1 co-localization in HBL1 cells.

(F) Immunoblots of WCL from primary B-cells unstimulated or αIgM/CpG stimulated for 24h or from HBL1.

(G) Body weights of mice with HBL1 xenograft implants and treated with saline or ERG245 as indicated. Day 14 was the first day the mice received ERG245 or saline injection.

(H) Tumor volumes of mice with OCI-LY10 xenografts and treated with saline or ERG245, as indicated. Day 14 was the first day the mice received ERG245 or saline injection. P-values were calculated by two-way ANOVA followed by two-stage linear step-up procedure of Benjamini, Krieger and Yekutieli.

(I) Body weights of mice with C007 PDX implants and treated with saline or ERG245 as indicated. Day 14 was the first day the mice received ERG245 or saline injection.

Supplementary Table Legends

Supplementary Table S1. Transcriptomic, proteomic, and metabolomics analysis of human primary B cells simulated by 10 indicated conditions for 24 hours. Related to Figure 1-3 and S1-4.

Supplementary Table S2. Metabolomic analysis of ERG245 vs vehicle treated Rael cells expressing sgRNAs targeting control or BCAT1 and stimulated by αIgM+CpG for 24h. Related to Figure S8.

Supplementary Table S3. RNAseq analysis of primary B-cells electroporated with Cas9 RNPs loaded with control or BCAT1 sgRNAs and stimulated by αIgM+CpG for 24h. Related to Figure 3, S11.

Supplementary Table S4. Ion intensities of 13C carbon labeled metabolites in BCAA and TCA metabolism. Related to Figure 4.

Supplementary Table S5. Metabolomic analysis of ERG245 vs vehicle treated primary B-cells stimulated by αIgM+CpG for 24h. Related to Figure 4.

Supplementary Table S6. Tandem-mass-tag protein abundances in immunopurified lysosomes from Rael Lyso-IP cell unstimulated or stimulated by αIgM+CpG. Raw data was median normalized. Related to Figure 5.

Supplementary Table S7. BCAAs and BCKA ion intensities in whole cell vs lysosomes immunopurified from Rael Lyso-IP cells unstimulated or stimulated by αIgM+CpG. Related to Figure 5. Tandem-mass-tag protein abundances in unstimulated, αIgM+CpG stimulated human naïve B cells or in HBL1 DLBCL. Related to Figure 6.

Supplementary materials and methods

Cell lines and reagents

Rael Burkitt cells, HBL1, OCI-LY10 and TMD8 DLBCL cells were obtained from Ethel Cesarman. HBL1 with stable *Streptococcus pyogenes* Cas9 expression were generated by lentiviral transduction and blasticidin selection (5 μg/ml), as previously described (1). Rael with stable TMEM192-HA were generated by lentiviral transduction using pLJC5-Tmem192-3xHA (Addgene plasmid #102930, a gift from David Sabatini). B-cells were grown in RPMI 1640 medium (Gibco, Life Technologies) with 10% fetal bovine serum (FBS, Gibco) in a humidified incubator at 37°C with 5% CO2 and routinely certified as mycoplasma-free, using the MycoAlert kit (Lonza). 293T were obtained from ATCC and grown in Dulbecco's Modified Eagle's Medium (DMEM) with 10% FBS. For selection of transduced cells, puromycin was added at the concentration of 3 μg/ml. The BCAT1 inhibitor ERG245 was obtained from Adonia E. Papathanassiu and used at 100 μM for *in vitr*o experiments. ERG245 was used at 5mg/kg or 20mg/kg for *in vivo* mouse xenograft experiments, as indicated. R406 (SYK inhibitor, Selleckchem) was used at 1 and 5 μM (2, 3). Ibrutinib (BTK inhibitor, Selleckchem) was used at 2.5 and 12.5 μM (4, 5). ST2825 (MyD88 inhibitor, MedChemExpress) was used at 5 and 20 μM (6, 7). Idelalisib (PI3K inhibitor, Selleckchem) was used at 5 and 10 μM (8, 9). Cyclosporin A (NFAT inhibitor, Selleckchem) was used at 5 and 10 μM (10, 11). IKK-2 inhibitor VIII (IKKβ inhibitor, ApexBio) was used at 2 and 10 μM (12). JAK inhibitor 1 (Sigma) was used at 1.5 and 10 μM (13, 14). SCH772984 (ERK inhibitor, Selleckchem) was used at 10 and 20 μM (15, 16). SP600125 (JNK inhibitor, Selleckchem) was used at 10 and 20 μM (17, 18). SB203580 (p38 inhibitor, Selleckchem) was used at 2.5 and 10 μM (19, 20). Torin 1 (mTOR inhibitor, MedChemExpress) was used at 0.5 and 2 μM (21, 22). Venuloside A (LAT3 inhibitor, MedChemExpress) was used at 10 μM. Sodium pyruvate and lactate (Sigma-Aldrich) were used at 1:9 or at 9:1 mM ratios. Oligomycin was used at 10nM. Piericidin A was used at 100nM. R-2-hydroxyglutarate (R-2HG) and alpha-ketoglutarate (αKG) were used at 100μM. All cell lines were routinely tested for mycoplasma by the Lonza Mycoalert kit, according to the manufacturer's instructions. Antibodies used in the study was listed in Key Resources table.

Antibodies

The following antibodies were used for immunoblot analysis. Anti-DDX1 (Bethyl Laboratories, catalogue (Cat.) #A300-521A), IRF4 (Cell Signaling Technology, Cat. #4964), Fas (4C3) (Cell Signaling Technology, Cat. #8023), Bcl-6 (D-8) (Santa Cruz, Cat. # sc-7388), NAMPT/PBEF (Proteintech, Cat. #11776-1-AP), TRAF1 (45D3) (Cell Signaling Technology, Cat. #4715), PHGDH (Bethyl Laboratories, Cat. #A304-732A), FLIP (G-11) (Santa Cruz, Cat. #sc-5276), Cyclin D3 (DCS22) (Cell Signaling Technology, Cat. #2936), phospho-mTOR (Ser2448) (Cell Signaling Technology, Cat. #2971), mTOR (7C10) (Cell Signaling Technology, Cat. #2983), p70 S6 Kinase (Cell Signaling Technology, Cat. #9202), phospho-p70 S6 Kinase (Thr389) (Cell Signaling Technology, Cat. #9205), AMPKα (D5A2) (Cell Signaling Technology, Cat. #5831), phospho-AMPKα (Thr172) (40H9) (Cell Signaling Technology, Cat. #2535), BCAT1 (Cell Signaling Technology, Cat. #12822), BCAT1 (D6D4K) (Cell Signaling Technology, Cat. #88785), BCAT2 (D8K3O) (Cell Signaling Technology, Cat. #79764), ATF-4 (D4B8) (Cell Signaling Technology, Cat. #11815), anti-puromycin (Millipore, Cat# MABE343), SLC7A5 (Cell Signaling Technology, Cat. 13752-1-AP), LAMP1 (H4A3) (Santa Cruz, Cat. sc-20011), Tom20 (F-10) (Santa Cruz, Cat. sc-17764), alpha tubulin (DM1A) (Abcam, Cat. # ab7291) and GAPDH (D16H11) XP® (Cell Signaling Technology, Cat. #5174). APC-tagged anti-human CD274 (B7-H1, PD-L1) (BioLegend, Cat. #329708) and APC-tagged anti-human CD19 (BioLegend, Cat. #302212) antibodies were used for flow cytometry. HRP-coupled secondary antibodies against mouse IgG (Cell Signaling Technology, Cat. #7076) and rabbit IgG (Cell Signaling Technology, Cat.#7074) were used as secondary antibodies for immunoblot.

B-cell line CRISPR-Cas9 editing

CRISPR/Cas9 engineering was performed using stable Cas9 expression and Broad Institute Brunello library sgRNA sequences. sgRNA oligos were obtained from Integrated DNA Technologies and cloned into the pLentiGuide-Puro vector (Addgene plasmid #52963, a gift from Feng Zhang). Lentiviruses were produced in 293T cells by co-transfection of pLentiGuid-puro with psPAX2 and VSV-G packaging vectors. At 24 hours post transfection, the cell culture media was changed to RPMI-1640+10% FBS. Two rounds of lentiviral transduction were performed at 48 and 72 hours post-transfection. Transduced cells were selected by puromycin (3 μg/ml), added 48 hr post-transduction. Depletion of target gene encoded protein expression was confirmed by immunoblot.

Primary B-cell CRISPR-Cas9 editing using crRNA-tracrRNA-Caspase 9 RNP system

To create a RNP complex for knocking out BCAT1 in human primary-B-cells, a mixture of 2.2 μL crRNA, 2.2 μL tracrRNA, and 5.6 μL duplex buffer was heated to 95°C for 5 minutes and then cooled to room temperature. The crRNA-tracrRNA complex was then combined with 0.6 μL of caspase 9 to assemble the RNP complex, which was incubated for 20 minutes at room temperature. The RNP complex was mixed with washed human primary-B-cells suspended in buffer T, then electroporated using parameters of 1700V, 20ms width, and 1 pulse. Finally, the cells were added to prewarmed media. To enhance the knock-out efficiency, a 1:1 mixture of two BCAT1 crRNA-tracrRNA-Cas9 RNP complexes was electroporated simultaneously.

Immunoblot analysis

Immunoblot was performed as previously described (23). In brief, the same numbers of cells were harvested and lysed. Whole cell lysates (WCL) prepared by boiling cells in 1× Laemmli buffer were separated by SDS-PAGE electrophoresis, transferred onto the nitrocellulose membranes, blocked with 1% BSA in TBST buffer and the probed with relevant primary antibodies at 4 °C overnight, followed by secondary antibody incubation for 1 h at room temperature. Blots were then developed by incubation with ECL chemiluminescence for 1 min (Millipore) and images were captured by Licor Fc platform. Bands intensities were measured where indicated by Image Studio Lite Version 5.2. DDX1, GAPDH, and α-tubulin were used as loading controls. DDX1 was used as a load control in multiple recent papers, as we found that its expression level does not change significantly between resting and activated B-cell states, in particular in the context of Epstein-Barr virus transformation of resting B-cells into lymphoblastoid cells or following immunoreceptor stimulation (24-26).

Puromycin analysis of protein translation.

Two million cells were seeded at 0.3 million per ml in RPMI-1640+10% FBS. Puromycin (10 μg/ml) was added for 20 min at 37°C. WCLs were prepared and analyzed by immunoblot, using an anti-puromycin monoclonal antibody to visualize newly synthesized polypeptides.

Flow cytometry analysis

Flow cytometry was performed on a BD FacsCalibur instrument. For intracellular cytokine staining, 1× 10^6 cells were treated with GolgiPlug and GolgiStop (BD) for 5 h. Subsequently, cells were fixed and permeabilized using eBioscience™ Intracellular Fixation & Permeabilization Buffer Set, followed by staining with αIL-10 primary antibody for 30 minutes on ice. Labeled cells were then washed three times with FACS buffer prior to the flow cytometry. For CFSE staining, primary B-cells were stained with 10 µM CFSE for 15 minutes at 37°C, washed, resuspended at 100,000 cells/mL and then treated with indicated conditions. 2NBDG (2-(N-(7-nitrobenz-2-oxa-1,3-diazol-4-yl)amino)-2-deoxyglucose; ThermoFisher) was used to assess the real-time glucose uptake of human primary-B-cells by flow cytometry, as described previously (24). eBioscience™ Cell Proliferation Dye eFluor™ 670 (5uM) was used for measuring total protein content in human primary-B-cells following the manuals. Cells were then analyzed by FACS. FACS data were analyzed with FlowJo V10.

Growth curve analysis and caspase activation assay

Cells were counted and then normalized to the same starting concentration, using the CellTiterGlo (CTG) luciferase assay (Promega, Cat#G7570). Live cell numbers were quantitated at each timepoint by CTG measurements, and values were corrected for tissue culture passage. Fold change of live cell number at each timepoint was calculated as a ratio of the value divided by the input value. For long-term assays, chemicals were refreshed at every 72 hours in fresh media. Caspase 3/7 activity was quantified by Caspase-Glo assays (Promega) according to manufacturer's manual, and normalized to the cell number of the same sample determined by CTG assay. All values were quantitated on a Molecular Devices plate reader.

RNAseq analysis

Total RNA was isolated by the RNeasy Mini kit (Qiagen), following the manufacturer's manual. An in-column DNA digestion step was included to remove the residual genomic DNA contamination. To construct indexed libraries, 1 µg of total RNA was used for polyA mRNA-selection, using the NEBNext Poly(A) mRNA Magnetic Isolation Module (New England Biolabs), followed by library construction via the NEBNext Ultra RNA Library Prep Kit (New England Biolabs). Each experimental treatment was performed in triplicate. Libraries were multi-indexed, pooled and sequenced on an Illumina NextSeq 500 sequencer using single-end 75 bp reads (Illumina) at the Dana Farber Molecular Biology core. Adaptor-trimmed Illumina reads

for each individual library were mapped back to the human GRCh37.83 transcriptome assembly or EBV Akata genome (accession#: KC207813.1) using STAR2.5.2b (27). Feature Counts was used to estimate the number of reads mapped to each contig (28). Only transcripts with at least 5 cumulative mapping counts were used in this analysis. DESeq2 was used to evaluate differential expression (DE) (29). DESeq2 uses a negative binomial distribution to account for overdispersion in transcriptome datasets. It uses a conservative analysis that relies on a heuristic approach. Each DE analysis used pairwise comparison between the experimental and control groups. Differentially expressed genes were identified and a p values < 0.05 and absolute fold change > 2 cutoff was used. Differentially expressed genes were subjected to Enrichr analysis which was employed to perform gene list-based gene set enrichment analysis on the selected gene subset. The algorithm used to calculate combined scores was described previously (30). P value and log2 fold change were generated with DESeq2 under default settings with Wald test and normal shrinkage, respectively. Top 5 Enrichr terms that passed the adjusted p-value cutoff were visualized using Graphpad Prism 7.

Volcano plots were built with Graphpad Prism7. Heatmaps were generated by feeding Z-score values of selected EBV genes from DESeq2 into Morpheus software (https://software.broadinstitute.org/morpheus/).

Confocal microscopy

Cells were seeded on glass slides in PBS, air dried and then fixed with 4% paraformaldehyde (PFA) in PBS for 10 minutes. PFA was removed and fixed cells were permeabilized with 0.1% Triton-X in PBS. Slides were blocked with 1% IgG-free BSA (Sigma-Aldrich, Cat# A2058) in PBS for 30 minutes at room temperature. Cells were incubated with primary antibodies against BCAT1 (Cell Signaling, 1:200) and LAMP1 (Santa Cruz, 1:100) or TOMM20 (Santa Cruz, 1:100) in PBS containing 1% BSA for 1 hour at 37°C. Slides were then washed three times and then incubated with secondary antibodies (Alexa Fluor 488-conjugated goat anti-mouse and Alex Fluor 594-conjugated goat anti-rabbit, diluted 1:250 in PBS) for 1 hour at 37°C. Slides were washed three times in PBS and incubated with 100 uL of Hoechst 33258 (10 µg/mL in PBS) for 10 minutes. Cells were then washed three times with PBS. ProLong Gold anti-fade was applied to the slide, which was then sealed with a No. 1.5 coverslip. Image acquisition was performed

with the Zeiss LSM 800 instrument. Image analysis was performed with the Zeiss ZEN Blue software.

Lysosomal immunopurification (LysoIP)

20 million cells were collected for each LysoIP. The cells were rinsed with PBS twice, then centrifuged for 2 minutes at 1000 g at 4°C, resuspended in 1 mL KPBS (136 mM KCl, 10 mM KH2PO4, pH 7.25 and was adjusted with KOH).The cells were homogenized with 55 strokes of a 2 mL homogenizer. The homogenized mixture was centrifuged for 2 minutes at 1000 g at 4°C. Anti-HA magnetic beads were prewashed with KPBS. 50 µL of the beads were placed in a 1.5 mL tube, mixed with 175 µL KPBS, and then separated using a magnetic stand. The supernatant was incubated with 25 µL of the KPBS prewashed anti-HA magnetic beads for 15-30 minutes (3 minutes for metabolite extraction). Immunoprecipitated lysosmes were washed 3 times with 300 µL KPBS using a DynaMag Spin Magnet. All buffers used in the experiment were pre-chilled on ice. For immunoblot analysis 100 uL of SDS-PAGE sample buffer was added to the tube and mixed with vortexing and boiled for 10min. For proteomics analysis, proteins from the isolated lysosomes were extracted by 5% formic acid. For targeted metabolomics, lysosomal metabolites were extracted with dry-ice cold 80% methanol.

Intracellular metabolite profiling

For profiling for 10 stimulation conditions of human primary-B-cells, 5×10^6 cells were seeded into a T25 flask with 10 mL of fresh RPMi-1640, supplemented with 10% FBS. 24 hours after seeding, 3×10^6 cells were pelleted and resuspended in fresh media for additional 3 hours prior to intracellular metabolite extraction.

For profiling of ERG245 treated human primary-B-cells, 5×10^6 cells were treated with vehicle or 100µM ERG245 1 hour prior to αIgM+CpG or CD40L+IL4 stimulation. At 24h post stimulation, 3×10^6 live cells from each condition were pelleted and resuspended in fresh RPMI-1640 media, supplemented with 10% dialyzed FBS for 3 hours prior to intracellular metabolite extraction.

Cells were pelleted and washed with 5 mL of room temperature PBS. Pellets were resuspended in 1 mL of dry ice cold 80% methanol, incubated at -80°C for 30 minutes and centrifuged at 21,000 x g for 5 minutes to precipitate proteins. For mice xenograft tumors, 100mg of tumor tissues were washed once with PBC and submerged in 500 µl of 80% (vol/vol) HPLC-grade

methanol (cooled to −80 °C). The tissues were smashed/grinded for 1–2 min with small pestle/tissue grinder on dry ice in the tube, vortex for 1 min at 4–8 °C and incubate for 4 hours at −80 °C. The tissues were then centrifuged at 21,000g for 10 min using a refrigerated centrifuge (4–8 °C). The supernatant was collected in pre-chilled tubes and stored at -80°C. On the day of analysis, supernatants were incubated on ice for 20 minutes and clarified by centrifugation at 21,000 x g at 4°C. At the Beth Israel Mass Spectrometry core, supernatants were dried down in a speed vacuum concentrator (Savant SPD 1010, Thermofisher Scientific) and re-suspended in 100µL of 60/40 acetonitrile/water. The samples were then vortexed, sonicated in ice-cold water for 1 minute, and incubated on ice for 20 minutes. Supernatants were collected in an autosampler vial after centrifugation at 21,000 x g for 20 minutes at 4°C. Pooled QC samples were generated by combining ~15µL of each sample. Metabolite profiling was performed using Dionex Ultimate 3000 UHPLC system coupled to Q-Exactive plus orbitrap mass spectrometer (ThermoFisher Scientific, Waltham, MA) with an Ion Max source and HESI II probe operating in switch polarity mode. A zwitterionic Sequent zic philic column (150 x 2.1mm, 5µm polymer, part # 150460, MilliporeSigma, Burlington, MA) was used for polar metabolite separation. Mobile phase A (MPA) was 20mM ammonium carbonate in water, pH9.6 (adjusted with ammonium hydroxide) and MPB was acetonitrile. The column was held at 27°C, injection volume 5µL, autosampler temperature 4°C and LC conditions at flow rate of 0.15 mL/min were: 0min: 80% B, 0.5min: 80% B, 20.5min: 20% B, 21.3min: 20%B, 21.5min: 80% B with 7.5min of column equilibration time. MS parameters were: sheath gas flow 30, aux gas flow 7, sweep gas flow 2, spray voltage 2.80kV for negative & 3.80kV for positive, capillary temperature 310°C, S-lens RF level 50 and aux gas heater temp 370°C. Data acquisition was done using Xcalibur 4.1 (ThermoFisher Scientific) and performed in full scan mode with a range of 70-1000m/z, resolution 70,000, AGC target 1e6 and maximum injection time of 80ms. Data analysis was performed in Compound Discoverer 3.1 and Tracefinder 4.1. Samples were injected in randomized order and pooled QC samples were injected regularly throughout the analytical batch. Metabolite annotation was done base on accurate mass (±5ppm) and matching retention time (±0.5min) as well as MS/MS fragmentation pattern from the pooled QC samples against in-house retention time +MSMS library of reference chemical standards. Metabolites with CV<30% in pooled QC were used for the statistical analysis. The quality of integration for each metabolite peak was reviewed.

Metabolites with p-values < 0.05, $\log_2$(fold change)>1 or <-1 were used for pathway analysis using MetaboAnalyst 5.0 (https://www.metaboanalyst.ca/MetaboAnalyst/ModuleView.xhtml).

BCAT1 Isotope Tracing

B-cells were treated with ERG245 for 1 hour prior to αIgM+CpG stimulation. 24hr later, cells were incubated with 2 mM of $^{15}N_2$-Glutamine and 381 μM of $^{13}C_6$-leucine for 8, 16 and 24 hours. Equal number of cells were spun down for 5 minutes at room temperature and the supernatant was aspirated off. Metabolism was quenched by addition of 1 mL of dry ice cold methanol. Samples were vortexed upon methanol addition. Samples were stored at -80°C until analysis. On the day of analysis, samples were thawed on ice and then centrifuged at 21,000g and 4°C for 20 minutes. Supernatants were dried down using the Genevac EZ-2 elite vacuum dryer. Dried extracts were re-suspended in 120μL of water, vortexed, sonicated for 2 minutes in an ice-cold water bath, and clarified by spinning down at 21,00g for 20 minutes at 4°C. 5 μL of supernatant was injected onto a HSS-T3 column (2.1x100 mm, 1.8μM, part # 186003539 Waters Corporation). Column oven and autosampler temperatures were 30°C and 4°C respectively. The mobile phase A (MPA) was 0.1% formic acid in water and mobile phase B (MPB) was 0.1% formic acid in methanol. LC gradient conditions at a flow rate of 0.3mL/minute were: 0 min: 2.5% B, 1.5 min: 2.5%B, 8 min- 65%B, 9- 90% B, 10.8 min: 90% B, 11min : 2.5% B and 15 min : 2.5 % B. Metabolite detection was performed using a Dionex Ultimate 3000 UHPLC system coupled to a Q-Exactive Plus orbitrap mass spectrometer (ThermoFisher Scientific, Waltham, MA) with an Ion Max source and HESI II probe operating in polarity switching mode. MS parameters were: sheath gas flow = 30, aux gas flow = 7, sweep gas flow = 2, spray voltage = 2.80 for negative & 3.30 for positive ion modes, capillary temperature = 310°C, S-lens RF level=50, aux gas heater temp: 370°C. Data acquisition was performed using an Xcalibur 4.1 (ThermoFisher Scientific) in full scan mode with a range of 70-1000m/z, a resolving power of 70,000, an AGC target of 1×10^6 and maximum injection time of 100 ms. Data analysis was done using Tracefinder4.1 software. Natural abundance correction was performed using IsoCorrectoR (Heinrich et al., 2018)

Proteomics LC-MS/MS analysis

Sample Preparation for Mass Spectrometry Experiments (10 conditions)

Samples were prepared for LC-MS/MS experiments are described previously1. Clarified flash-frozen lysates were thawed and reduced by the addition of 5mM tris(2-carboxyethyl)-phosphine (TCEP) and reactive cysteines were blocked by alkylation through the addition of iodoacetamide (IAA) to a final concentration of 10mM. To prevent overalkylation, the reaction was quenched by the addition dithiothreitol (DTT) to a concentration of 15mM. A trigger channel was created by mixing 10uL from each lysate. Samples were then precipitated by the chloroform-methanol method.

Precipitated treated lysates were digested overnight with Lys-C (Wako), carried out at room temperature, at an approximated 1:100 protease:protein. This was followed by the addition of sequencing grade Trypsin (Promega) at 37°C for 6 hours for further proteolytic digestion. Digested samples were labelled at room temperature with TMT-11 reagents for 90 minutes and then quenched with hydroxylamine. To check labelling efficiency and overall relative sample abundance, 2µL from each TMT-labelled sample were combined to create a ratio check as samples were normalized by cell count. Samples were then combined with equal volume following the assumption of equal cell counts except for the µIgM+CpG sample. Review of the ratio check indicated that the sample treated with µIgm+CpG was over 4x more abundant than the least abundant sample. As such, the amount of µIgM+CpG sample added to the final combination was halved to reduce the effects of compositional proteomics2.

The combined TMT-labelled sample was then flash-frozen and dried down by vacuum centrifugation before resuspension in a formic acid solution such that pH of sample was below 3 (approximate final concentration of formic acid was 3%). The acidified sample was then desalted through a C18 solid phase extraction column (Waters) and dried down by vacuum centrifugation. Samples were resuspended in a 10mM ammonium bicarbonate and 5% acetonitrile solution for off-line basic pH reversed phase (BPRP) fractionation.

Sample Preparation for Mass Spectrometry Experiments (3 conditions)

Samples were prepared for LC-MS/MS analysis as described above except samples were normalized to total protein amount. A protein bicinchoninic acid assay was used on clarified lysate before reduction and alkylation. Additionally, after labelling samples with TMT-11

reagents, samples were normalized based on ratio check data to ensure a 1:1:1:1:1:1:1:1:1 total protein ratio was observed as described previously1.

Off-line BPRP Fractionation

TMT-labelled samples suspended in 10mM ammonium bicarbonate and 5% acetonitrile were subjected to off-line BPRP high performance liquid chromatography on an Agilent 1100 pump with degaser and photodiode array detector. Elution occurred over a 13-37% acetonitrile 50-minute gradient in 10mM ammonium bicarbonate. The instrument separated the TMT-labelled sample into 96 fractions, collected in plate for by an automated fraction collector. To reduce redundancy, the 96 fractions were combined into 24 fractions as described previously. These 24 fractions were dried down by vacuum centrifugation and resuspended in 1% formic acid. A final cleanup step was performed by desalting each fraction over C18 stop-and-go extraction tips (STAGE tips), before resuspension in a loading buffer comprised of 5% acetonitrile and 5% formic acid.

LC-MS/MS Experiments

For each BPRP fractionated sample, 12 of the 24 fractions were selected for LC-MS/MS analysis to reduce redundant identifications. Each fraction was analyzed on a Thermo Orbitrap Fusion Lumos with a Proxeon EASY-nLC 1200 system before the source (Thermo Fisher Scientific, San Jose). The mass spectrometer was operated in a data-dependent centroid mode for all SPS-MS3 methods. On-line chromatography was performed on a 100μm inner diameter microcapillary column packed with 35cm of Accucore C18 resin was used. Approximately 2μg of labelled peptides were loaded onto the column. Spectra were acquired across a 90-minute LC gradient ranging from 6-25% acetonitrile in 0.125% formic acid. For the 10-condition experiment, a real-time search method was employed in the instrument such that SPS-MS3 scans would not trigger unless a successful Comet search result was obtained from a preceding MS2 scan3. For the 3-condition experiment, in lieu of real-time search a field asymmetric wave form ion mobility spectrometry (FAIMS) device was attached ahead of the mass spectrometer's source. The FAIMS was operated as described previously with 3 CV offsets set at -40, -60, and -80; SPS-MS3 scans were triggered with a traditional Top 10 protocol4. MS1 scans were obtained (Orbitrap resolution: 120,000; mass range: 400-1400 m/z; Automatic Gain Control (AGC) target = 200,000, maximum ion injection time: 100ms) prior to a low-resolution MS2 scan utilizing CID

(Collision energy: 35%; maximum injection time: 35ms; AGC target: 20,000; Isolation window: 0.7 Th). Precursors from the MS1 spectra were selected for MS2 by a Top 10 method. In the case of the 10-condition experiment, MS3 scans were sent from the real-time search algorithm. For the 3-condition experiment, the SPS-MS3 scan encoded into the method was a Top 10 method (Orbitrap Resolution: 50,000; Scan Range: 100-500 m/z; maximum injection time: 120ms; AGC Target: 150,000).

LC-MS/MS Data Analysis

Spectra from all mass spectrometry experiments were processed and searched with an in-house Sequest-based software pipeline as described previously1,5,6. Raw data acquired on mass spectrometer instruments were converted into an mzXML format before a Sequest search was executed against a human proteome database (Uniprot Database ID: 9606, downloaded February 4, 2014). This database was concatenated with common laboratory contaminants and a database comprised of all protein sequences reversed. Ion tolerances for precursor and product ions were set to 50ppm and 0.9 Da respectively. A variable mass difference of +15.99491 Da was assigned to methionine residues to account for potential oxidation. Additionally, fixed modifications on cysteine (+57.02146 Da) and lysine and the peptide N-terminus (+229.16293 Da) were assigned to account for protective alkylation and TMT-11 labelling, respectively.

The peptide level false discovery rate (FDR) was determined using the target-decoy method and controlled to 1% by linear discriminant analysis before protein level FDR correction to 1% was performed using the principles of parsimony as described previously1,6–8. Signal-to-noise ratios (SNR) for TMT reporter ions were extracted from high resolution SPS-MS3 scans. A quality control filter of 200 SNR and isolation specificity, a measure of a scan's isolation purity, of 0.5 were imposed on the data for each experiment9. Protein level estimation for each sample was determined as the sum SNR of the appropriate TMT reporter-ion channel from all contributing peptides identified and quantified in the experiment. Raw data was median normalized. For the 10-condition experiment, protein level abundances were normalized across all TMT-reporter ion channels under the assumption that total protein levels were consistent across all TMT-channels before an additional normalization step ensuring that protein levels of SORL1, SUDS3, GRAP, CHMP1B, TCL1A, MECP2, ABHD10, STAP1, and PSIP1 were consistent across all TMT-channels based on RNAseq data. For the 3-condition experiment, protein level abundances were

normalized across all TMT-reporter ion channels under the assumption that total protein levels were consistent across all TMT-channels.

Relative protein ratios were calculated between protein TMT reporter-ion SNR levels in a patient-matched fashion before averaging across the calculated ratios. When comparing patient samples to laboratory cell lines (in the case of the 3-conditoin experiment) protein TMT reporter-ion SNR levels were averaged before calculating a ratio. Two-factor analysis of variance was used to calculate significance to account for patient-to-patient variation. Tukey's Honestly Significant Difference post-hoc tests were then used to identify nominal p-values between pairwise comparisons.

Antigen tetramer preparation

Ovalbumin conjugated to biotin (with EZ-Link Sulfo–NHS–LC–Biotinylation kit; Thermo Scientific, #21435) or Biotinylated HEL (GTX82960-pro) was incubated with streptavidin in 4:1 molar ratio for 30 mins on ice. Tetramer antigens were purified using 100kDa size-exclusion centrifugal device (Amicon Ultra centrifugal filter, # UFC510008).

***In vitro* treatment of murine splenic B cells**

Spleens were harvested from C57BL/6J or MD4 mice and processed into single cells. Briefly, splenic cells were passed through 70μm cell strainer, pelleted, treated with 1 mL RBC lysis for 5 mins at room temperature, and washed in 10mL PBS. B cells were enriched with CD45R/B220 microbeads (Miltenyi Biotec: #130-049-501). B cells were treated with: murine CD40L 50ng/ml (Biolegend, #797404), CpG 0.5μM (IDT), murine IL4 (Biolegend, #574302) 20ng/ml, αIgM (Sigma, #M8644) at 1μg/ml, LPS (Sigma, #L2018) at 10 μg/ml, OVA and HEL at 1 μg/ml for 48 hours.

In vivo murine B-cell stimulation

Female C57BL/6J mice were divided into six treatment groups based on T cell-dependent and T-cell independent pathways: (i) 20μg LPS + 250μg anti-IgM representing T cell-independent with BCR signaling pathway (ii) 1μg IL4 in complex with 10ug αIL4 (Biolegend, #574302) (31, 32) + 250μg agonistic anti-CD40 (BioXcell, BE0016-2) to activate a T cell-dependent pathway (iii) PBS only (iv) 20μg LPS + 250μg αIgM + ERG245 (20mg/kg) (v) 1μg IL4 in complex with

10μg αIL4 + 250μg αCD40 + BCAT1 inhibitor (vi) PBS + ERG245. Complexes of IL4 and αIL4 were made to increase the IL4 half-life *in vivo* (31). Mice were exposed to treatment for 48 hours before the spleen was harvested from each mouse and prepared as single cell suspension.

Female MD4 mice were also grouped into six treatment categories: (i) 20μg LPS + 10μg hen egg lysozyme (HEL) (ii) 1μg IL4 in complex with 10ug αIL4 + 250μg αCD40 + 10μg ovalbumin (iii) PBS only (iv) 20μg LPS + 10μg HEL+ ERG245 (iv) 1μg IL4 in complex with10ug αIL4 + 250μg αCD40 + 10μg ovalbumin + ERG245 inhibitor (vi) PBS + ERG245. All treatments were prepared in 500ul PBS and injected through the intraperitoneal (IP) route. Mice were exposed to treatment for 48 hours before the spleen was harvested from each mouse and prepared as single cell suspension.

Mouse xenograft experiments

Mouse xenograft experiments were done in accordance with the Institutional Animal Care & Use Committee (IACUC# 2017-0035) of Weill Cornell Medical Center (WCMC).

Twelve NOD.Cg-Prkdcscid Il2rgtm1Wjl/SzJ (NSG) immunocompromised mice were subjected to bilateral xenotransplantation with a finely minced 2-3mm^3 piece of MCD DLBCL, HBL1 for two weeks. Once the tumor volumes reached the range between 30-60mm^3, mice were randomly divided into 3 groups. Vehicle, 5mg/kg, or 20mg/kg ERG245 was injected intraperitonially, once a week, into the mice in the three groups respectively. Tumor growth was monitored for an additional 3 weeks.

Twelve NOD.Cg-Prkdcscid Il2rgtm1Wjl/SzJ (NSG) immunocompromised mice were subjected to bilateral xenotransplantation with a finely minced 2-3mm^3 piece of MCD DLBCL, OCI-LY10 for two weeks. Once the tumor volumes reached the range between 30-60mm^3, mice were randomly divided into 3 groups. Vehicle, 5mg/kg, or 20mg/kg ERG245 was injected intraperitonially, once a week, into the mice in the three groups respectively. Tumor growth was monitored for an additional 4 weeks.

Twelve NOD.Cg-Prkdcscid Il2rgtm1Wjl/SzJ (NSG) immunocompromised mice were subjected to bilateral xenotransplantation with a finely minced 2-3mm3 piece of patient derived MCD DLBCL tumor C007 for two weeks. Once the tumor volumes reached the range between 30-60mm^3, mice

were randomly divided into 2 groups. Vehicle or 20mg/kg ERG245 was injected intraperitonially, three times a week, into the mice in the two groups respectively. Tumor growth was monitored for an additional 2 weeks.

Digital caliper measurements and body weight were measured under 2.5% isoflurane anesthesia. Two-dimensional length (L) and width (W) measurements were used to extrapolate 3D volume using the formula (L x W^2)/2. All animals were euthanized by CO2 asphyxiation at a flow rate 3.5L/min.

Seahorse metabolic analysis

For mitochondrial stress test analysis, cell culture plates were layered with Cell-Tak to enable B-cell adhesion, and cells were then seeded at 500,000 per well. For standard measurements, complete bicarbonate-free RPMI-1640 supplemented with 25 mM HEPES, 10% dialyzed FBS and 2 mM L-glutamine was used as the growth media during the period of data acquisition. Detection of changes in oxygen consumption and extracellular acidification rates was achieved with the use of Seahorse XFe96 sensor cartridges. The following mitochondrial poisons were used: 3.5 μM oligomycin, 2 μM CCCP and 100 nM piericidin A. Data acquisition was performed with the Seahorse XFe96 Analyzer (Agilent).

References

1. Ma Y, Walsh MJ, Bernhardt K, Ashbaugh CW, Trudeau SJ, Ashbaugh IY, et al. CRISPR/Cas9 Screens Reveal Epstein-Barr Virus-Transformed B Cell Host Dependency Factors. *Cell Host & Microbe.* 2017;21(5):580-91.e7.
2. Koerber RM, Held SAE, Heine A, Kotthoff P, Daecke SN, Bringmann A, et al. Analysis of the anti-proliferative and the pro-apoptotic efficacy of Syk inhibition in multiple myeloma. *Exp Hematol Oncol.* 2015;4:21.
3. Borge M, Remes Lenicov F, Nannini PR, de los Rios Alicandu MM, Podaza E, Ceballos A, et al. The expression of sphingosine-1 phosphate receptor-1 in chronic lymphocytic leukemia cells is impaired by tumor microenvironmental signals and enhanced by piceatannol and R406. *J Immunol.* 2014;193(6):3165-74.
4. Puig de la Bellacasa R, Roue G, Balsas P, Perez-Galan P, Teixido J, Colomer D, et al. 4-Amino-2-arylamino-6-(2,6-dichlorophenyl)-pyrido[2,3-d]pyrimidin-7-(8H)-ones as BCR kinase inhibitors for B lymphoid malignancies. *Eur J Med Chem.* 2014;86:664-75.
5. Zhao D, Huang S, Qu M, Wang C, Liu Z, Li Z, et al. Structural optimization of diphenylpyrimidine derivatives (DPPYs) as potent Bruton's tyrosine kinase (BTK) inhibitors with improved activity toward B leukemia cell lines. *Eur J Med Chem.* 2017;126:444-55.
6. Taniguchi S, Matsui T, Kimura K, Funaki S, Miyamoto Y, Uchida Y, et al. In vivo induction of activin A-producing alveolar macrophages supports the progression of lung cell carcinoma. *Nat Commun.* 2023;14(1):143.
7. Zhang J, Liu Y, Chen H, Yuan Q, Wang J, Niu M, et al. MyD88 in hepatic stellate cells enhances liver fibrosis via promoting macrophage M1 polarization. *Cell Death Dis.* 2022;13(4):411.
8. Luk SK, Piekorz RP, Nurnberg B, and Tony To SS. The catalytic phosphoinositol 3-kinase isoform p110delta is required for glioma cell migration and invasion. *Eur J Cancer.* 2012;48(1):149-57.
9. Meadows SA, Vega F, Kashishian A, Johnson D, Diehl V, Miller LL, et al. PI3Kdelta inhibitor, GS-1101 (CAL-101), attenuates pathway signaling, induces apoptosis, and overcomes signals from the microenvironment in cellular models of Hodgkin lymphoma. *Blood.* 2012;119(8):1897-900.
10. Condello M, Cosentino D, Corinti S, Di Felice G, Multari G, Gallo FR, et al. Voacamine modulates the sensitivity to doxorubicin of resistant osteosarcoma and melanoma cells and does not induce toxicity in normal fibroblasts. *J Nat Prod.* 2014;77(4):855-62.
11. Juvale K, and Wiese M. 4-Substituted-2-phenylquinazolines as inhibitors of BCRP. *Bioorg Med Chem Lett.* 2012;22(21):6766-9.
12. Wang C, Jiang S, Ke L, Zhang L, Li D, Liang J, et al. Genome-wide CRISPR-based gene knockout screens reveal cellular factors and pathways essential for nasopharyngeal carcinoma. *J Biol Chem.* 2019;294(25):9734-45.

13. Ghazawi FM, Faller EM, Sugden SM, Kakal JA, and MacPherson PA. IL-7 downregulates IL-7Ralpha expression in human CD8 T cells by two independent mechanisms. *Immunol Cell Biol.* 2013;91(2):149-58.
14. Ettich J, Wittich C, Moll JM, Behnke K, Floss DM, Reiners J, et al. Respiratory syncytial virus-approved mAb Palivizumab as ligand for anti-idiotype nanobody-based synthetic cytokine receptors. *J Biol Chem.* 2023;299(11):105270.
15. Ward RA, Colclough N, Challinor M, Debreczeni JE, Eckersley K, Fairley G, et al. Structure-Guided Design of Highly Selective and Potent Covalent Inhibitors of ERK1/2. *J Med Chem.* 2015;58(11):4790-801.
16. Ward RA, Bethel P, Cook C, Davies E, Debreczeni JE, Fairley G, et al. Structure-Guided Discovery of Potent and Selective Inhibitors of ERK1/2 from a Modestly Active and Promiscuous Chemical Start Point. *J Med Chem.* 2017;60(8):3438-50.
17. Lin HY, Shen SC, Lin CW, Wu MS, and Chen YC. Cobalt protoporphyrin inhibition of lipopolysaccharide or lipoteichoic acid-induced nitric oxide production via blocking c-Jun N-terminal kinase activation and nitric oxide enzyme activity. *Chem Biol Interact.* 2009;180(2):202-10.
18. Wang Y, Bell JC, Keeney DS, and Strobel HW. Gene regulation of CYP4F11 in human keratinocyte HaCaT cells. *Drug Metab Dispos.* 2010;38(1):100-7.
19. Davis T, Bagley MC, Dix MC, Murziani PG, Rokicki MJ, Widdowson CS, et al. Synthesis and in vivo activity of MK2 and MK2 substrate-selective p38alpha(MAPK) inhibitors in Werner syndrome cells. *Bioorg Med Chem Lett.* 2007;17(24):6832-5.
20. Zhong C, Liu XH, Chang J, Yu JM, and Sun X. Inhibitory effect of resveratrol dimerized derivatives on nitric oxide production in lipopolysaccharide-induced RAW 264.7 cells. *Bioorg Med Chem Lett.* 2013;23(15):4413-8.
21. Ho TLF, Lee MY, Goh HC, Ng GYN, Lee JJH, Kannan S, et al. Domain-specific p53 mutants activate EGFR by distinct mechanisms exposing tissue-independent therapeutic vulnerabilities. *Nat Commun.* 2023;14(1):1726.
22. Cao Y, Chen M, Tang D, Yan H, Ding X, Zhou F, et al. The proton pump inhibitor pantoprazole disrupts protein degradation systems and sensitizes cancer cells to death under various stresses. *Cell Death Dis.* 2018;9(6):604.
23. Ma Y, Walsh MJ, Bernhardt K, Ashbaugh CW, Trudeau SJ, Ashbaugh IY, et al. CRISPR/Cas9 Screens Reveal Epstein-Barr Virus-Transformed B Cell Host Dependency Factors. *Cell Host Microbe.* 2017;21(5):580-91 e7.
24. Wang LW, Shen H, Nobre L, Ersing I, Paulo JA, Trudeau S, et al. Epstein-Barr-Virus-Induced One-Carbon Metabolism Drives B Cell Transformation. *Cell Metab.* 2019;30(3):539-55 e11.
25. Wang LW, Wang Z, Ersing I, Nobre L, Guo R, Jiang S, et al. Epstein-Barr virus subverts mevalonate and fatty acid pathways to promote infected B-cell proliferation and survival. *PLoS Pathog.* 2019;15(9):e1008030.
26. Liang JH, Wang C, Yiu SPT, Zhao B, Guo R, and Gewurz BE. Epstein-Barr Virus Induced Cytidine Metabolism Roles in Transformed B-Cell Growth and Survival. *mBio.* 2021;12(4):e0153021.
27. Dobin A, Davis CA, Schlesinger F, Drenkow J, Zaleski C, Jha S, et al. STAR: ultrafast universal RNA-seq aligner. *Bioinformatics (Oxford, England).* 2013;29(1):15-21.

28. Liao Y, Smyth GK, and Shi W. featureCounts: an efficient general purpose program for assigning sequence reads to genomic features. *Bioinformatics (Oxford, England).* 2014;30(7):923-30.
29. Love MI, Huber W, and Anders S. Moderated estimation of fold change and dispersion for RNA-seq data with DESeq2. *Genome biology.* 2014;15(12):550.
30. Chen EY, Tan CM, Kou Y, Duan Q, Wang Z, Meirelles GV, et al. Enrichr: interactive and collaborative HTML5 gene list enrichment analysis tool. *BMC bioinformatics.* 2013;14:128.
31. Finkelman FD, Madden KB, Morris SC, Holmes JM, Boiani N, Katona IM, et al. Anti-cytokine antibodies as carrier proteins. Prolongation of in vivo effects of exogenous cytokines by injection of cytokine-anti-cytokine antibody complexes. *J Immunol.* 1993;151(3):1235-44.
32. Shintani Y, Ito T, Fields L, Shiraishi M, Ichihara Y, Sato N, et al. IL-4 as a Repurposed Biological Drug for Myocardial Infarction through Augmentation of Reparative Cardiac Macrophages: Proof-of-Concept Data in Mice. *Sci Rep.* 2017;7(1):6877.
