# Supplementary material for: Multiomic analysis reveals a key BCAT1 role in mTOR activation by B cell receptor and TLR9
Source: J Clin Invest. 2025 Sep 9;135(22):e186258. doi: 10.1172/JCI186258 (PMC12618069; doi:10.1172/JCI186258)

Fig. 3I

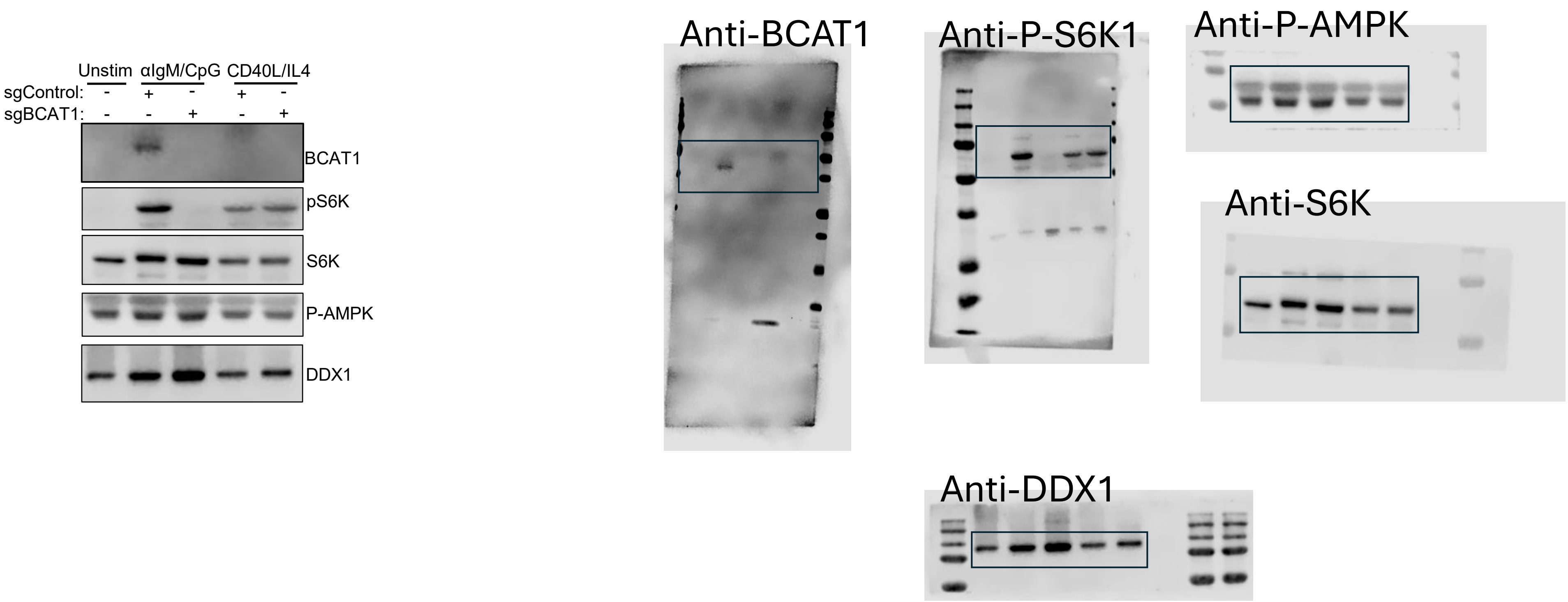

Unstim
αIgM/CpG
CD40L/IL4
sgControl:
sgBCAT1:
BCAT1
pS6K
S6K
P-AMPK
DDX1
Anti-BCAT1
Anti-P-S6K1
Anti-P-AMPK
Anti-S6K
Anti-DDX1

Fig. 3J

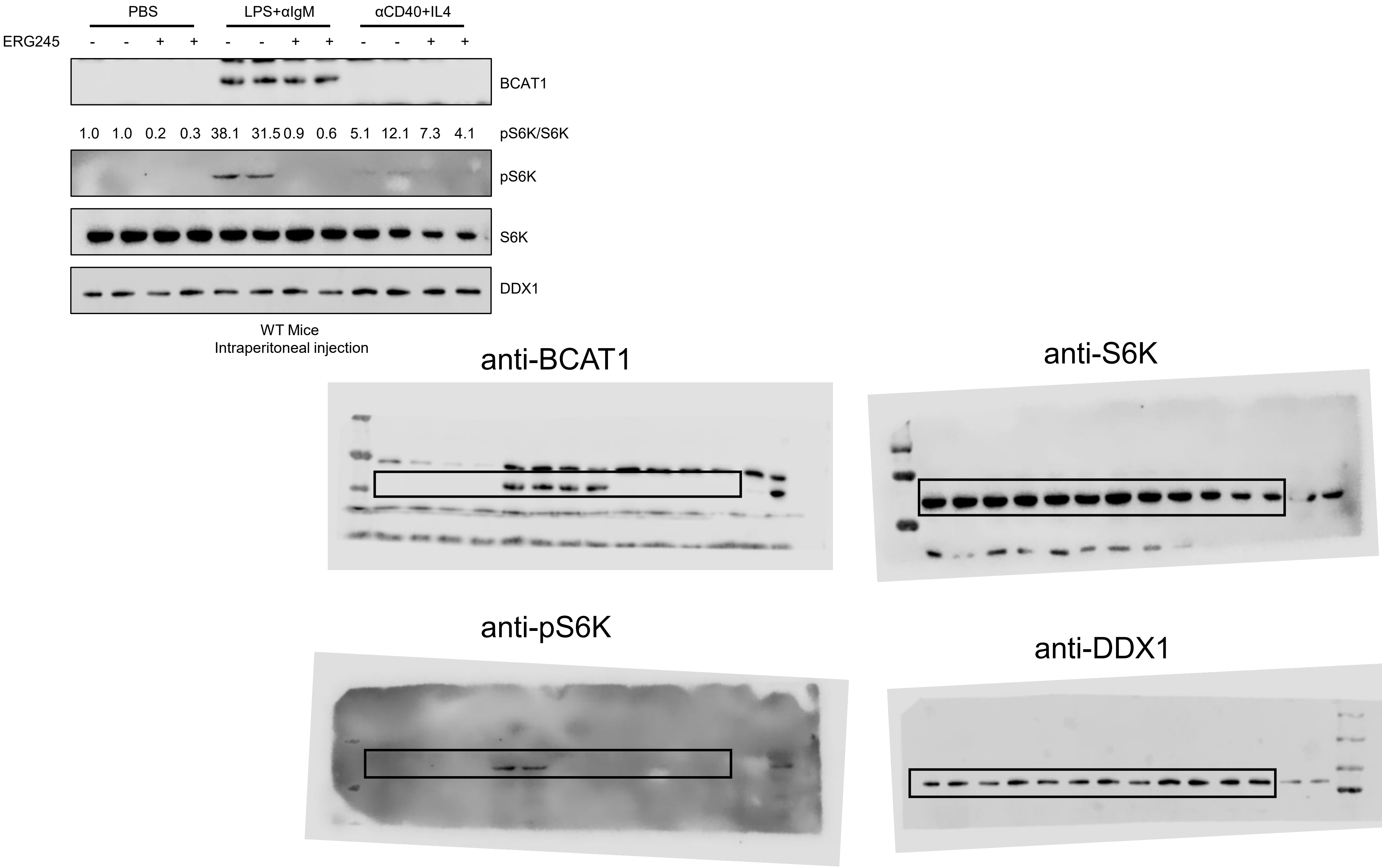
PBS
LPS+αIgM
αCD40+IL4
ERG245
- - + + - - + + - - + +
BCAT1
1.0 1.0 0.2 0.3 38.1 31.5 0.9 0.6 5.1 12.1 7.3 4.1
pS6K/S6K
pS6K
S6K
DDX1
WT Mice
Intraperitoneal injection
anti-BCAT1
anti-S6K
anti-pS6K
anti-DDX1

Fig. 3K

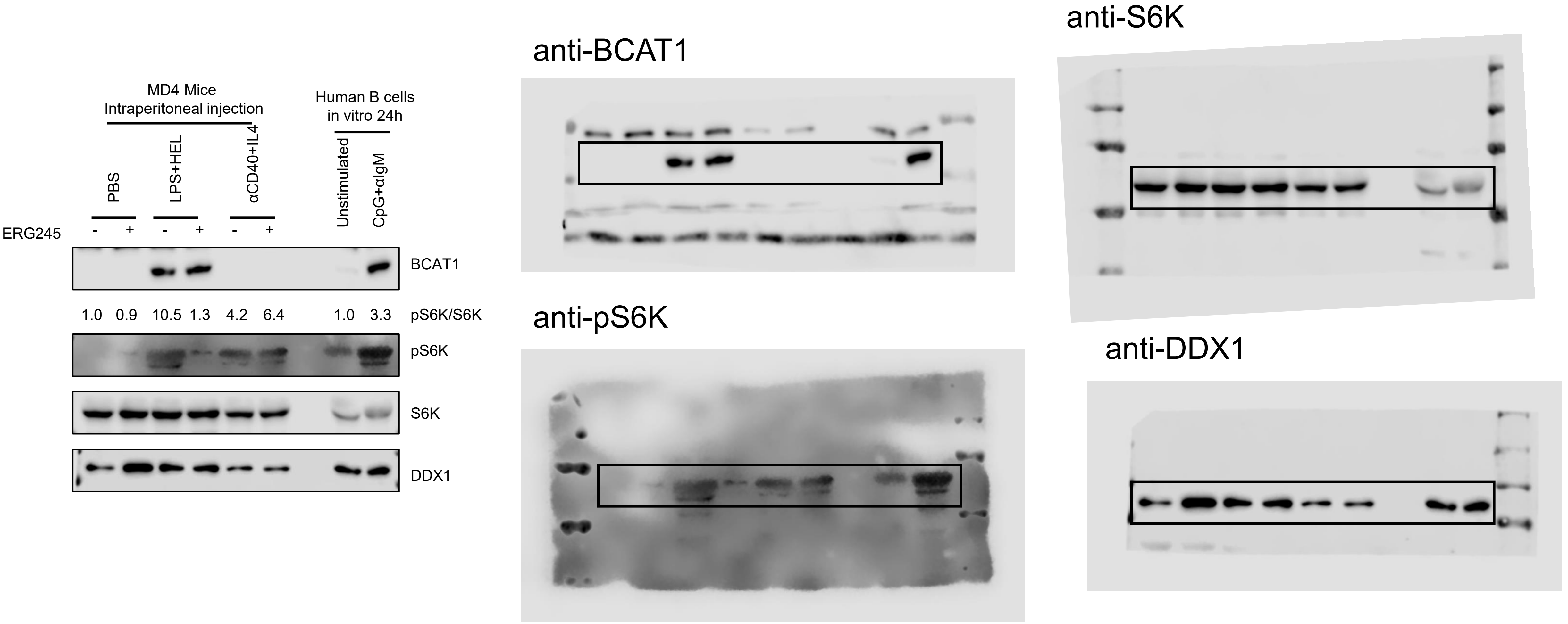
MD4 Mice
Intraperitoneal injection
Human B cells
in vitro 24h
PBS
LPS+HEL
αCD40+IL4
Unstimulated
CpG+αIgM
ERG245
- + - + - +
BCAT1
1.0 0.9 10.5 1.3 4.2 6.4 1.0 3.3
pS6K/S6K
pS6K
S6K
DDX1
anti-BCAT1
anti-pS6K
anti-S6K
anti-DDX1

Fig. 5E

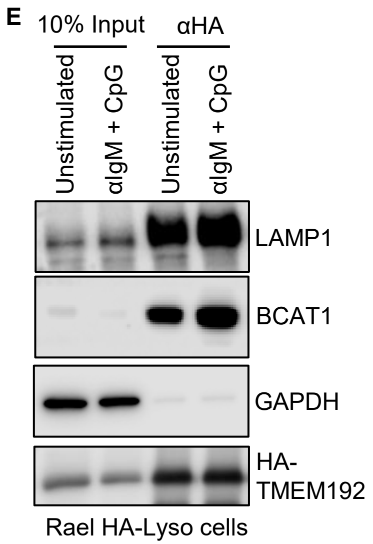

Anti-HA

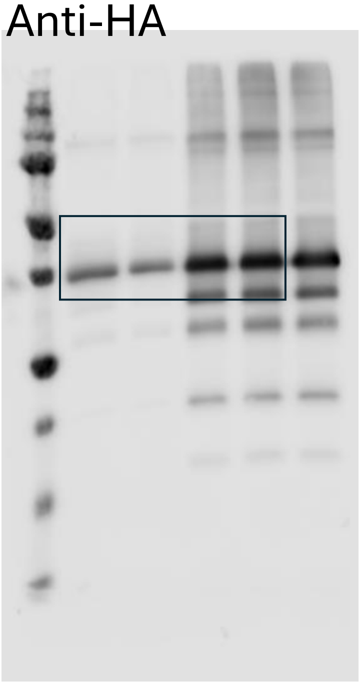

Anti-GAPDH

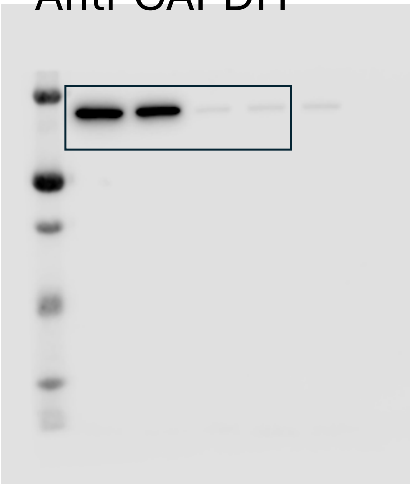

Anti-LAMP1

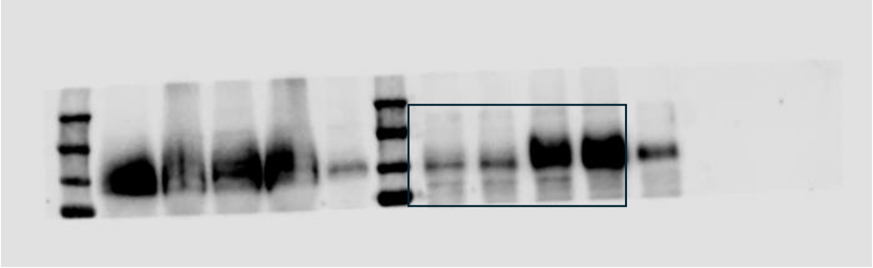

Anti-BCAT1

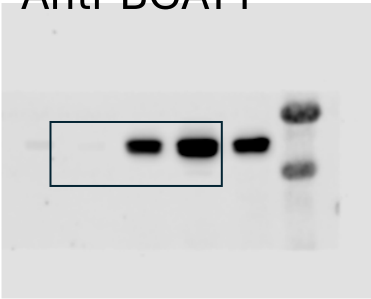

Fig. 6C

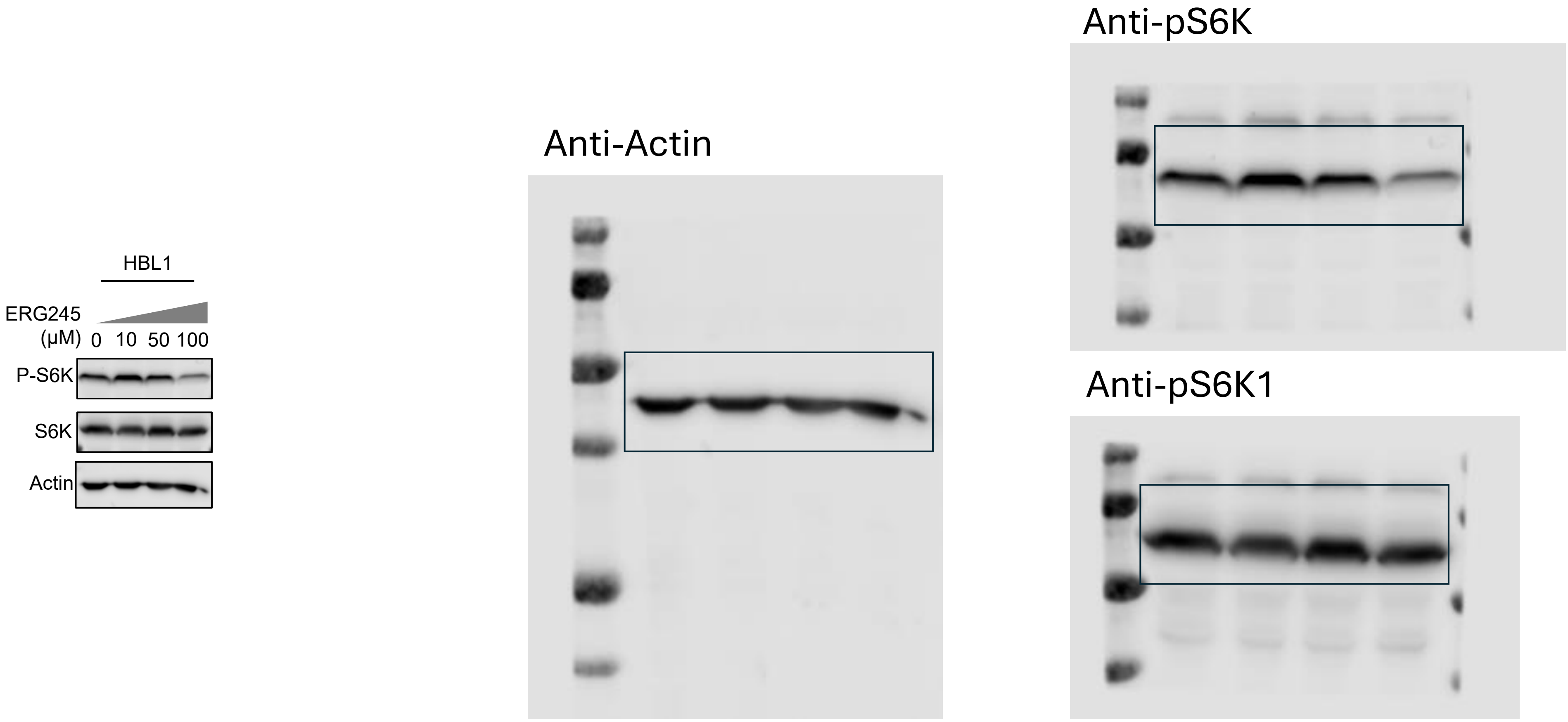

HBL1
ERG245
(μM) 0 10 50 100
P-S6K
S6K
Actin
Anti-Actin
Anti-pS6K
Anti-pS6K1

Fig. 6E

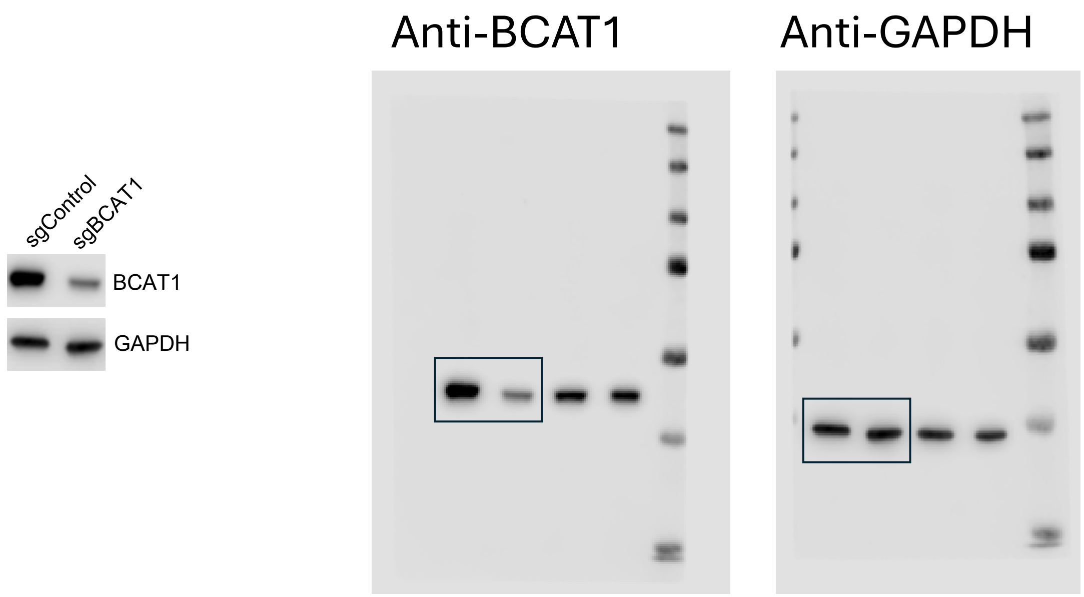

Fig. S1A

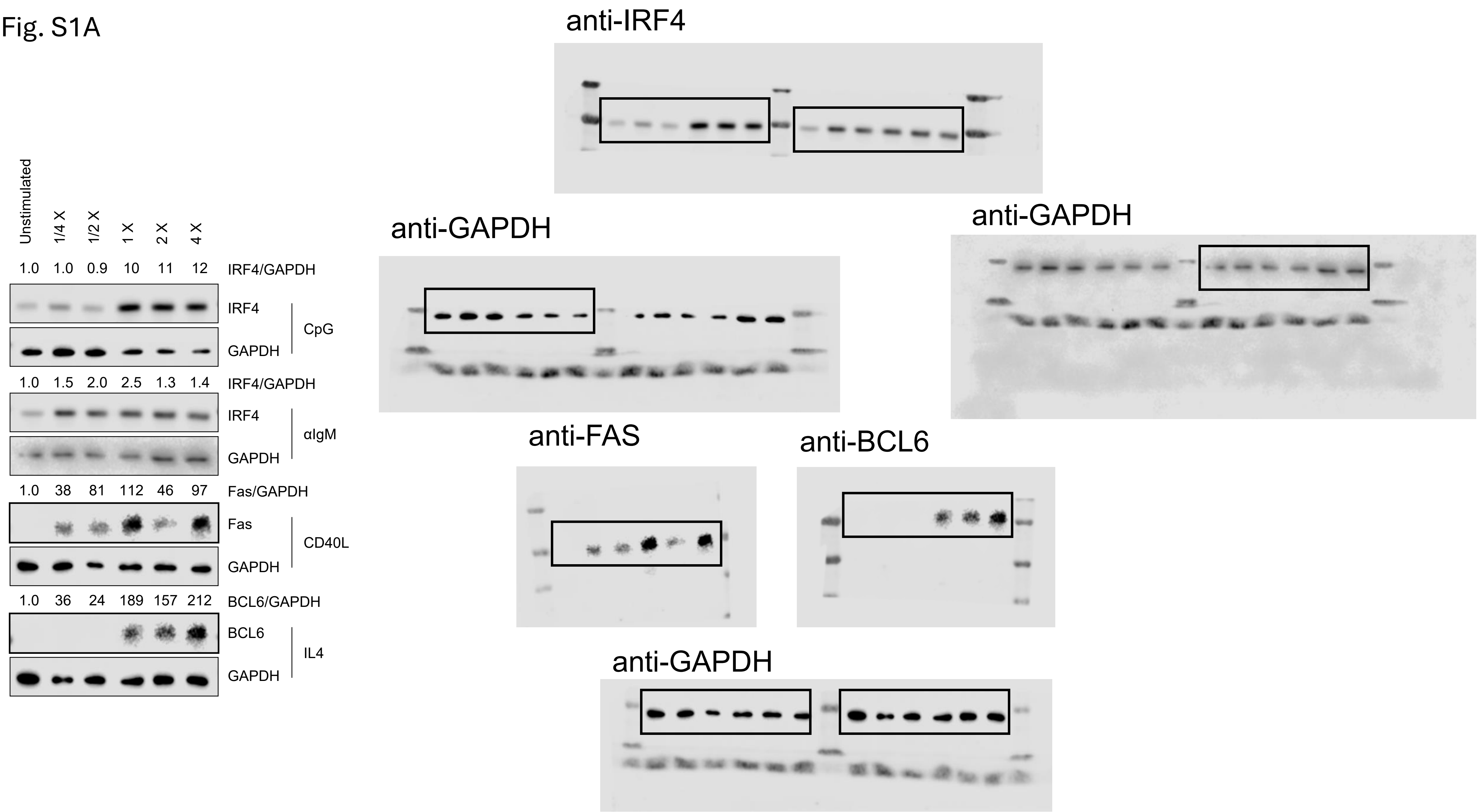
Unstimulated
1/4 X
1/2 X
1 X
2 X
4 X
1.0 1.0 0.9 10 11 12 IRF4/GAPDH
IRF4
GAPDH
CpG
1.0 1.5 2.0 2.5 1.3 1.4 IRF4/GAPDH
IRF4
GAPDH
αIgM
1.0 38 81 112 46 97 Fas/GAPDH
Fas
GAPDH
CD40L
1.0 36 24 189 157 212 BCL6/GAPDH
BCL6
GAPDH
IL4
anti-IRF4
anti-GAPDH
anti-GAPDH
anti-FAS
anti-BCL6
anti-GAPDH

Fig. S1B

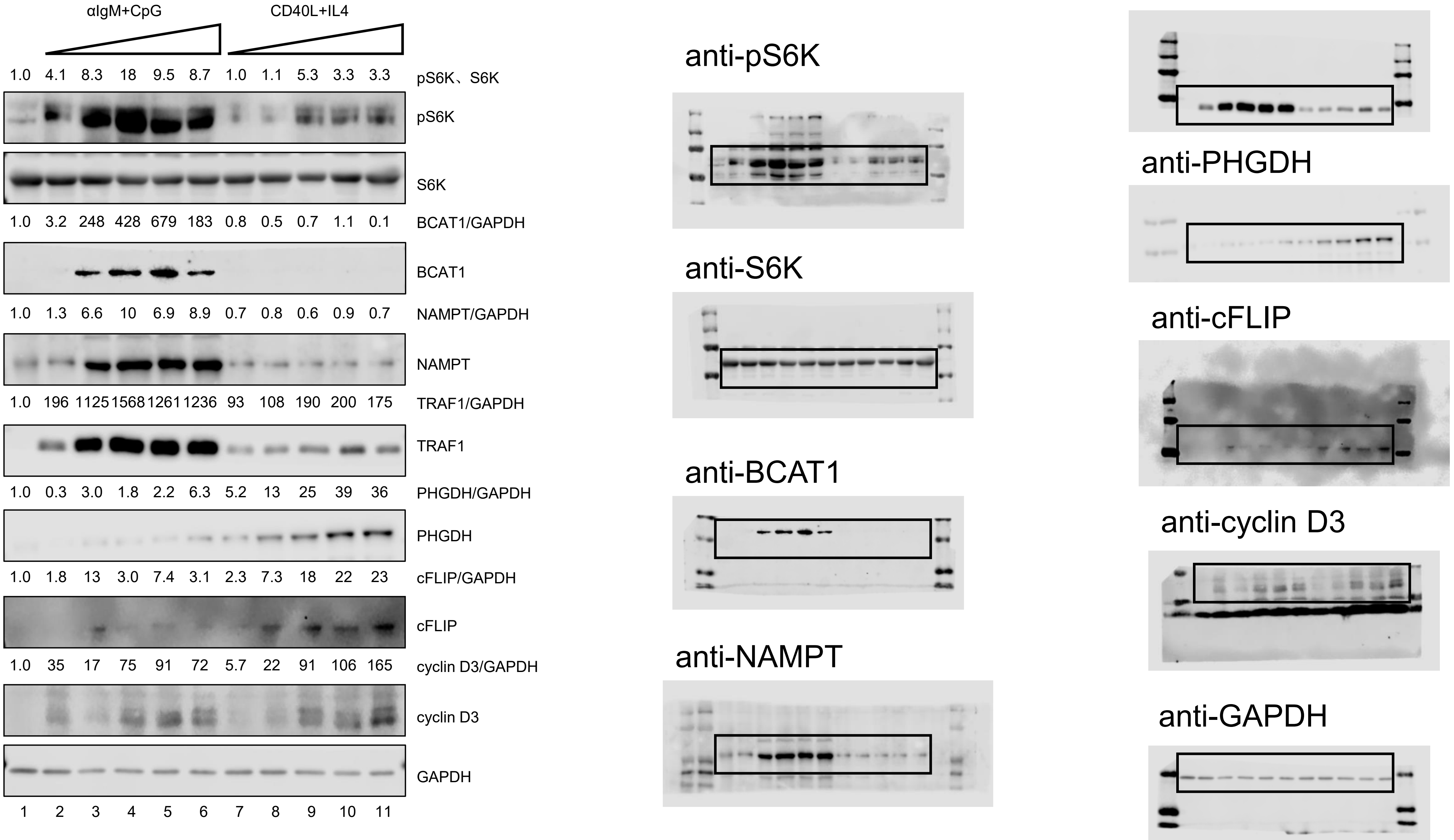

αIgM+CpG
CD40L+IL4
1.0 4.1 8.3 18 9.5 8.7 1.0 1.1 5.3 3.3 3.3 pS6K、S6K
pS6K
S6K
1.0 3.2 248 428 679 183 0.8 0.5 0.7 1.1 0.1 BCAT1/GAPDH
BCAT1
1.0 1.3 6.6 10 6.9 8.9 0.7 0.8 0.6 0.9 0.7 NAMPT/GAPDH
NAMPT
1.0 196 1125 1568 1261 1236 93 108 190 200 175 TRAF1/GAPDH
TRAF1
1.0 0.3 3.0 1.8 2.2 6.3 5.2 13 25 39 36 PHGDH/GAPDH
PHGDH
1.0 1.8 13 3.0 7.4 3.1 2.3 7.3 18 22 23 cFLIP/GAPDH
cFLIP
1.0 35 17 75 91 72 5.7 22 91 106 165 cyclin D3/GAPDH
cyclin D3
GAPDH
1 2 3 4 5 6 7 8 9 10 11
anti-pS6K
anti-S6K
anti-BCAT1
anti-NAMPT
anti-TRAF1
anti-PHGDH
anti-cFLIP
anti-cyclin D3
anti-GAPDH

Fig. S6B

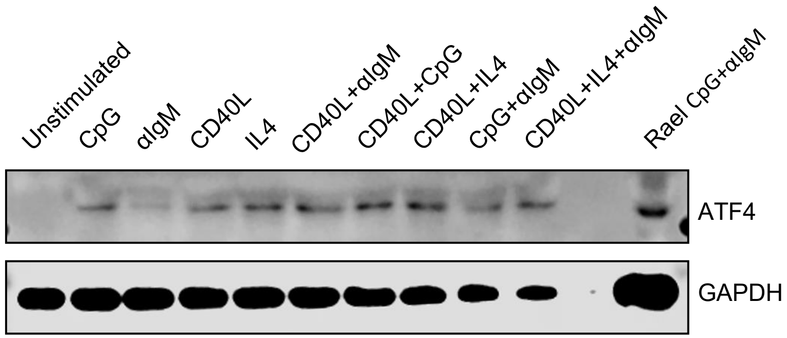

anti-ATF4

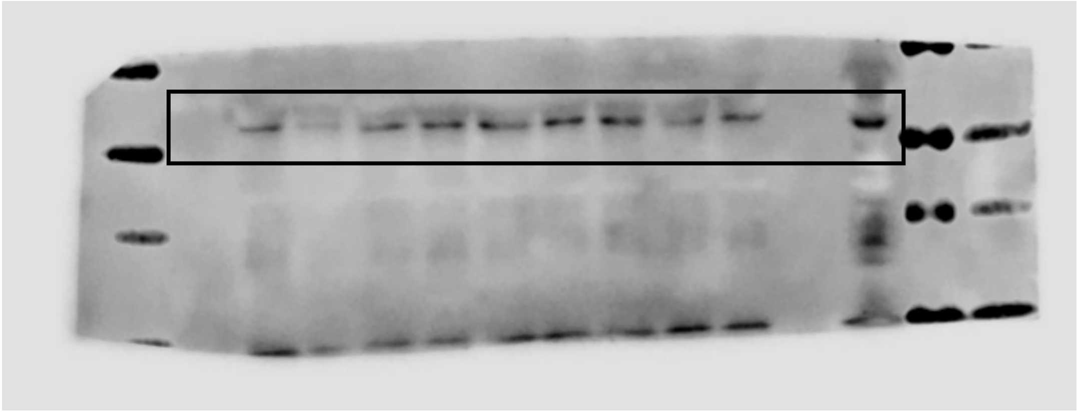

anti-GAPDH

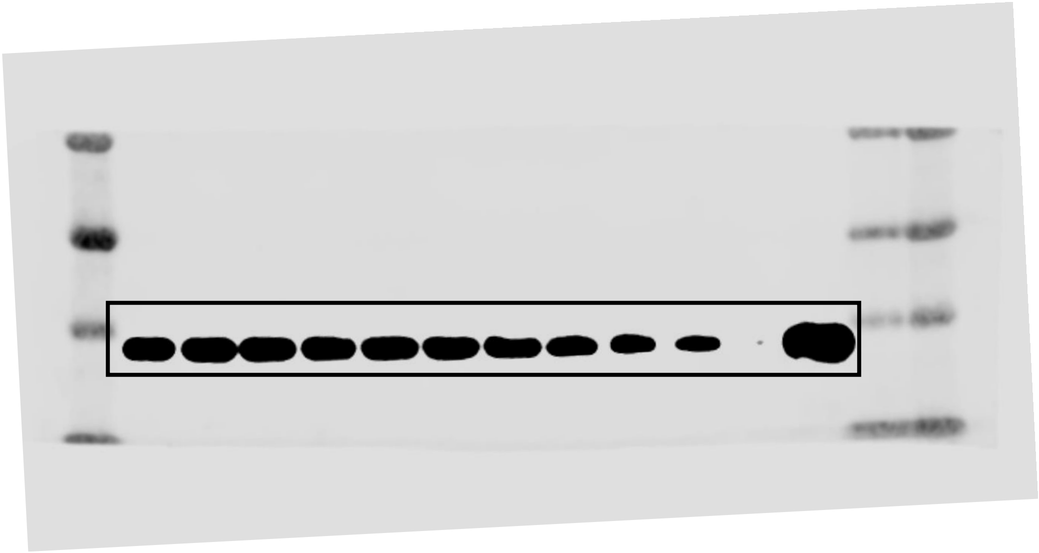

Fig. S6C

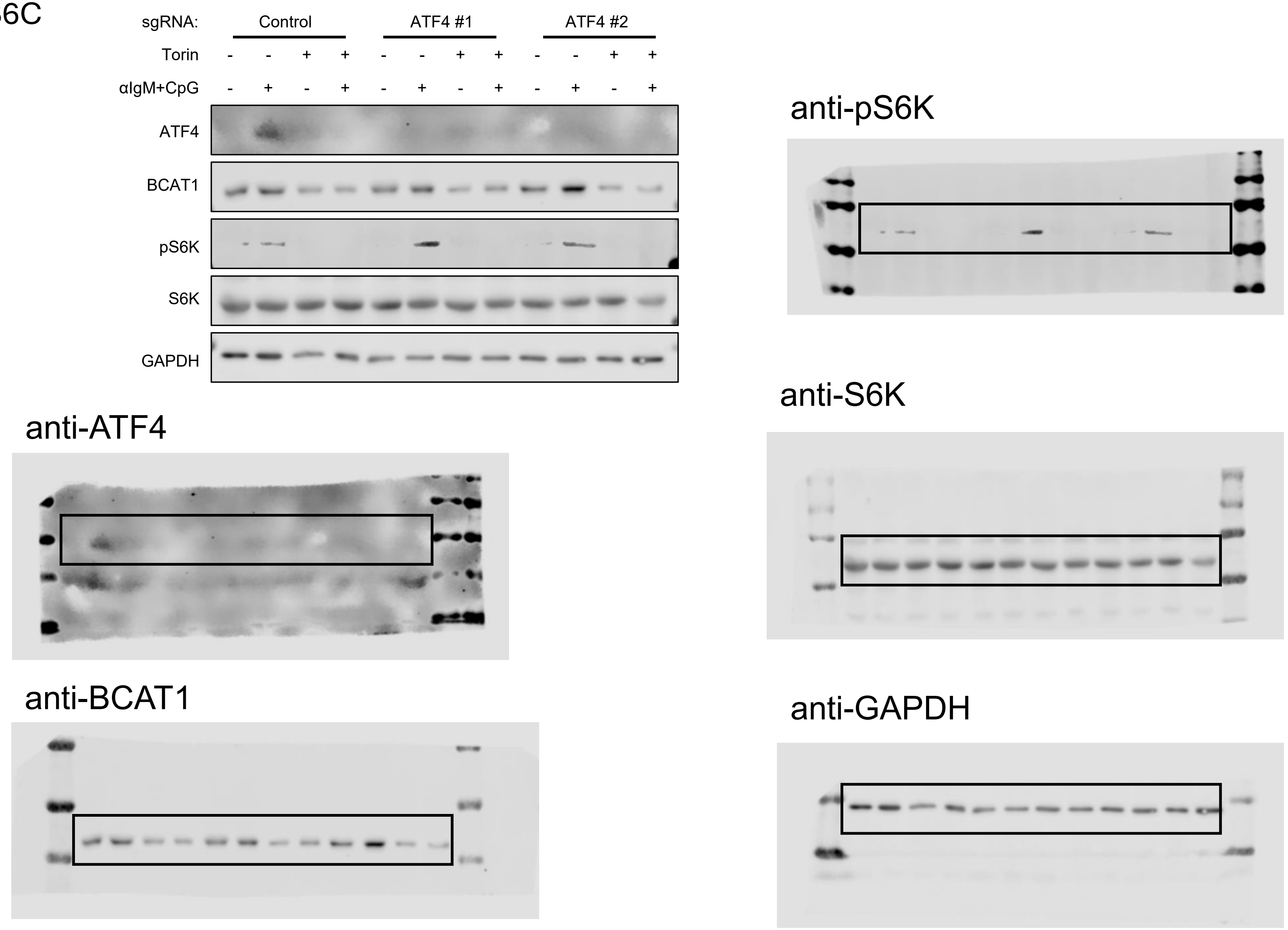

sgRNA:
Control
ATF4 #1
ATF4 #2
Torin
- - + + - - + + - - + +
αIgM+CpG
- + - + - + - + - + - +
ATF4
BCAT1
pS6K
S6K
GAPDH
anti-pS6K
anti-S6K
anti-ATF4
anti-BCAT1
anti-GAPDH

Fig. S7B

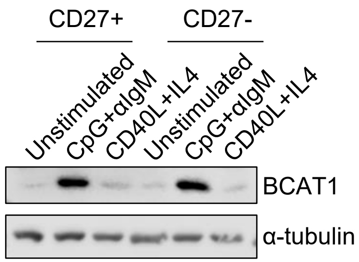

anti-BCAT1

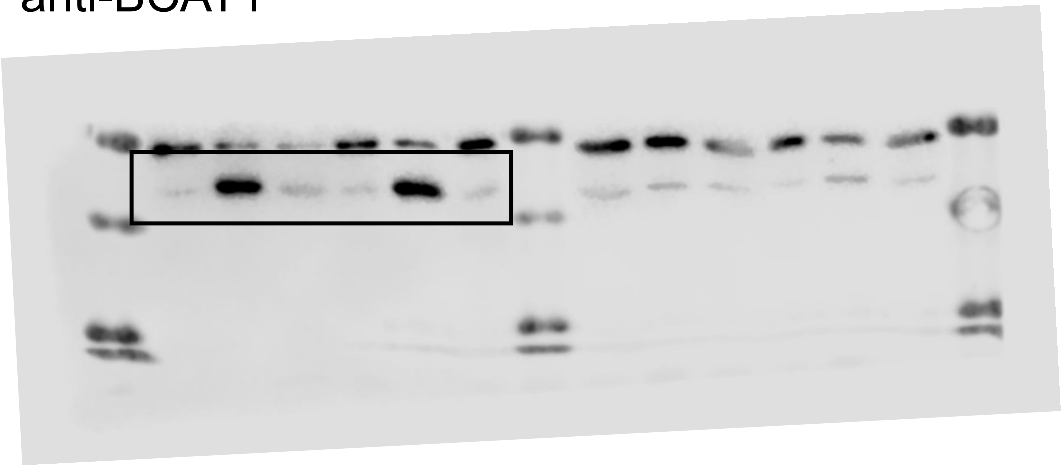

anti-α-tubulin

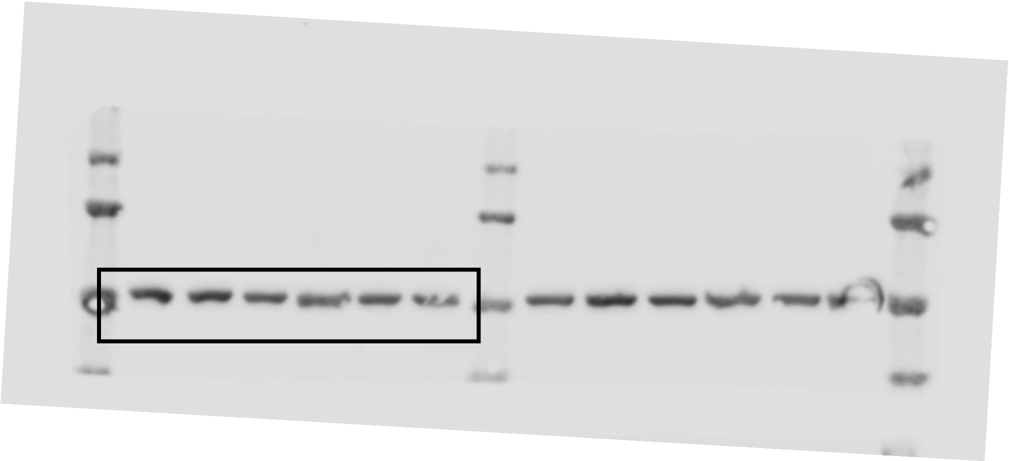

Fig. S8A

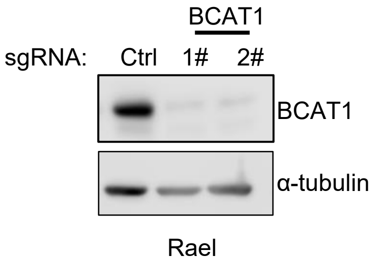

anti-BCAT1

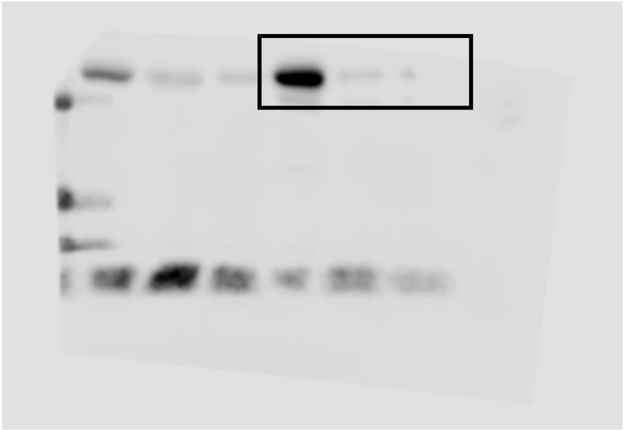

anti-α-tubulin

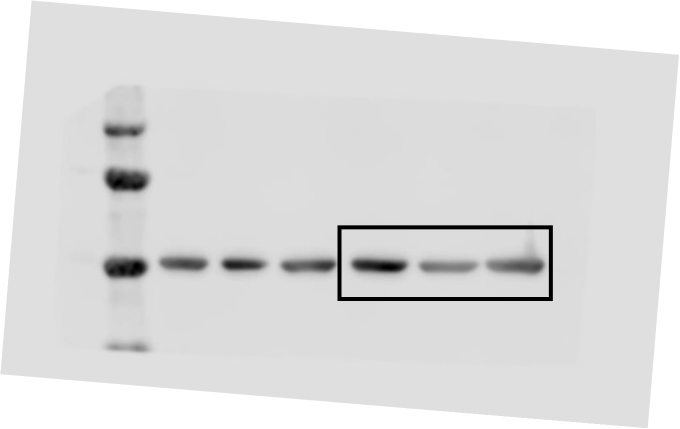

Fig. S9B

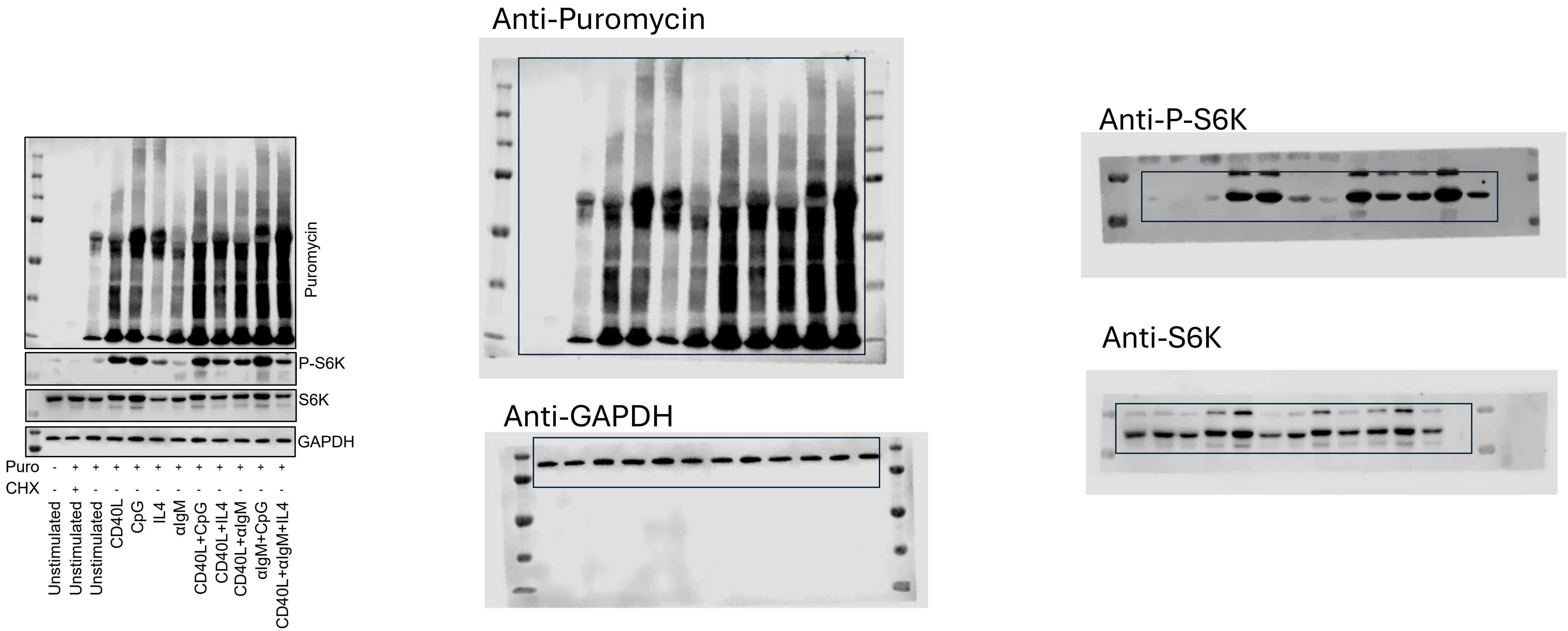
Puromycin
P-S6K
S6K
GAPDH
Puro - + + + + + + + + + + +
CHX - + - - - - - - - - - -
Unstimulated
Unstimulated
Unstimulated
CD40L
CpG
IL4
αIgM
CD40L+CpG
CD40L+IL4
CD40L+αIgM
αIgM+CpG
CD40L+αIgM+IL4
Anti-Puromycin
Anti-GAPDH
Anti-P-S6K
Anti-S6K

Fig. S10A

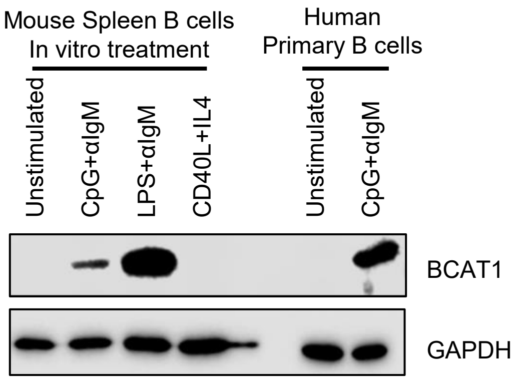

Mouse Spleen B cells
In vitro treatment
Human
Primary B cells
Unstimulated
CpG+αIgM
LPS+αIgM
CD40L+IL4
Unstimulated
CpG+αIgM
BCAT1
GAPDH

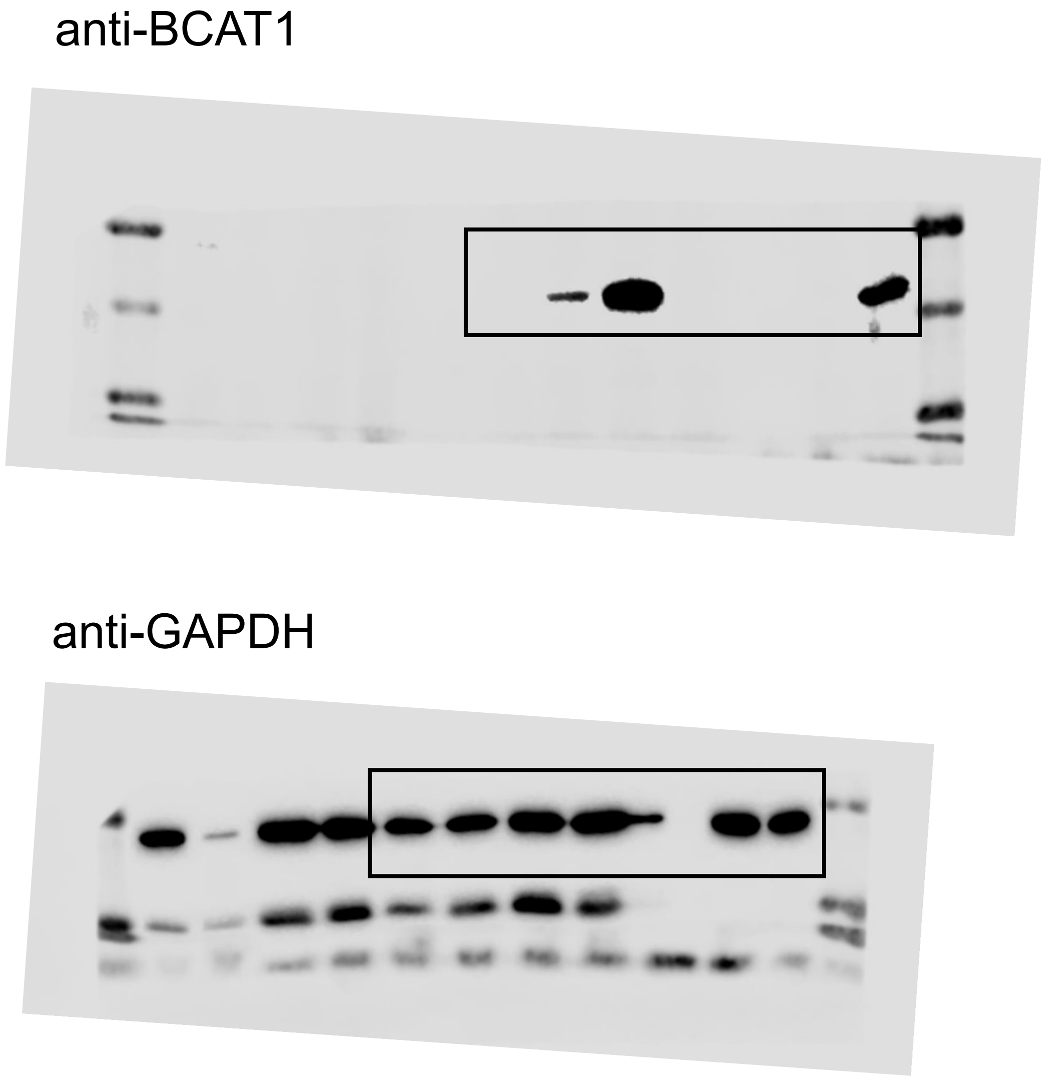

anti-BCAT1
anti-GAPDH

Fig. S10B

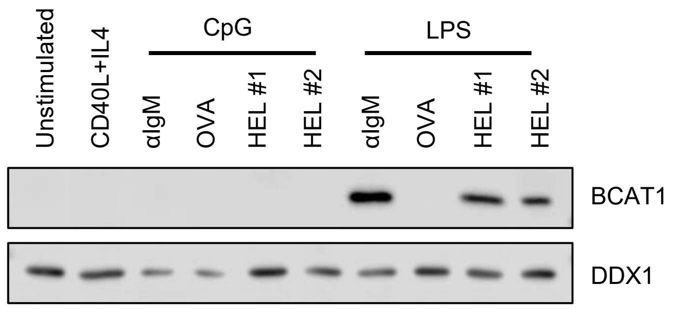

Mouse Spleen B cells
In vitro treatment

anti-BCAT1

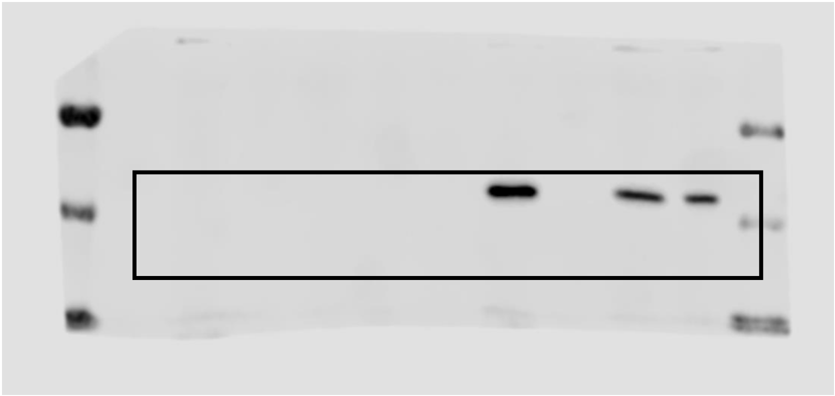

anti-GAPDH

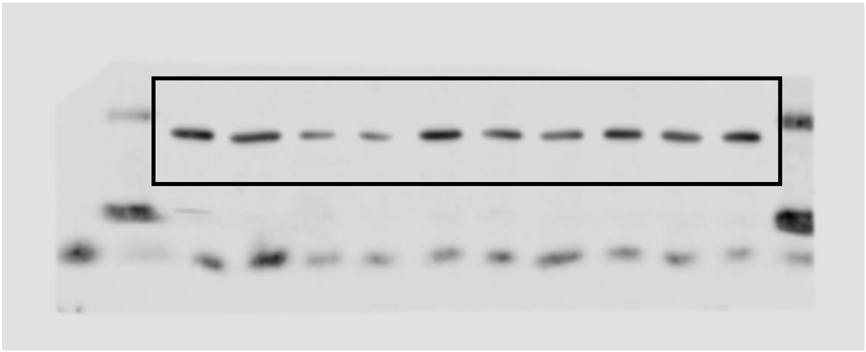

Fig. S11A

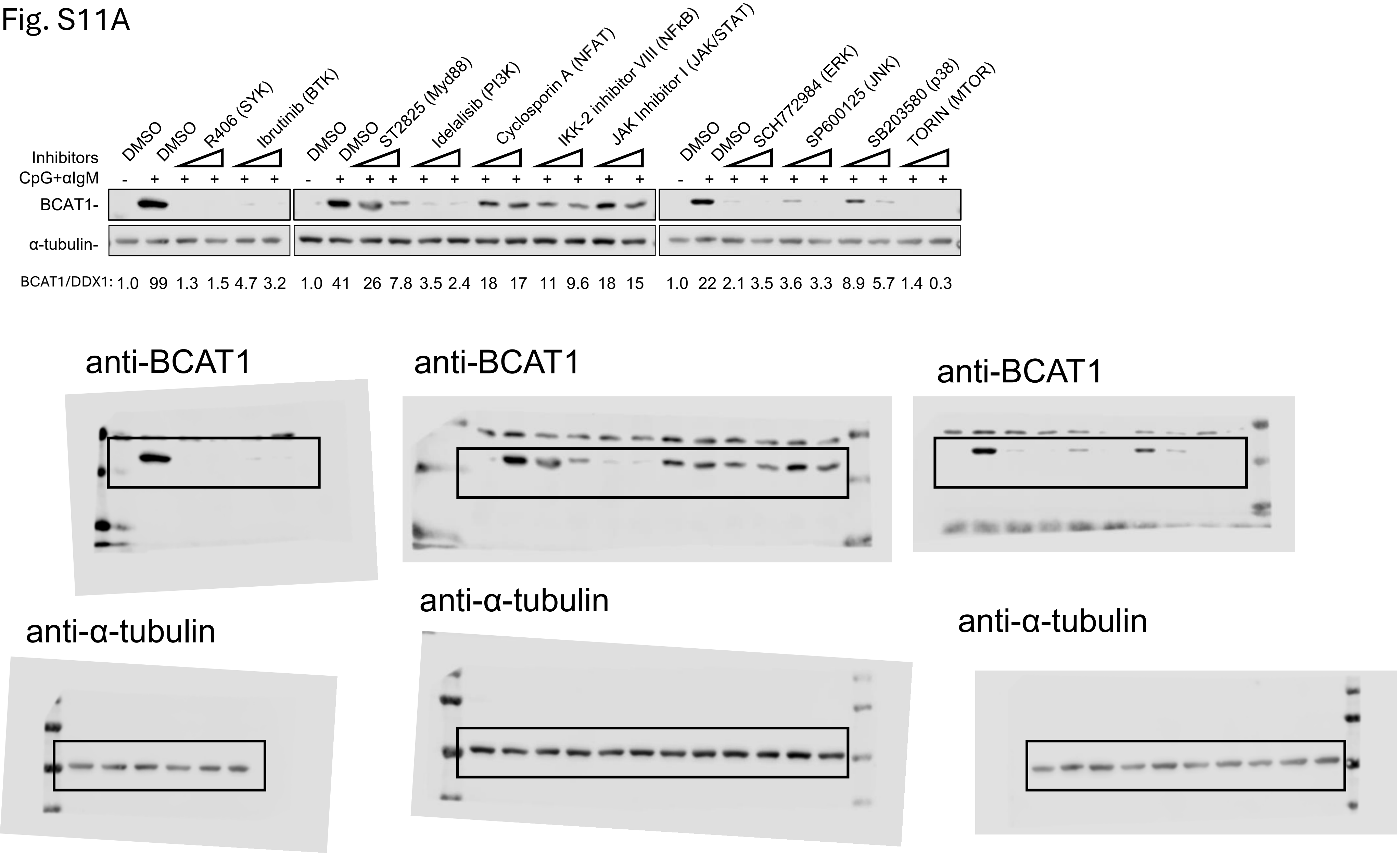
DMSO
DMSO
R406 (SYK)
Ibrutinib (BTK)
DMSO
DMSO
ST2825 (Myd88)
Idelalisib (PI3K)
Cyclosporin A (NFAT)
IKK-2 inhibitor VIII (NFκB)
JAK Inhibitor I (JAK/STAT)
DMSO
DMSO
SCH772984 (ERK)
SP600125 (JNK)
SB203580 (p38)
TORIN (MTOR)
Inhibitors
CpG+αIgM
- + + + + + - + + + + + + + + + + + - + + + + + + + + +
BCAT1-
α-tubulin-
BCAT1/DDX1: 1.0 99 1.3 1.5 4.7 3.2 1.0 41 26 7.8 3.5 2.4 18 17 11 9.6 18 15 1.0 22 2.1 3.5 3.6 3.3 8.9 5.7 1.4 0.3
anti-BCAT1
anti-BCAT1
anti-BCAT1
anti-α-tubulin
anti-α-tubulin
anti-α-tubulin

Fig. S14F

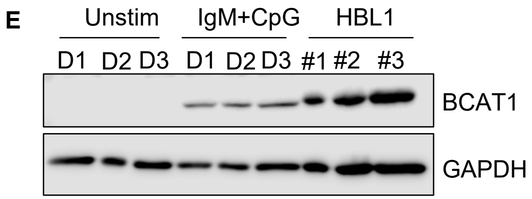

Anti-BCAT1

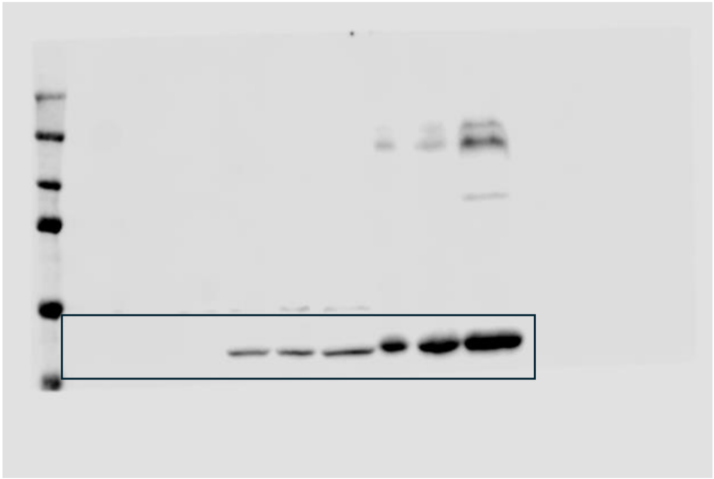

Anti-GAPDH

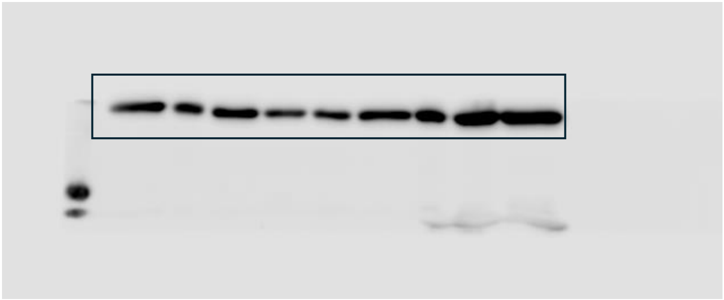

Supplement: Unedited blot and gel images [file jci-135-186258-s038.pdf]
